# Visual marking in mammals first proved by manipulations of brown bear tree debarking

**DOI:** 10.1038/s41598-021-88472-5

**Published:** 2021-05-04

**Authors:** Vincenzo Penteriani, Enrique González-Bernardo, Alfonso Hartasánchez, Héctor Ruiz-Villar, Ana Morales-González, Andrés Ordiz, Giulia Bombieri, Juan Diaz García, David Cañedo, Chiara Bettega, María Del Mar Delgado

**Affiliations:** 1 Research Unit of Biodiversity (UMIB, CSIC-UO-PA), Mieres Campus, 33600 Mieres, Spain; 2 Pyrenean Institute of Ecology (IPE), C.S.I.C., Avda. Montañana 1005, 50059 Zaragoza, Spain; 3 FAPAS Fondo para la Protección de los Animales Salvajes, Ctra. AS-228, km 8,9 – Tuñón, 33115 Santo Adriano, Asturias Spain; 4 Department of Conservation Biology, Estación Biológica de Doñana, C.S.I.C, Avda. Americo Vespucio 26, 41092 Sevilla, Spain; 5 Faculty of Environmental Sciences and Natural Resource Management, Norwegian University of Life Sciences, Postbox 5003, NO-1432, Ås, Norway; 6 MUSE - Museo delle Scienze, Sezione Zoologia dei Vertebrati, Corso del Lavoro e della Scienza 3, 38123 Trento, Italy; 7 Consejería de Ordenación del Territorio, Infraestructuras y Medio Ambiente, Dirección General de Biodiversidad, Oviedo, Principado de Asturias Spain

**Keywords:** Ecology, Zoology

## Abstract

The rather limited human ability to understand animal vision and visual signalling has frequently clouded our expectations concerning the visual abilities of other animals. But there are multiple reasons to suspect that visual signalling is more widely employed by animals than previously thought. Because visibility of visual marks depends on the background in which they are seen, species spending most of their time living in dark conditions (e.g., in forests and/or having crepuscular and nocturnal habits) may rely on bright signals to enhance visual display. Here, as a result of experimental manipulations, we present, for the first time ever, evidence supporting the use of a new channel of intraspecific communication by a mammal species, i.e., brown bear *Ursus arctos* adult males relying on visual marks during mating. Bear reactions to our manipulation suggest that visual signalling could represent a widely overlooked mechanism in mammal communication, which may be more broadly employed than was previously thought.

## Introduction

Among the many groups of terrestrial species, our understanding of mammal visual signalling might be hampered by the fact that most research on mammals has focused on chemical (e.g., scat, urine, and glands) and acoustic (e.g., howling) signalling^1,2^. Instead^2,3^, visual communication might be an overlooked communication channel^2,4^, despite being perhaps as important as the others, if we consider that: (1) mammal coloration has evolved for inter- and intraspecific communication^2,4–7^, which means that mammals use visual signals to communicate; and (2) visual signalling through physical marks (e.g., bites and scratches) is permanent and, thus, has the obvious advantages of (a) being long-lasting, i.e., environmental factors such as rain or snow are less likely to affect the detectability of visual marks as compared to, e.g., chemical signalling^8^, although mammals have found strategies to make chemical signalling last as long as possible^9^, and (b) functioning remotely, i.e., even when the signaller is away from the marked location^2^. Visual marking may also allow individuals to reduce repeated visits to strategic marking points, and thus save time and energy, which would otherwise detract animals from other activities, like foraging and reproduction^10^. Therefore, visual signalling may represent a reliable and advantageous communication channel^8^.

Solitary species like bears may benefit from advertising their location, size, and reproductive status to expedite mate selection during the breeding season. Moreover, brown bears usually occur at low densities across their range, making direct interactions with one another infrequent^11,12^. Thus, long-lasting visual signalling may be particularly effective and considerably time saving. To date, studies on bear communication have highlighted two main forms of communication^10,13–17^: (1) olfactory communication, i.e., the marking of focal trees by rubbing the body against the trunk and/or by urination and deposition of anogenital gland secretions; and (2) pedal marking, by which bears mark the ground with their scent by grinding their feet into the substrate. Auditory communication, e.g., vocalizations used as threats during agonistic encounters, to advertise sexual receptivity, or for communication between females and their cubs, is considered as the least important channel through which bears signal, whereas visual communication has always been considered limited to different forms of body postures or behavioural displays (but see^18^).

Since the beginning of the 1980s, bear marks on trees have puzzled researchers^8^. The function of, and motivation behind, tree biting and clawing have prompted a variety of theories related to glandular scent deposition (i.e., chemical signalling), but none of these hypotheses has been considered satisfactory, nor have they ever been tested^8^. The debarking behaviour of brown bears *Ursus arctos*, which leaves bright and conspicuous marks on tree trunks (see Extended Data Fig. 1 and Extended Data Fig. 2), presents a unique yet unexplored opportunity to investigate new ways of visual communication in terrestrial mammals, and to better understand both bear and carnivore communication broadly. The hypothesis behind this experimental work is that brown bears may rely on visual communication via the conspicuous marks that they produce on trees.

**Figure 1:**
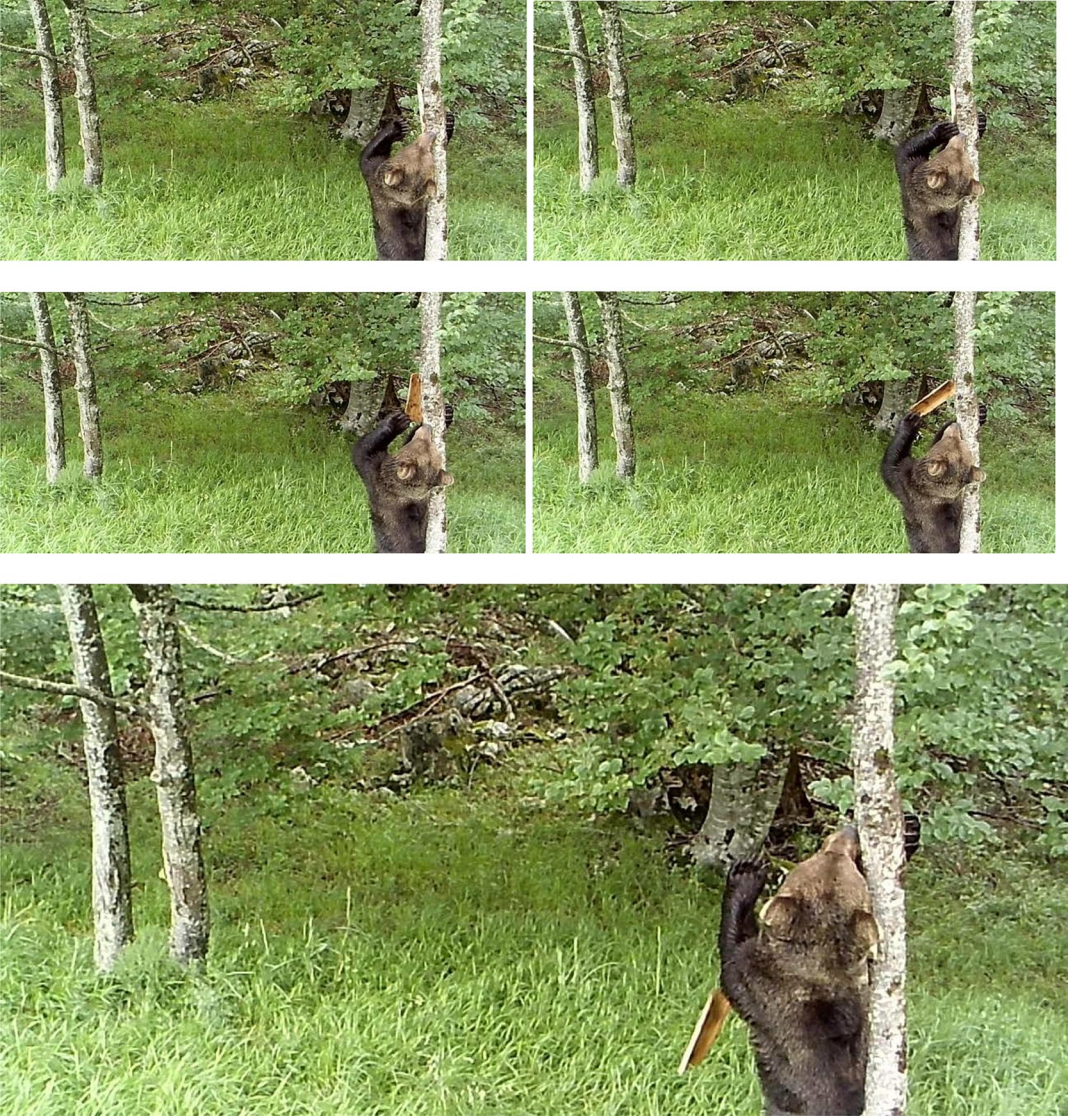
Brown bear response to trunk mark manipulation. The behavioural sequence of an adult male brown bear removing the pieces of bark that we used to conceal the visual markings on an ash tree during the mating season in the Cantabrian Mountains, Spain (12/06/2020, 15h37). The whole sequence is shown in the video footage Extended Data Fig. 5.

After manipulating bear tree marks in the Cantabrian Mountains (north-western Spain), we found that bears removed the bark strips that we used to cover their marks during the mating season (Extended Data Figs. 3 and 4), suggesting that bear debarking may represent a visual communication channel used for intraspecific communication.

### Brown bear responses to marked tree manipulations

After concealing bear marks due to trunk debarking with bark strips from the same tree species (see “Methods”), our manipulations on 20 trees triggered a rapid reaction from brown bears. Between the 16th of May and the end of September 2020 (overlapping part of the brown bear mating period in the Cantabrian Mountains^19^), brown bears removed the strips of bark that we used to cover the trunk marks in 9 (45%) out of the 20 manipulated trunks (Fig. 1 and Extended Data Fig. 5). However, if we consider that these nine trees were also the ones that we could manipulate (because of field work restrictions due to COVID-19) from the start of the mating season (beginning of May), 100% of the bark strips used to cover tree marks were removed by bears when the manipulation occurred at the commencement of the mating season. In only one case, a bear removed the bark strips covering marks on a tree that was manipulated later in the mating season (end of June). Control bark strips fixed to (a) the same trunk as the manipulated bear mark, (b) the nearest neighbouring tree to the manipulated one showing bear marks, and (c) the nearest rubbing trees with no bear marks, were never removed by bears. In two cases (50%), after the first removal of the manipulated mark by a bear, which was subsequently covered again with new strips (n = 4), a bear removed the strips a second time. Further, camera traps showed that: (1) bears uncovered the manipulated marks the first time they visited the tree after our manipulation; (2) bark strips that were not removed were always the result of bears not visiting the site after tree manipulations; and (3) the shortest lapse of time between a mark manipulation and a bear visiting the tree for the first time and uncovering the mark was seven days. Thus, manipulations always triggered a rapid response from bears when adult males, probably the same individuals that debarked the trunks, came back and check on marked trees.

### Conspicuousness of brown bear visual marks

The conspicuousness of a visual signal is not only increased by its position in a noticeable location, but also by the contrast between the signal and its background^20,21^. A remarkable difference (pixel intensity: mean (± SD) = 85.09 ± 26.77, range = 20.27–177.06) exists between bark and sapwood brightness for all tree species (*t* = 19.07, *p* =  < 2.2e−16) (Extended Data Fig. 1). Even if contrast values for certain tree species, such as linden *Tilia platyphyllos* (*p* = 0.05) and hazel *Corylus avellana* (*p* = 0.09), were considerably higher than those for the rest of the species, the debarked tree species showed no remarkable differences in contrast among them (*F* = 1.11, *p* = 0.39, R^2^ = 0.03), which suggests that a debarked tree is always conspicuous, independent of bark colour.

### Tree species selection for marking purposes

Debarked trees belonged to species relatively scarce in forest stands, i.e., only 31.1% ± 29.4 of the trees recorded in the proximity of a marked tree (see “Tree species selection for marking purpose” in “Methods”) were of the same species as the trees marked by bears. Moreover, in only 19 of the 59 covered transects (33.90%), the tree species marked by bears was the most abundant one. These percentages decrease if we remove a single monospecific forest stand of planted Monterey pine *Pinus radiata*. Indeed, if we only take into account native forest stands: (a) only 26.2% ± 26.2 (range = 0–85.7%) of the trees recorded in the proximity of the marked tree corresponded to the same species as the tree marked by a bear; and (b) in only 26.4% of transects, the tree species marked by bears was the most abundant one. This suggests that bears may select for some tree species, probably because of the characteristics of their bark, e.g., softness^22^.

Dominant males use chemical signalling to communicate and maintain dominance over other males and, consequently, subordinate males have been shown to scent-mark less than dominant males and in some cases not scent-mark at all^14–16^. Our two-year video recordings (Extended Data Figs. 3, 4 and 7) show analogies between chemical and visual signalling, the latter being also mainly performed by adult males during the mating season.

Interestingly, clawing and biting the bark of a tree, often leaving fur, frayed bark and scars on the tree trunk or other substrates, have always been considered olfactory signals^10,23^. For example, it has been suggested that clawing may leave scent from pedal glands and biting may deposit saliva^10^. Yet, at least for brown bears, the amount of smell left by scratches and bite marks on trees is expected to be less than that left by secretions from sebaceous and apocrine glands when rubbing the whole body^10^ and, thus, might result in an unnecessary reinforcement of body rubbing. Moreover, visual marks are generally on the upper sections of the tree, which can only be reached by larger adult males, and furthermore they would not be reached while body rubbing (Extended Data Fig. 9). This may explain why adult males use multiple marking behaviours to leave two different signals, i.e., chemical and visual, which may complement each other^8^. For example, whereas a chemical signal provides information on bear sex and individuality, visual marks might simultaneously indicate the height of the bear, thus providing a signal that is physically associated with a quality of interest to the receiver^24^. A similar behaviour has been suggested for tigers, *Panthera tigris*, which mark their territories by scratching as high as they can on tree trunks, a signal physically connected to their size^25^. It has also been hypothesised that visual marks simply identify the location of chemical signalling^8^. However, we believe that this may not always be the case, since: (1) visual marks do not necessarily happen on trees where body rubbing and pedal marking occur (Extended Data Figs. 1 and 8); and (2) a visual mark on a tree in a forest is only visible when the receiver is close to the mark, whereas chemical signals may go farer (e.g. by wind action) and reach an animal before a visual one.

Our results suggest that trunk debarking by brown bears plays an important role in visual communication at least during the mating season. In turn, visual signalling may be related to individual fitness, because communication is the first step towards successful mating and eventual reproduction.

This is the first time, to our knowledge, that the active role of visual marking in a mammalian species was experimentally tested in the field. To conclude, bear reactions to our manipulation suggest that visual signalling could represent a widely overlooked mechanism in mammal communication, which may be more broadly employed than was previously thought.

## Methods

### Manipulation of trunk debarking

Twenty trees with brown bear marks on their trunks^26^ were used for bark manipulations from the 1st of May 2020 (the beginning of the mating period in the Cantabrian Mountains^19^) to the end of September 2020 (the beginning of the hyperphagia period in this area^19^, when trunk marking is supposed to stop or, at least, to decrease^8^). Strips of bark of the same species as the marked trees were used to cover bear marks (Extended Data Fig. 6). We collected strips from the ground or we debarked a distant (preferably recently died) tree to avoid any further interaction with the trees marked by bears. Control bark strips were used on: (a) the same trunks as the manipulated bear marks, (b) the nearest tree of the same species as the manipulated one, and (c) the nearest rubbing trees with no bear marks. Control strips were used to discard the possibility that brown bears were attracted by our scent and removed the strips for any reason other than to uncover their visual marks. Additionally, in four cases where a bear removed the mark manipulation, it was possible to cover the bear mark again to reinforce support for the importance of visual signalling in brown bears. All manipulated trees were checked approximately every 15 days.

In five of the manipulated trees camera, traps were deployed (Browning Dark Ops HDProX) from May to August 2020. This period has been considered the one in which debarking is most intense in bears^8^. Camera traps were programmed to record, when triggered by an animal, one-minute videos during the day, and 20-s videos at night, with a one-second trigger delay between videos. All sites were visited every two weeks to check if the bark manipulations had been removed and to service camera traps (e.g. battery check, eventually stolen cameras). Additionally, to document brown bear debarking behaviour away from of our manipulations, from January 2019 to July 2020 six additional camera traps were deployed to monitor six previously known rubbing trees highly frequented by bears, but where no visual marks were found (e.g., Extended Data Fig. 3).

### Conspicuousness of brown bear visual marks

To explore the possibility that brown bear tree marking is a conspicuous signal on a trunk, we measured the contrast between the barkand sapwood for each of the marked tree species, as a proxy of mark brightness and conspicuousness. Using a blade, we first removed a small section of bark (approximately 3 × 4 cm, outer and inner bark) from three different trunks for each tree species. Bark removal exposed the sapwood, as happens in brown bear debarking. We took a total of 36 tree photos (JPEG format, 7 MG each), corresponding to 3 individuals from each of the 12 tree species where visual marking was detected: sycamore maple *Acer pseudoplatanus*, hazel *Corylus avellana*, birch *Betula pubescens*, chestnut *Castanea sativa*, cherry *Prunus avium*, ash *Fraxinus excelsior*, beech *Fagus sylvatica*, whitebeam *Sorbus aria*, Monterey pine *Pinus radiata*, oak *Quercus petraea*, willow *Salix caprea*, and linden *Tilia platyphyllos*. For repeatability purposes^18,27^, we took six measurements of bark brightness (three measurements of the bark and three of the exposed sapwood) for each picture. Finally, we calculated mean brightness values for both the cortex and the sapwood, and afterwards we calculated the contrast value (i.e., brightness of the bark − brightness of the sapwood) for each picture, for statistical purposes. Brightness values were obtained by processing the images with the Java-based image processing program ImageJ (https://imagej.nih.gov/ij/), by means of the Oval Selection Tool (width = 200 pixels, height = 200 pixels) and the Measure Tool. Digital images are two-dimensional grids of pixel intensity values with the width and height of the image being defined by the number of pixels in x (rows) and y (columns) directions. Thus, pixels (picture elements) are the smallest single component of digital images, holding numeric values (pixel intensities) that range between black and white. RGB pixels are converted to brightness values using the formula = (red + green + blue)/3 (ImageJ User Guide IJ 1.46r, http://imagej.nih.gov/ij/docs/guide).

### Tree species selection for marking purposes

To study whether brown bears might select specific trees on which to leave visual marks because, e.g., of the conspicuousness of the mark and/or the ease of debarking, we used a set of 59 debarked trees previously recorded in the Cantabrian Mountains^26^ to walk 59 linear transects with the aim of comparing the frequency of the tree species debarked by bears *vs*. the abundance of each tree species around the marked tree. Each transect had a total length of 40 m (20 m up and 20 m down from the marked tree), and the total number of trees of each species was recorded. The mean (± SD) number of trees (all species together) recorded was 13.0 ± 6.7 (range = 1–30 trees).

### Statistical analyses

We first compared the average brightness of bark with the average brightness of sapwood (n = 36 pictures) using a paired *t-test* (α = 0.05). Second, to assess the variation in contrast among tree species, we built a linear model with contrast as the response variable, and species as the explanatory variable. Analyses were performed in R 3.5.1 statistical software^28^.

## Supplementary information

Supplementary Figure 1.

Supplementary Figure 2.

Supplementary Figure 3.

Supplementary Figure 4(1).

Supplementary Figure 4(2).

Supplementary Figure 4(3).

Supplementary Figure 5.

Supplementary Figure 6.

Supplementary Figure 7.

Supplementary Figure 8.

Supplementary Figure 9.

Supplementary Legends.
