# Supplementary material for: Visual marking in mammals first proved by manipulations of brown bear tree debarking
Source: Sci Rep. 2021 May 4;11:9492. doi: 10.1038/s41598-021-88472-5 (PMC8096968; doi:10.1038/s41598-021-88472-5)

Visual marking in mammals first proved by manipulations of brown bear tree debarking

Vincenzo Penteriani[1*¶], Enrique González-Bernardo[1,2¶], Alfonso Hartasánchez[3], Héctor Ruiz-Villar[1], Ana Morales-González[4], Andrés Ordiz[5], Giulia Bombieri[6], Juan Díaz García[7], David Cañedo[7], Chiara Bettega[1], María del Mar Delgado[1]

1. Research Unit of Biodiversity (UMIB, CSIC-UO-PA), Mieres Campus, 33600 Mieres, Spain

2. Pyrenean Institute of Ecology (IPE), C.S.I.C., Avda. Montañana 1005, 50059 Zaragoza, Spain

3. FAPAS Fondo para la Protección de los Animales Salvajes, Ctra. AS-228, km 8,9 – Tuñón, 33115 Santo Adriano, Asturias, Spain.

4. Estación Biológica de Doñana, C.S.I.C., Department of Conservation Biology, Avda. Americo Vespucio 26, 41092 Sevilla, Spain

5. Faculty of Environmental Sciences and Natural Resource Management, Norwegian University of Life Sciences, Postbox 5003, NO-1432, Ås, Norway

6. MUSE - Museo delle Scienze, Sezione Zoologia dei Vertebrati, Corso del Lavoro e della Scienza 3, I-38123, Trento, Italy.

7. Consejería de Ordenación del Territorio, Infraestructuras y Medio Ambiente, Dirección General de Biodiversidad, Principado de Asturias, Oviedo, Spain

*Correspondence author: v.penteriani@csic.es

¶ These authors contributed equally to this work

Extended Data Fig. 2 | Old (i.e., previous mating season) visual marks left by brown bears. Different examples of old (> 1 year old) brown bear visual markings on diverse tree species. Even if the contrast between the outer bark and the interior of the tree (inner bark or sapwood) is less bright than for fresh marks (Extended Data Fig. 1), conspicuousness of the visual signal is still high. Indeed, the concealing of old marks with bark strips produced the same brown bear response as manipulations on fresh marks (Extended Data Fig. 7).

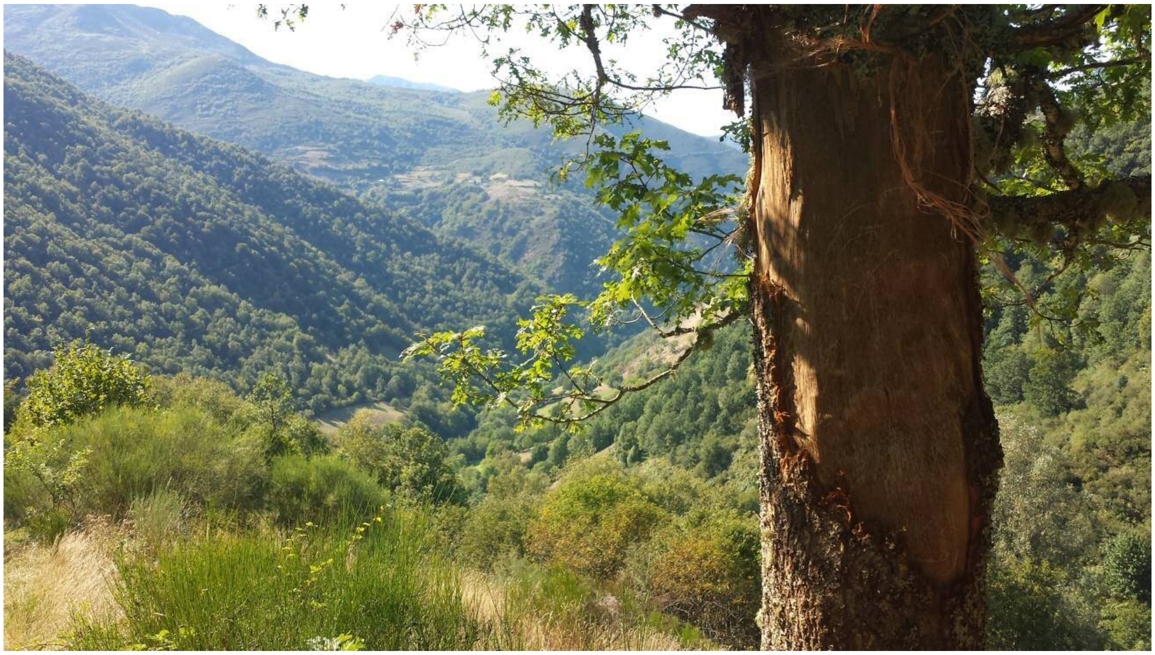

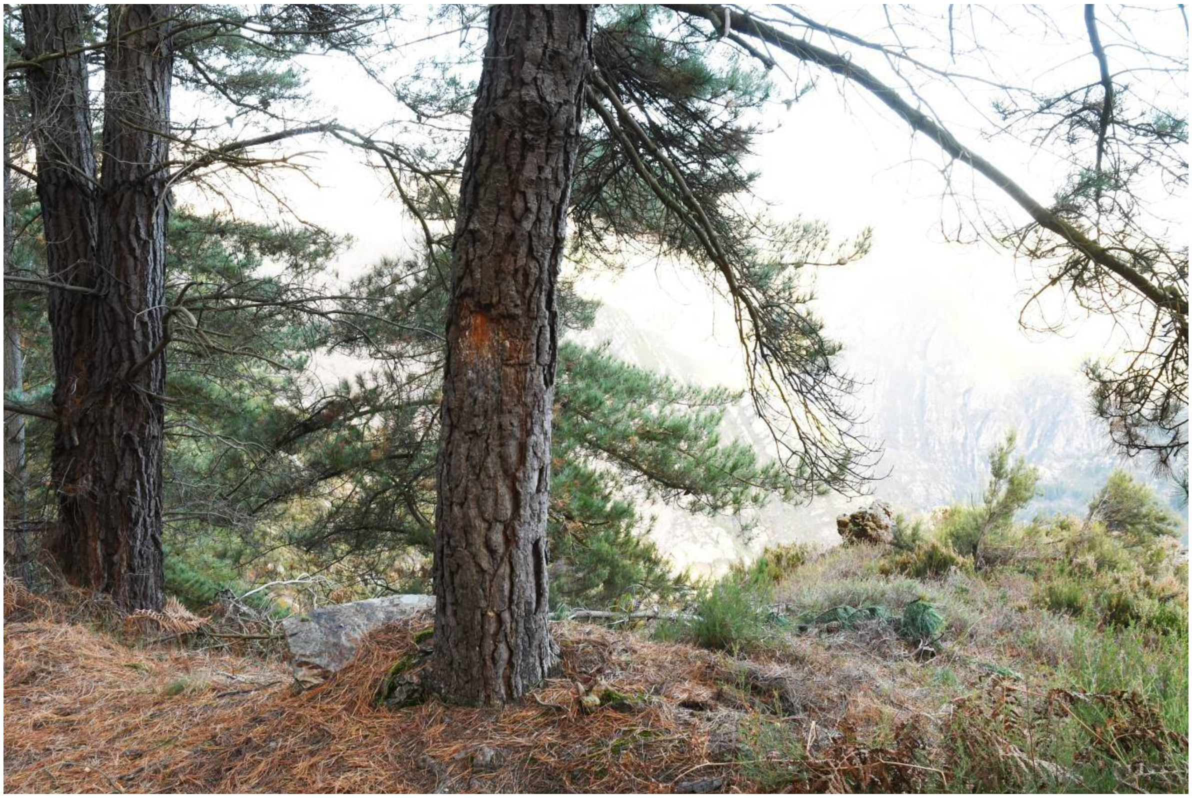

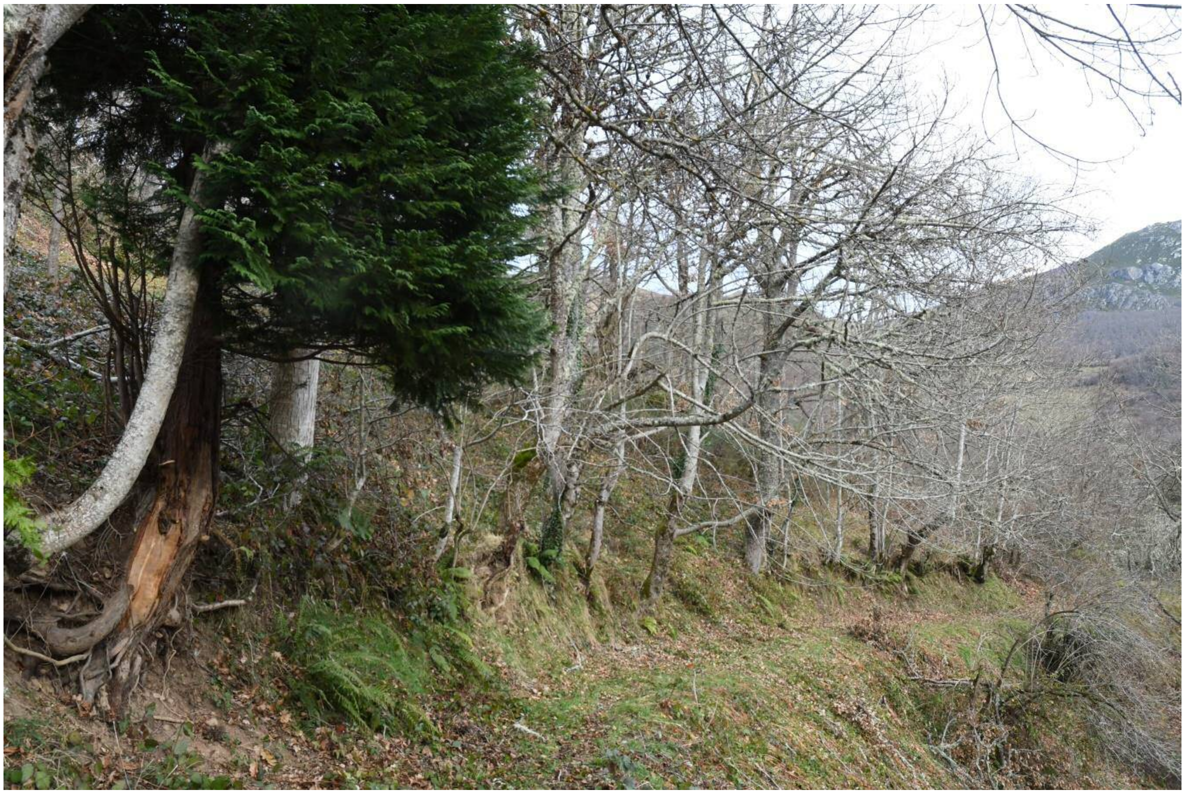

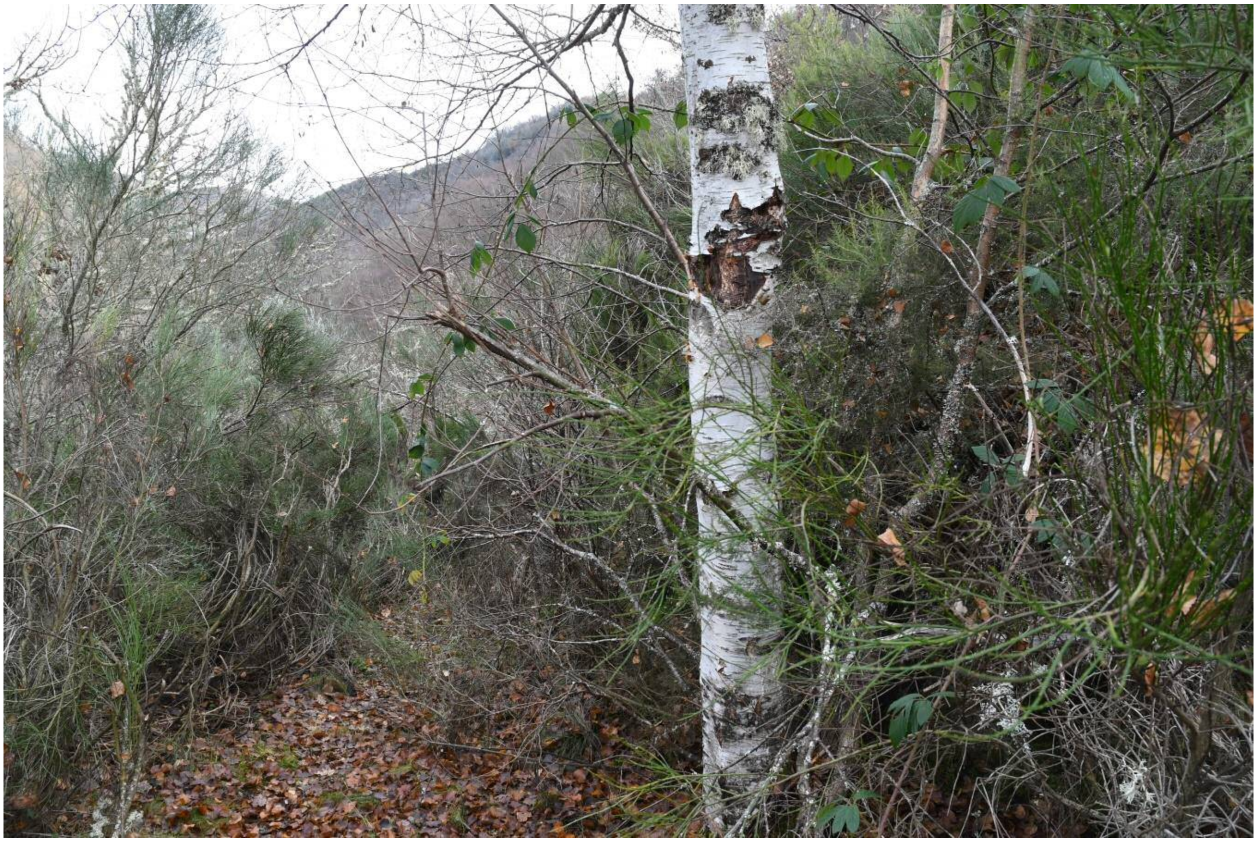

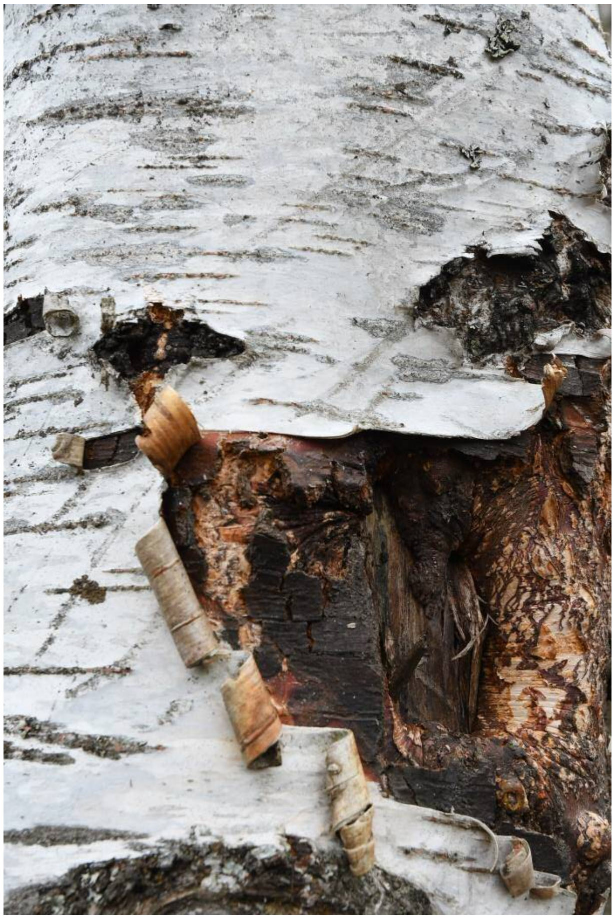
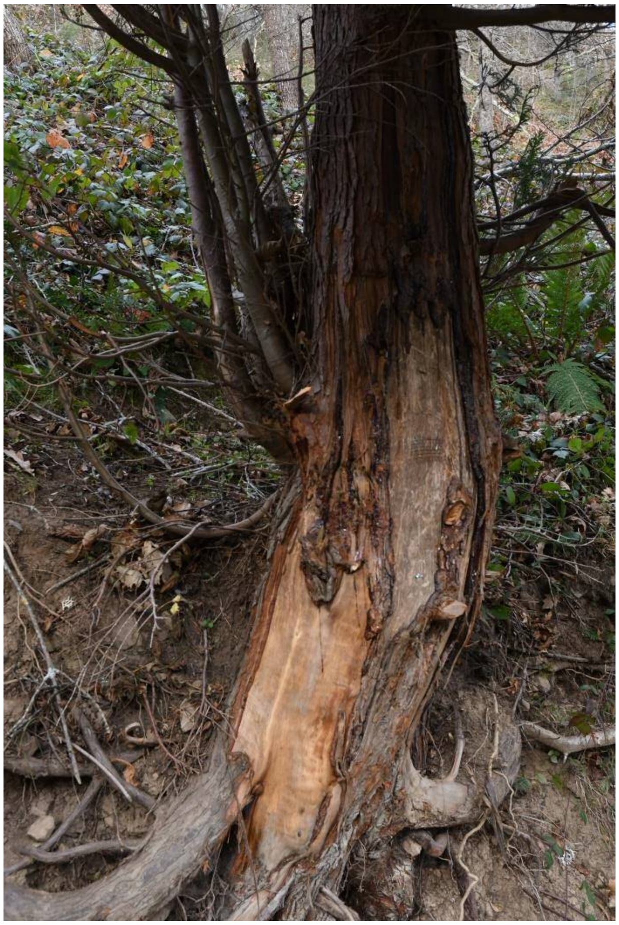

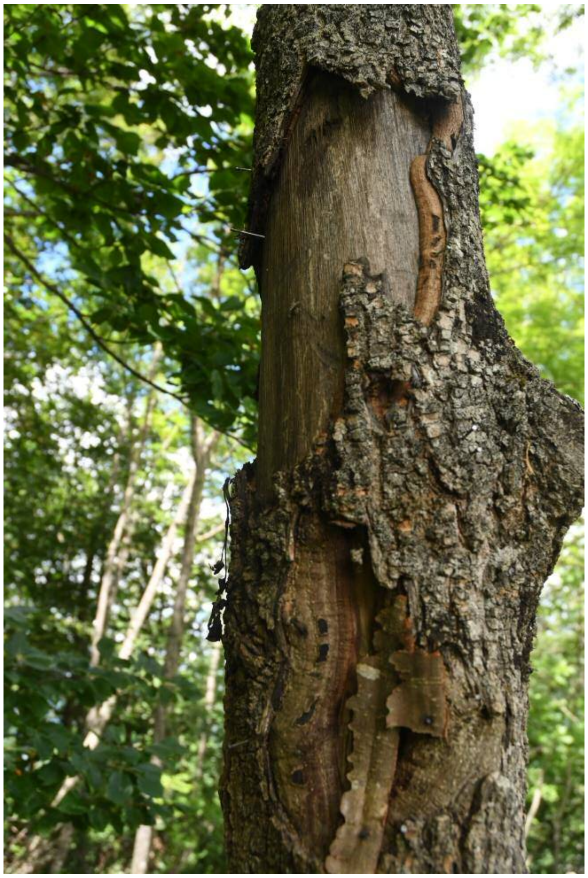
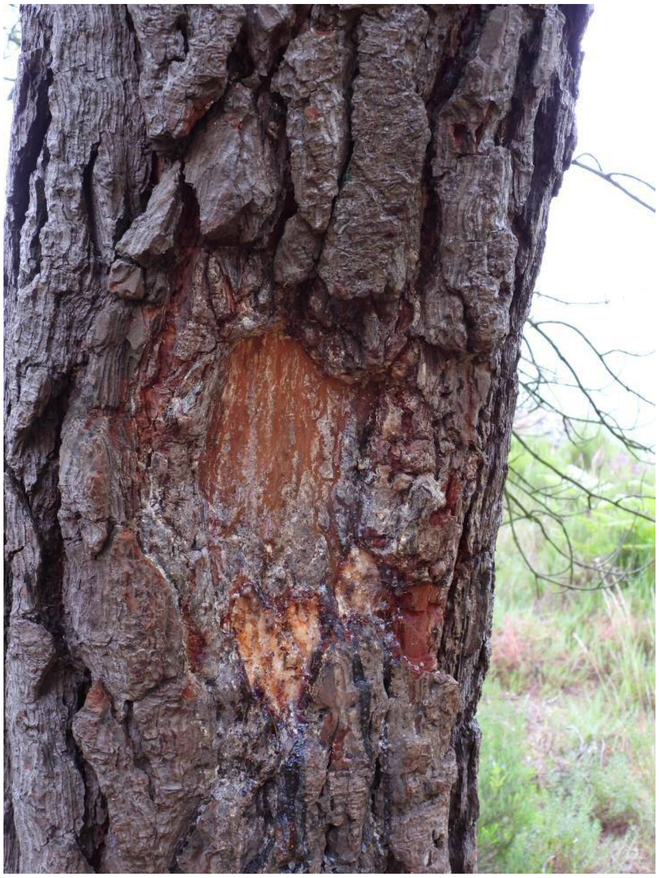
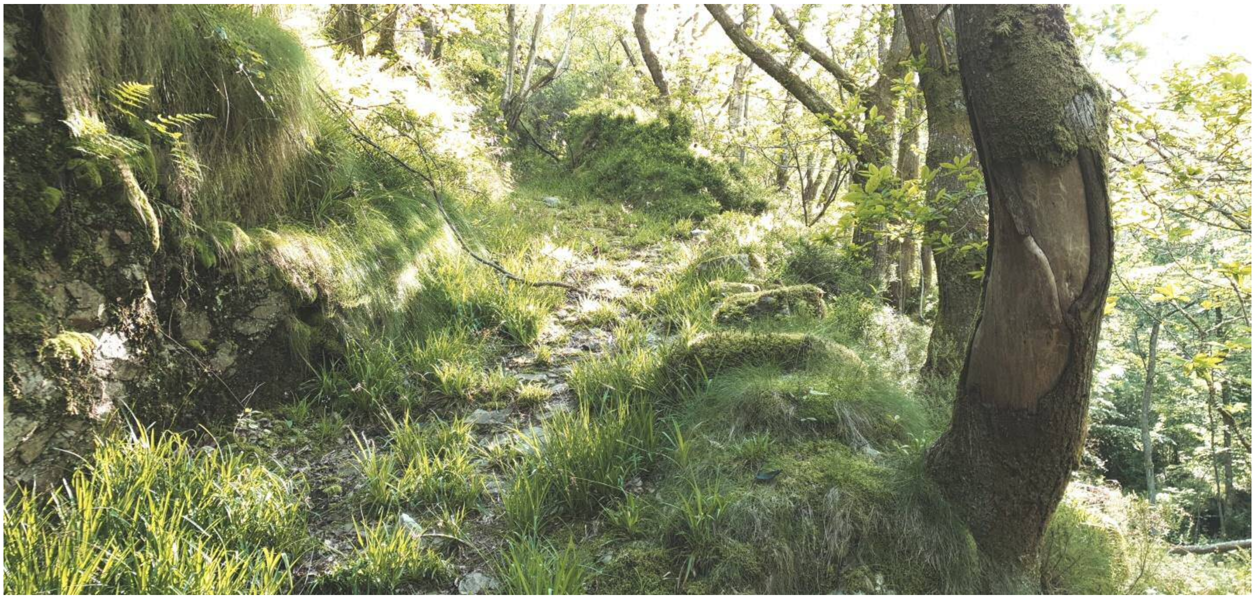

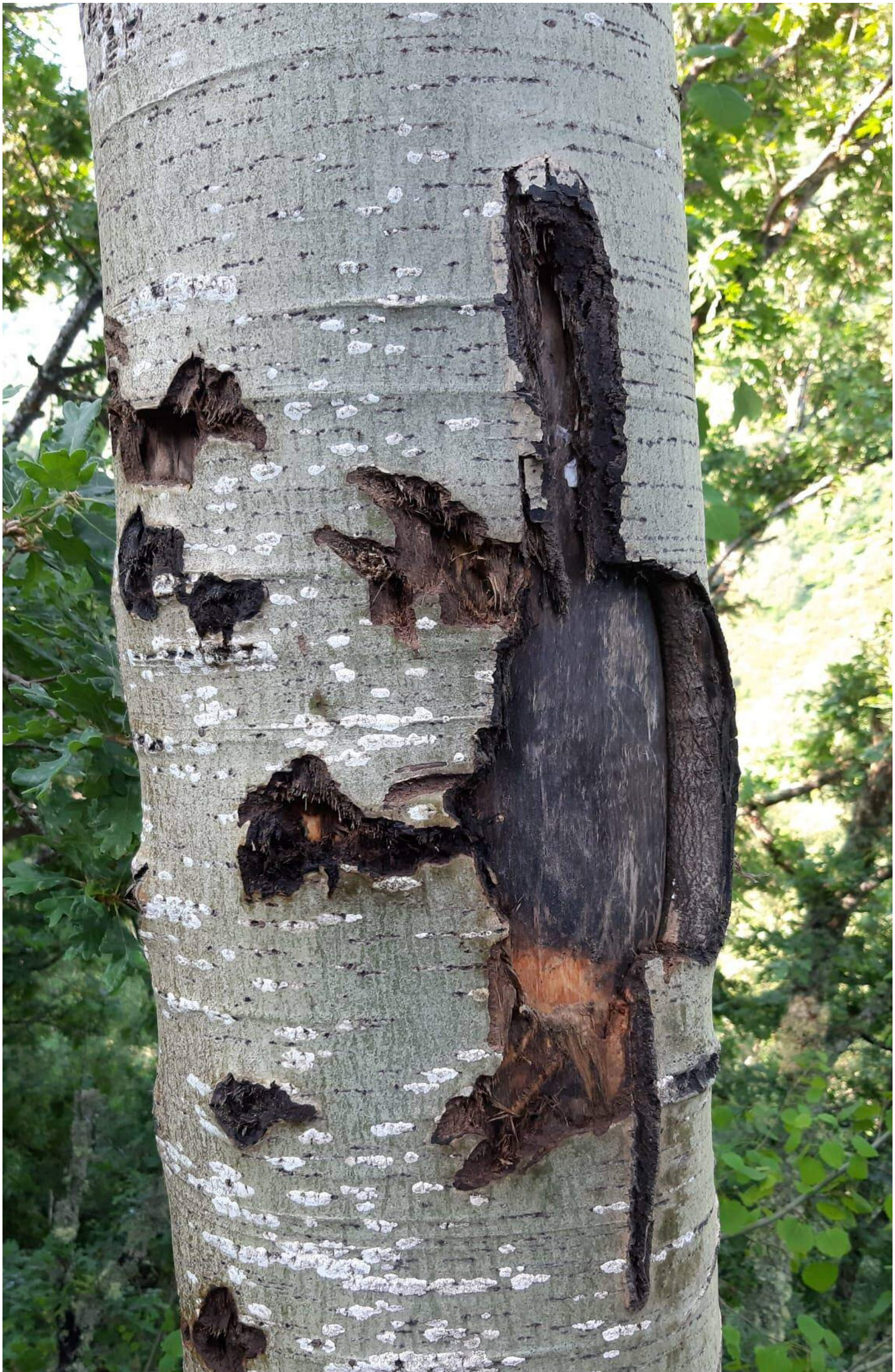

Supplement: Supplementary file 2 — Supplementary Figure 2. [file 41598_2021_88472_MOESM2_ESM.pdf]
