# Supplementary material for: Visual marking in mammals first proved by manipulations of brown bear tree debarking
Source: Sci Rep. 2021 May 4;11:9492. doi: 10.1038/s41598-021-88472-5 (PMC8096968; doi:10.1038/s41598-021-88472-5)

Visual marking in mammals first proved by manipulations of brown bear tree debarking

Vincenzo Penteriani[1*¶], Enrique González-Bernardo[1,2¶], Alfonso Hartasánchez[3], Héctor Ruiz-Villar[1], Ana Morales-González[4], Andrés Ordiz[5], Giulia Bombieri[6], Juan Díaz García[7], David Cañedo[7], Chiara Bettega[1], María del Mar Delgado[1]

1. Research Unit of Biodiversity (UMIB, CSIC-UO-PA), Mieres Campus, 33600 Mieres, Spain

2. Pyrenean Institute of Ecology (IPE), C.S.I.C., Avda. Montañana 1005, 50059 Zaragoza, Spain

3. FAPAS Fondo para la Protección de los Animales Salvajes, Ctra. AS-228, km 8,9 – Tuñón, 33115 Santo Adriano, Asturias, Spain.

4. Estación Biológica de Doñana, C.S.I.C., Department of Conservation Biology, Avda. Americo Vespucio 26, 41092 Sevilla, Spain

5. Faculty of Environmental Sciences and Natural Resource Management, Norwegian University of Life Sciences, Postbox 5003, NO-1432, Ås, Norway

6. MUSE - Museo delle Scienze, Sezione Zoologia dei Vertebrati, Corso del Lavoro e della Scienza 3, I-38123, Trento, Italy.

7. Consejería de Ordenación del Territorio, Infraestructuras y Medio Ambiente, Dirección General de Biodiversidad, Principado de Asturias, Oviedo, Spain

*Correspondence author: v.penteriani@csic.es

[¶] These authors contributed equally to this work

Extended Data Fig. 3 | Visual signalling in brown bears (colour and black and white photos). 1. On the 29th of April 2019 the tree had not yet been used for visual signalling by brown bears, even though it was a well-known rubbing tree. 2-6. On the 7th of May 2019 an adult male scratched and bit the trunk to remove the outer bark and, thus, revealed the bright and conspicuous layer of inner bark. 7. An example of an adult male brown bear leaving a visual mark (light grey patch) on a tree. The bear is scratching the trunk with his left paw, first removing a rectangular strip of bark. 8. Always with the left paw, the bear increases the size of the mark. 9-11. By rising on its hind legs, the bear removes a new piece of bark with its mouth, resulting in the bark being completely separated from the trunk when the bear drops down.

1.

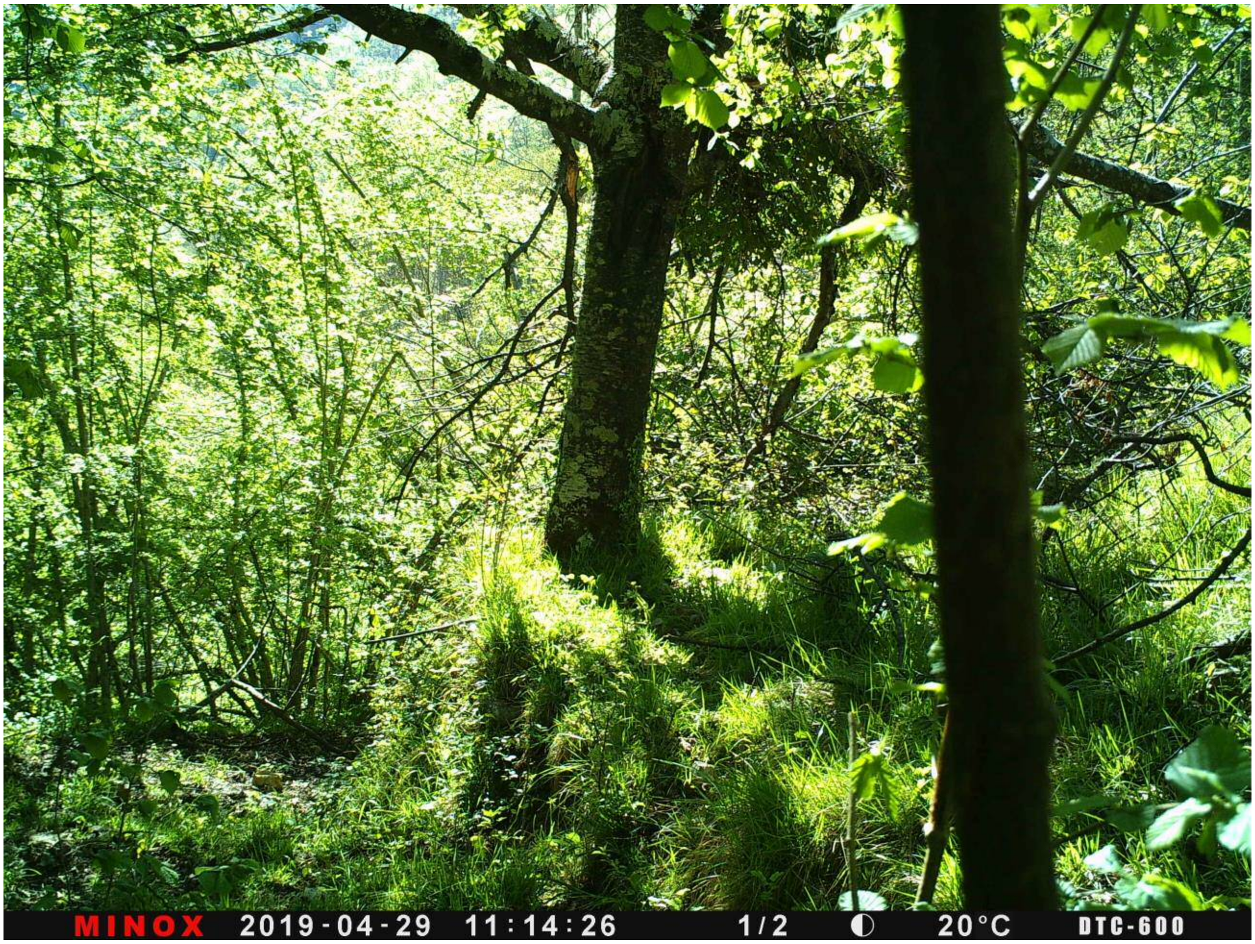

2.

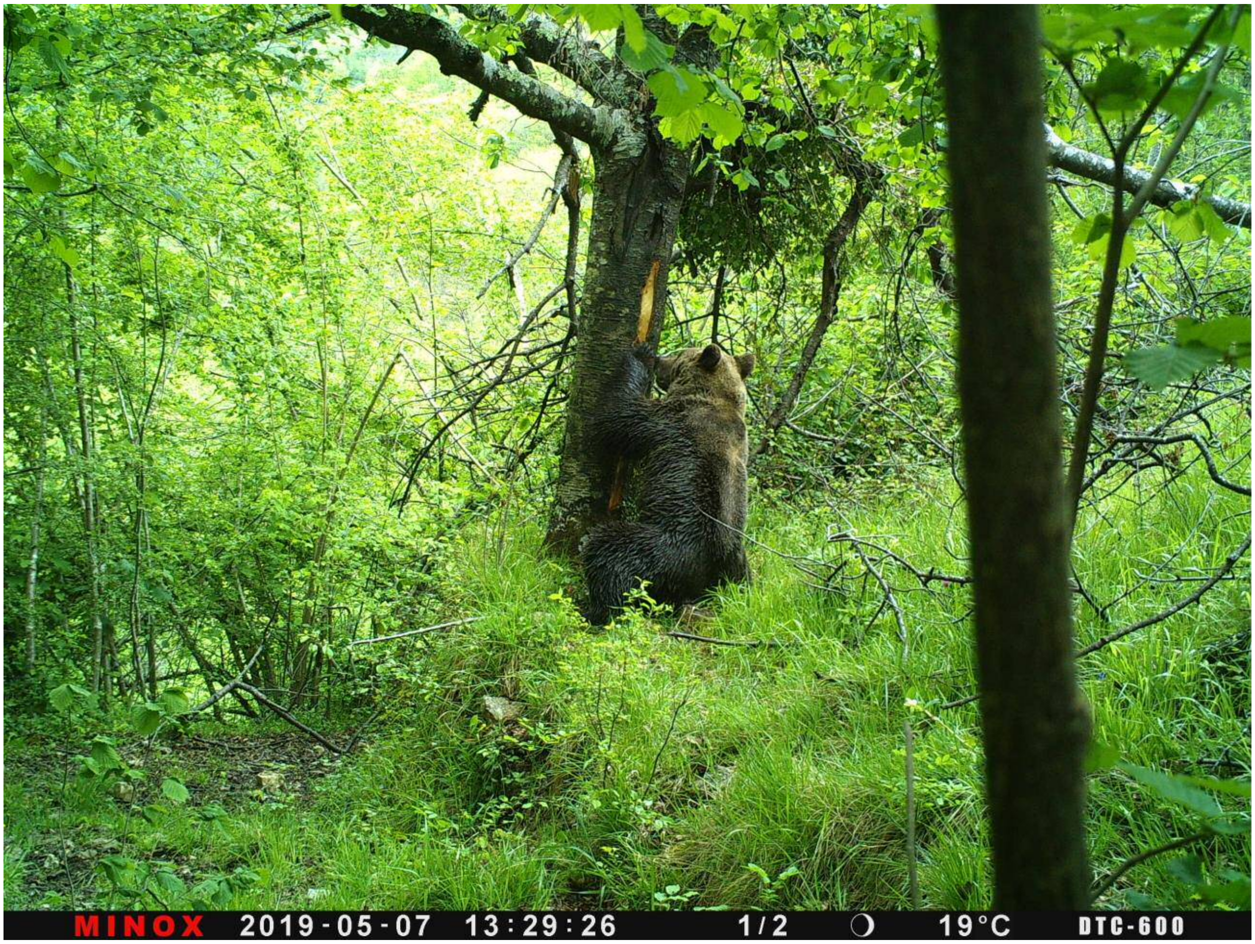

3.

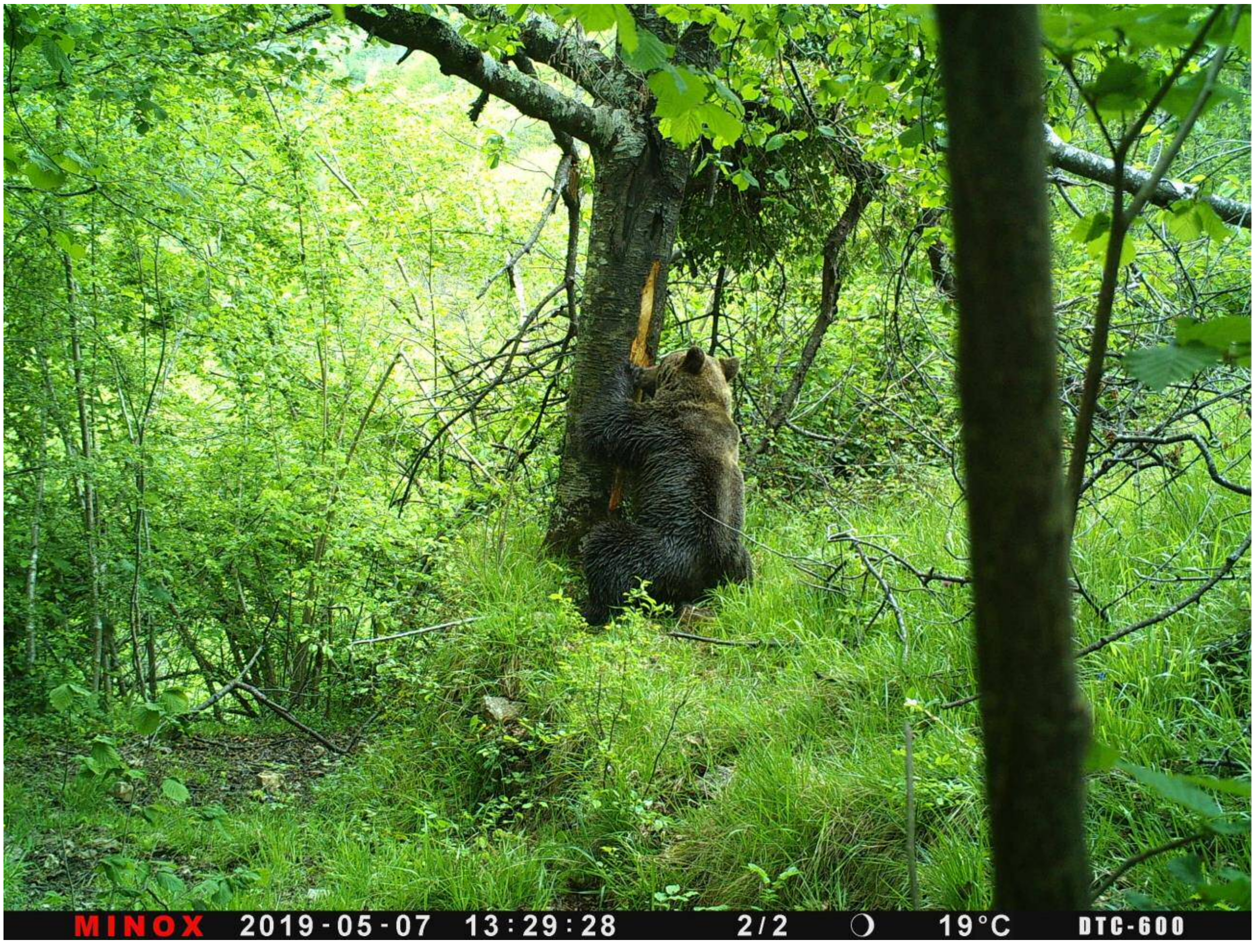

4.

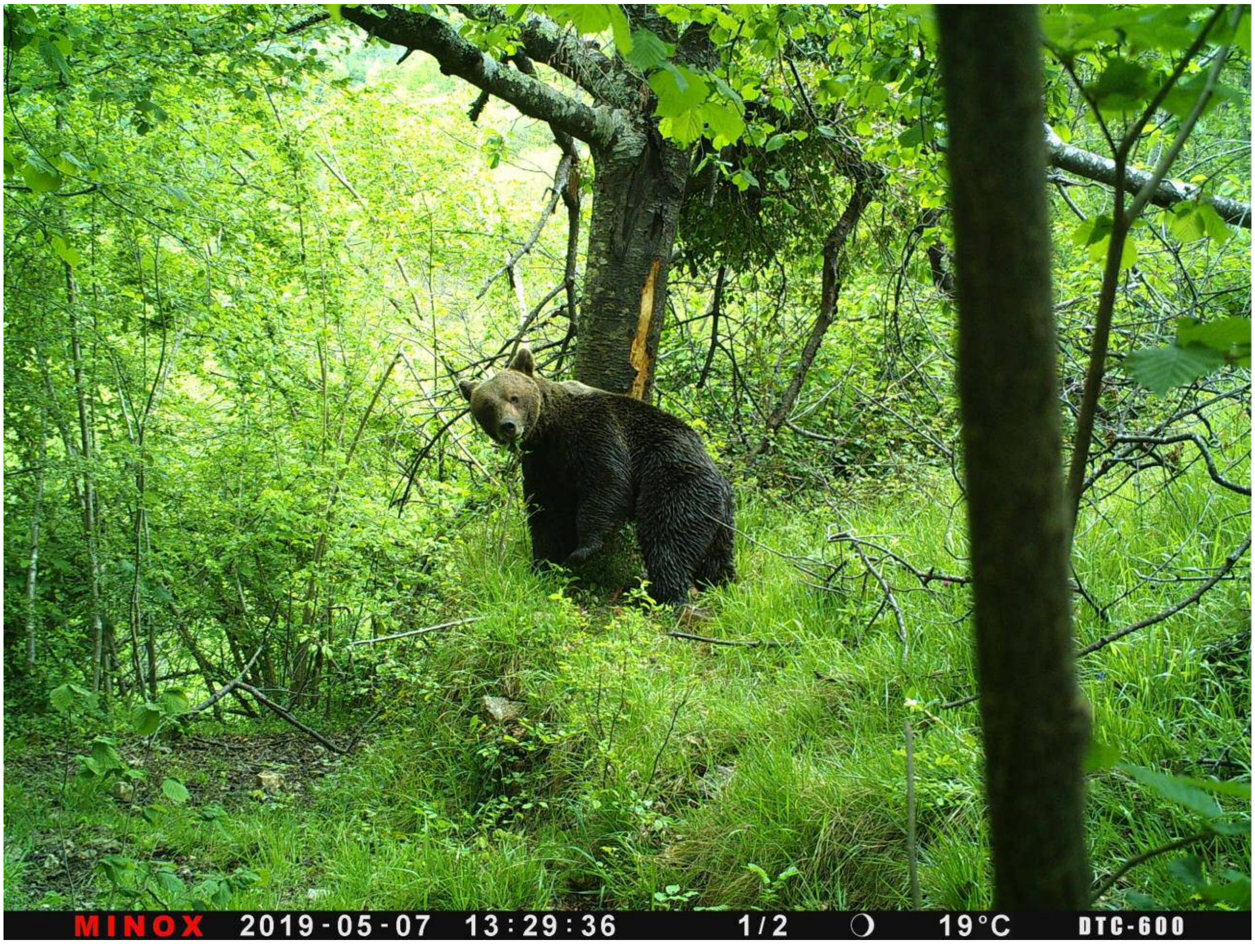

5.

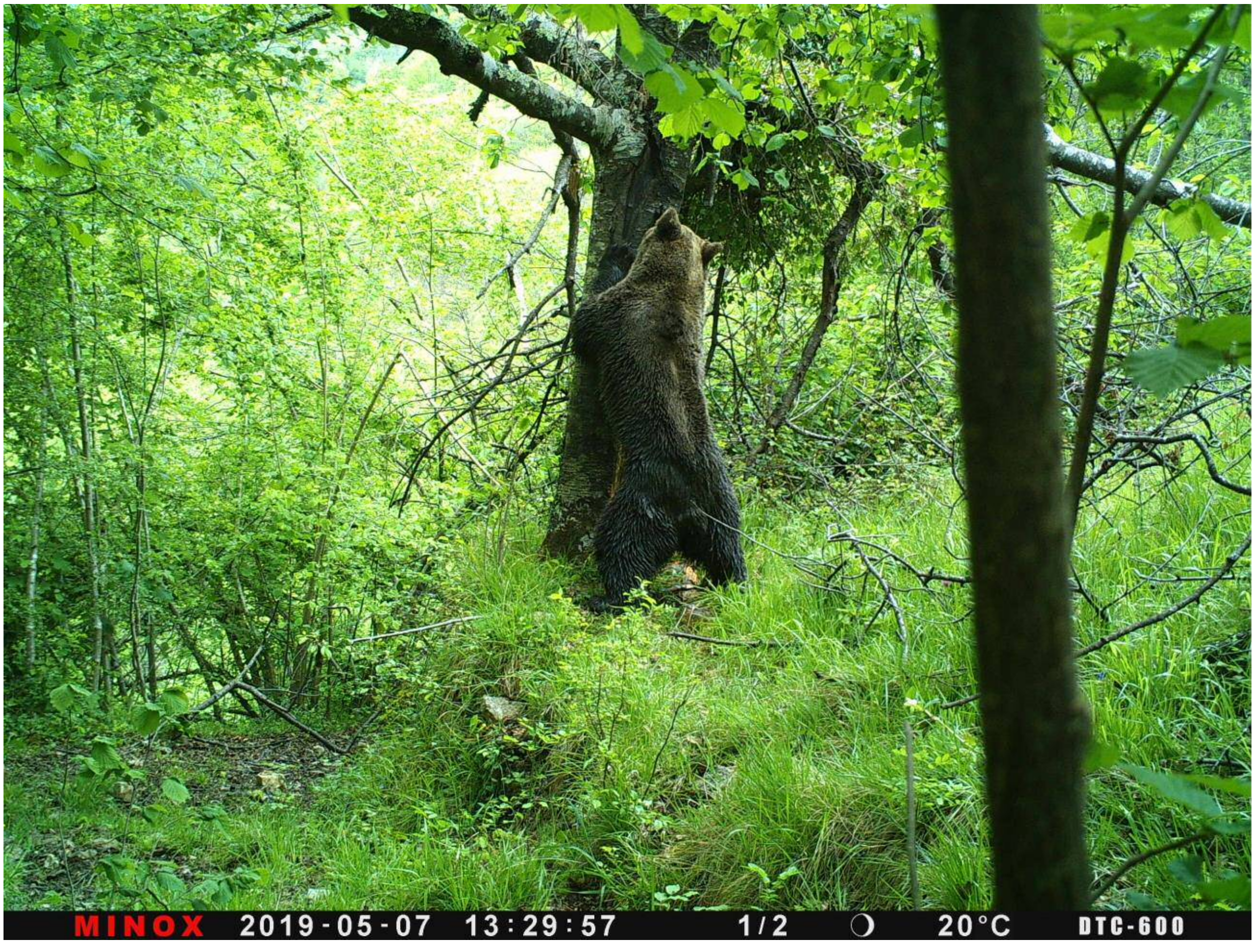

6.

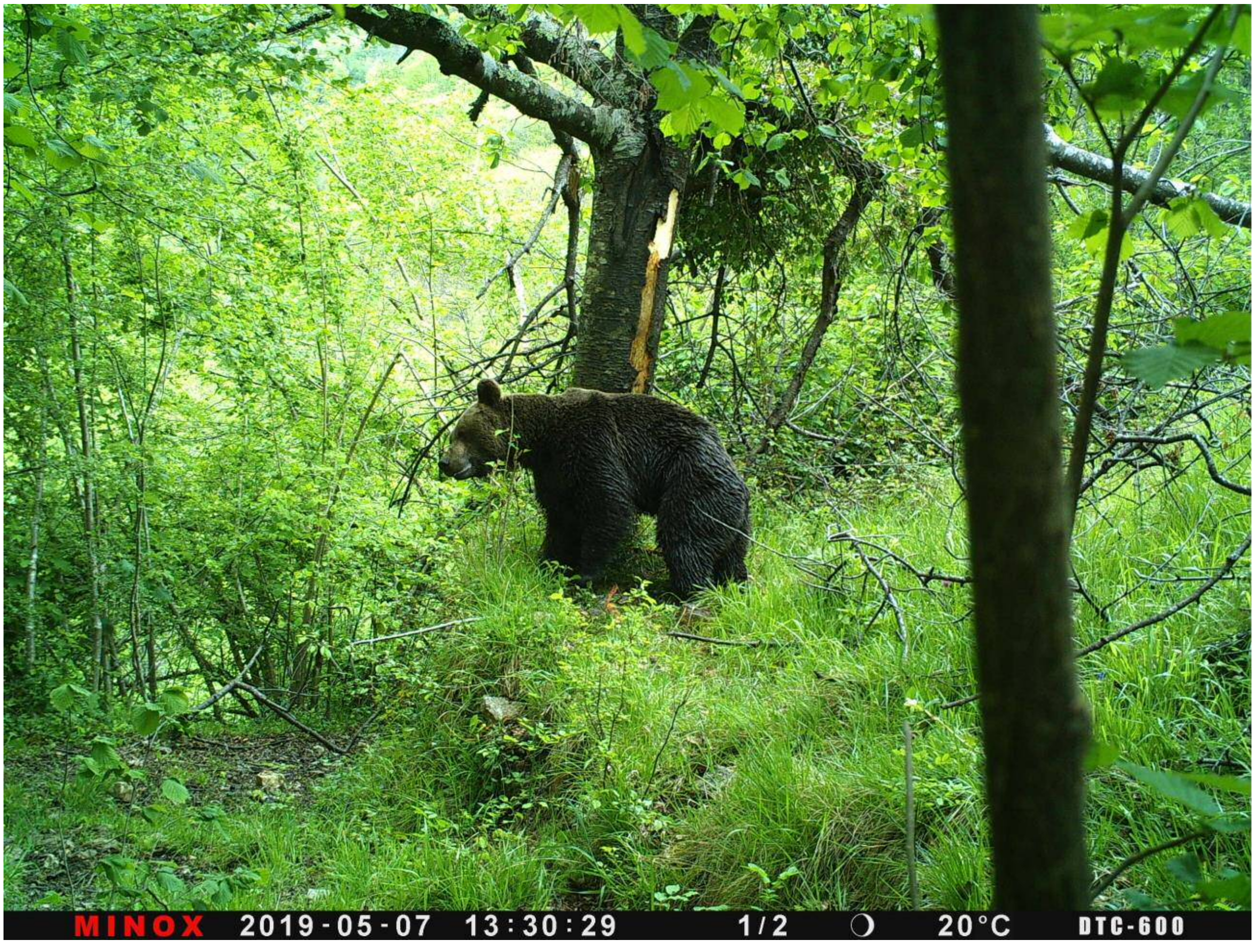

7.

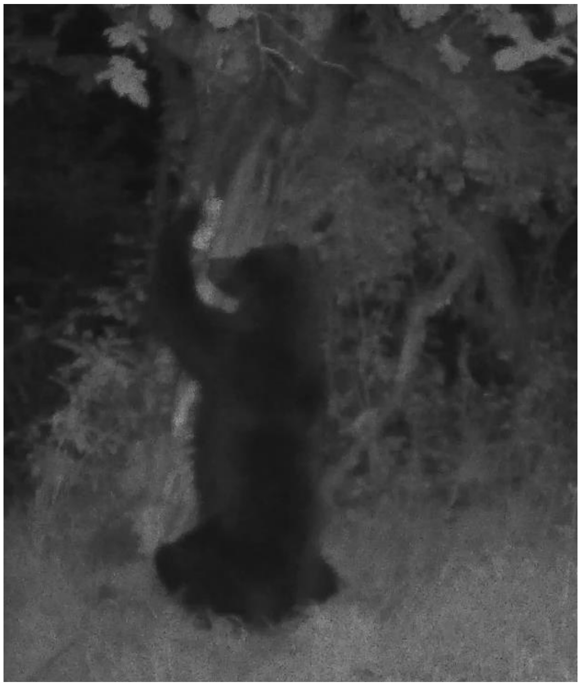

8.

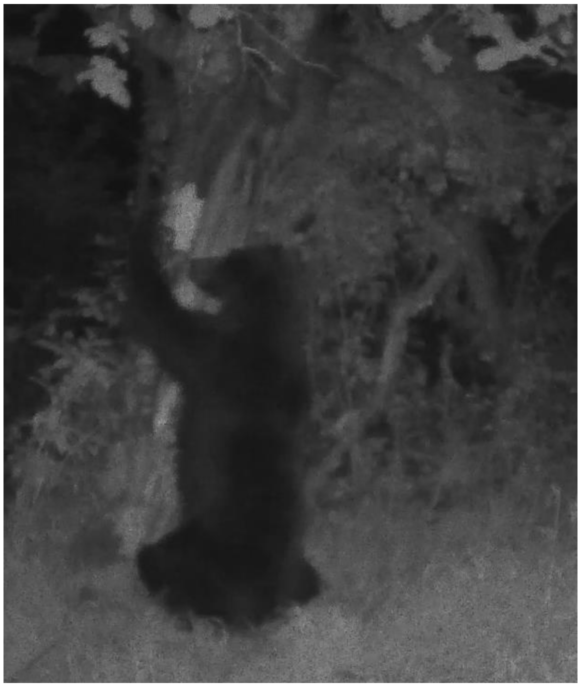

9.

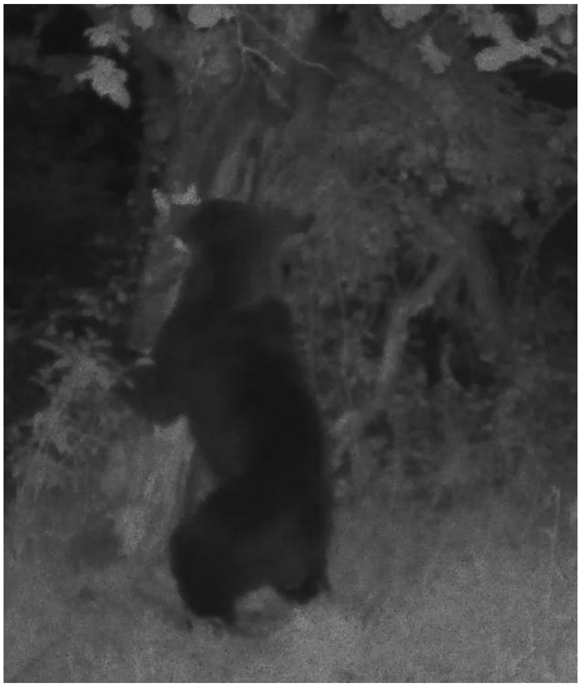

10.

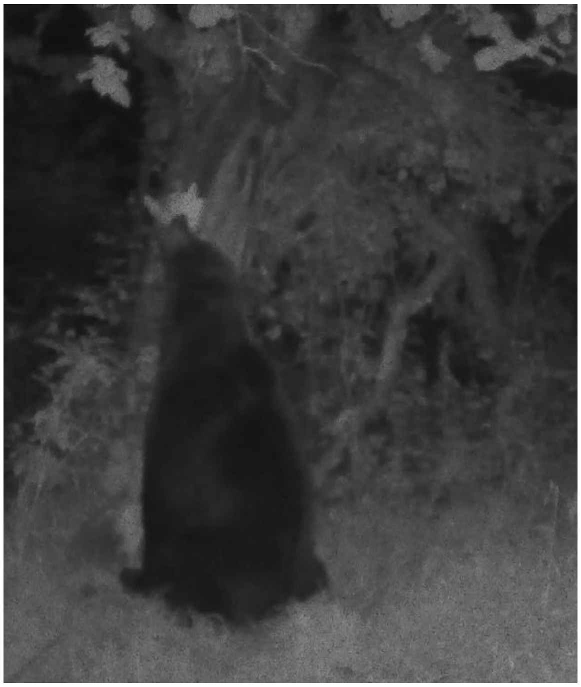

11.

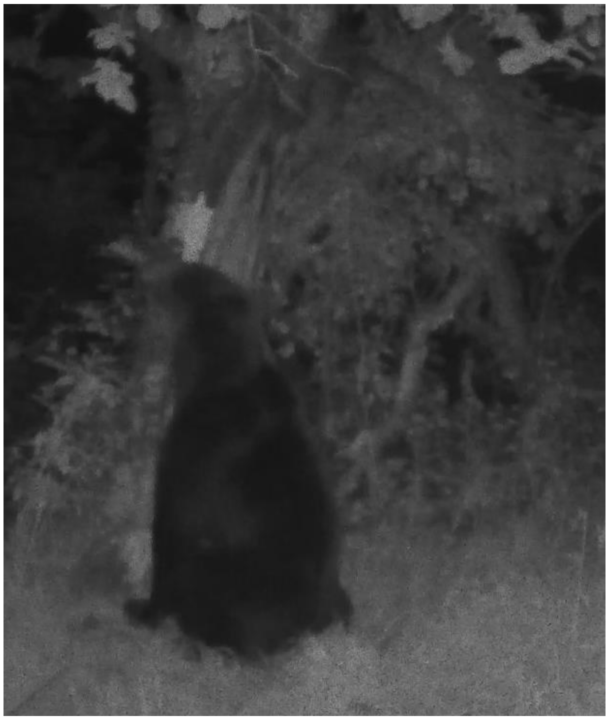

Supplement: Supplementary file 3 — Supplementary Figure 3. [file 41598_2021_88472_MOESM3_ESM.pdf]
