# Supplementary material for: Visual marking in mammals first proved by manipulations of brown bear tree debarking
Source: Sci Rep. 2021 May 4;11:9492. doi: 10.1038/s41598-021-88472-5 (PMC8096968; doi:10.1038/s41598-021-88472-5)

Visual marking in mammals first proved by manipulations of brown bear tree debarking

Vincenzo Penteriani[1*¶], Enrique González-Bernardo[1,2¶], Alfonso Hartasánchez[3], Héctor Ruiz-Villar[1], Ana Morales-González[4], Andrés Ordiz[5], Giulia Bombieri[6], Juan Díaz García[7], David Cañedo[7], Chiara Bettega[1], María del Mar Delgado[1]

1. Research Unit of Biodiversity (UMIB, CSIC-UO-PA), Mieres Campus, 33600 Mieres, Spain

2. Pyrenean Institute of Ecology (IPE), C.S.I.C., Avda. Montañana 1005, 50059 Zaragoza, Spain

3. FAPAS Fondo para la Protección de los Animales Salvajes, Ctra. AS-228, km 8,9 – Tuñón, 33115 Santo Adriano, Asturias, Spain.

4. Estación Biológica de Doñana, C.S.I.C., Department of Conservation Biology, Avda. Americo Vespucio 26, 41092 Sevilla, Spain

5. Faculty of Environmental Sciences and Natural Resource Management, Norwegian University of Life Sciences, Postbox 5003, NO-1432, Ås, Norway

6. MUSE - Museo delle Scienze, Sezione Zoologia dei Vertebrati, Corso del Lavoro e della Scienza 3, I-38123, Trento, Italy.

7. Consejería de Ordenación del Territorio, Infraestructuras y Medio Ambiente, Dirección General de Biodiversidad, Principado de Asturias, Oviedo, Spain

*Correspondence author: v.penteriani@csic.es

[¶] These authors contributed equally to this work

Extended Data Fig. 6 | Example of the manipulation of bear marks by covering them with bark.

Several examples of brown bear visual mark manipulations are shown, where the mark on the trunk has been covered by strips of bark from the same tree species (1–16). 17a–17b: a marked tree (old mark) before and after manipulation (with detail of the manipulation in 17c). This is the tree where the manipulation of the mark has been removed in the Extended Data Figure 7. Details of before and after the manipulation of brown bear marks are also shown in paired photos 18a–18b, 19a–19b, 20a–20b (with detail of the manipulation in 20c). 21a–21d: an example of a removed manipulation (21a) and the remains of manipulated strips of bark at the base of the tree (pedal marking is also visible in 21d).

1.

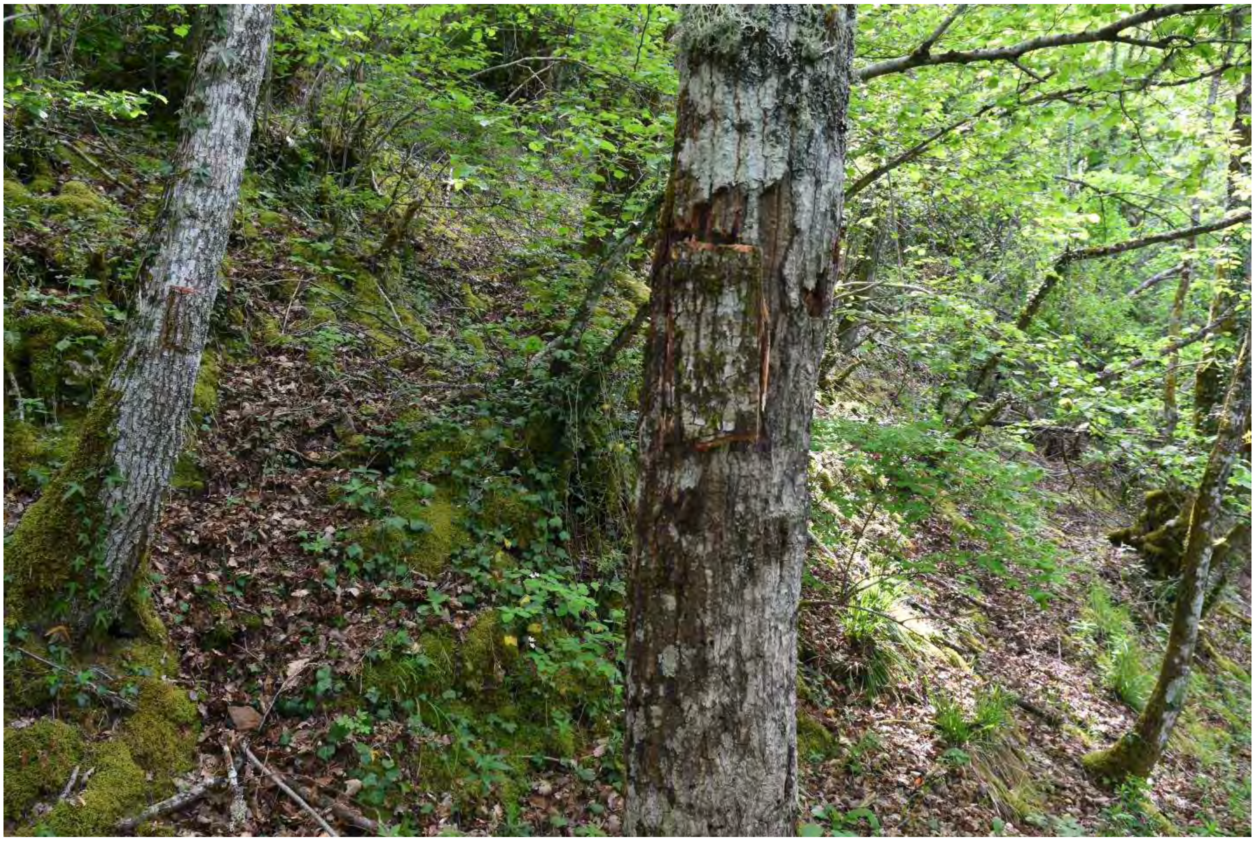

2.

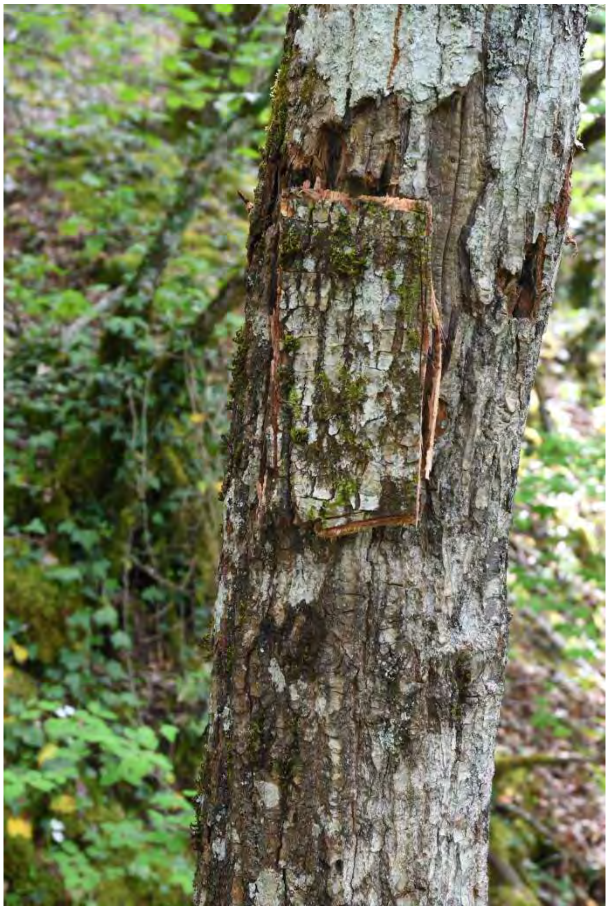

3.

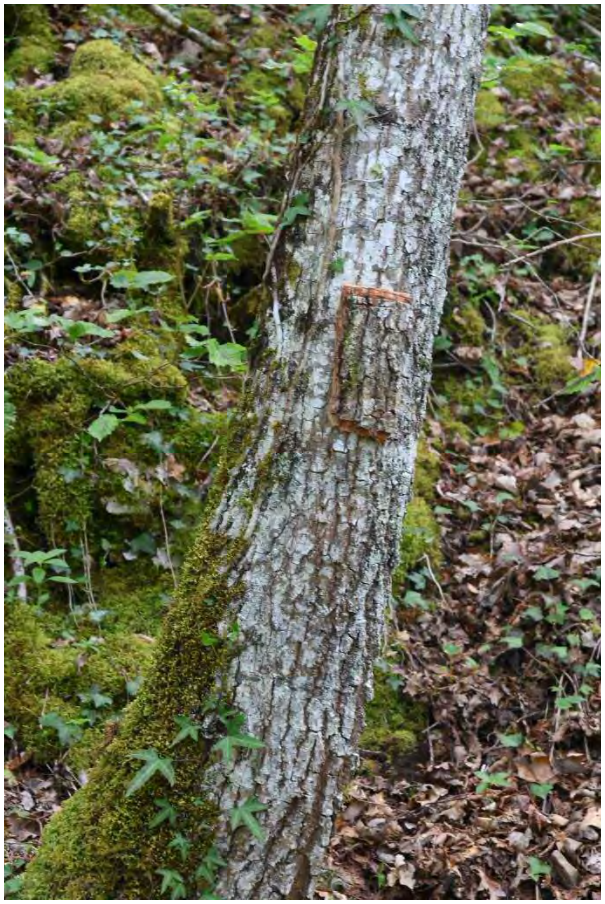

4.

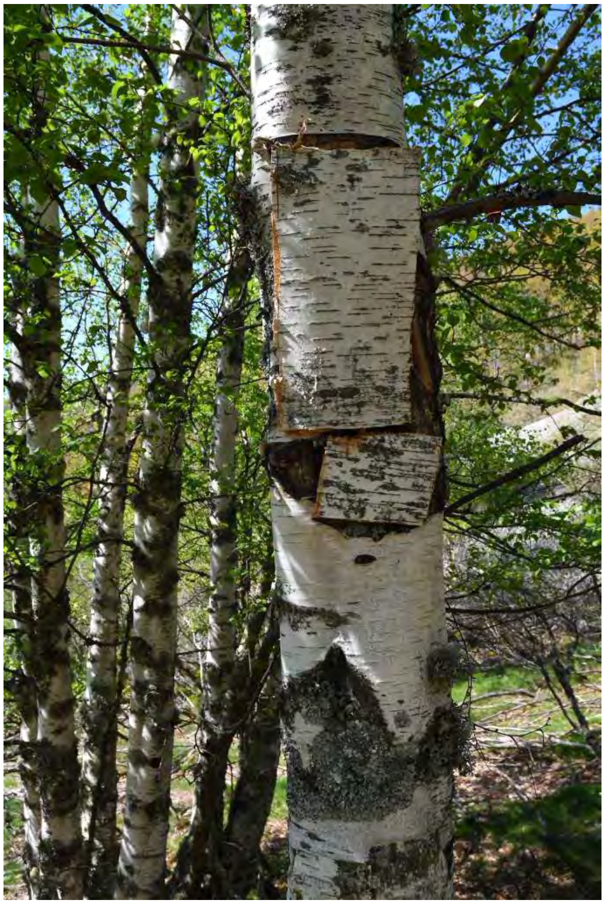

5.

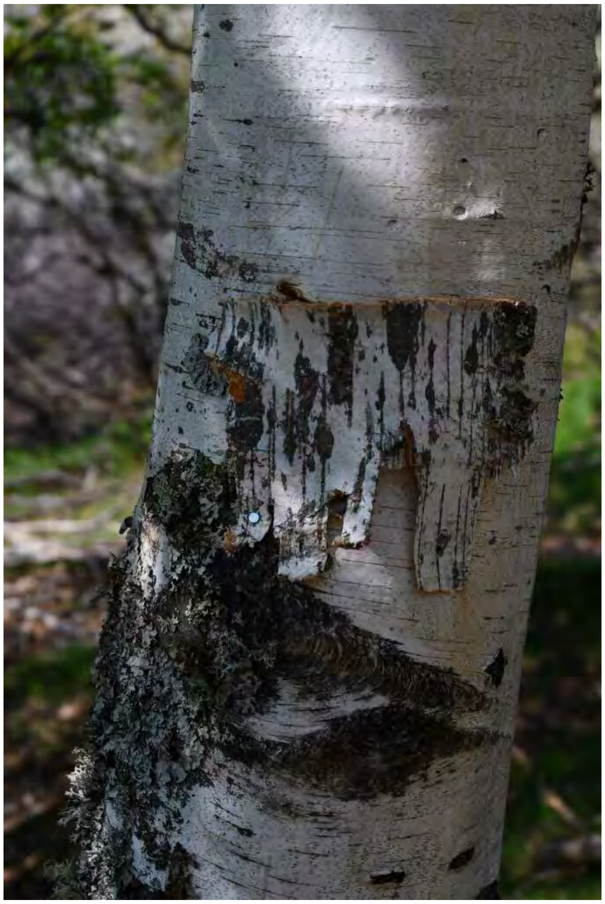

6.

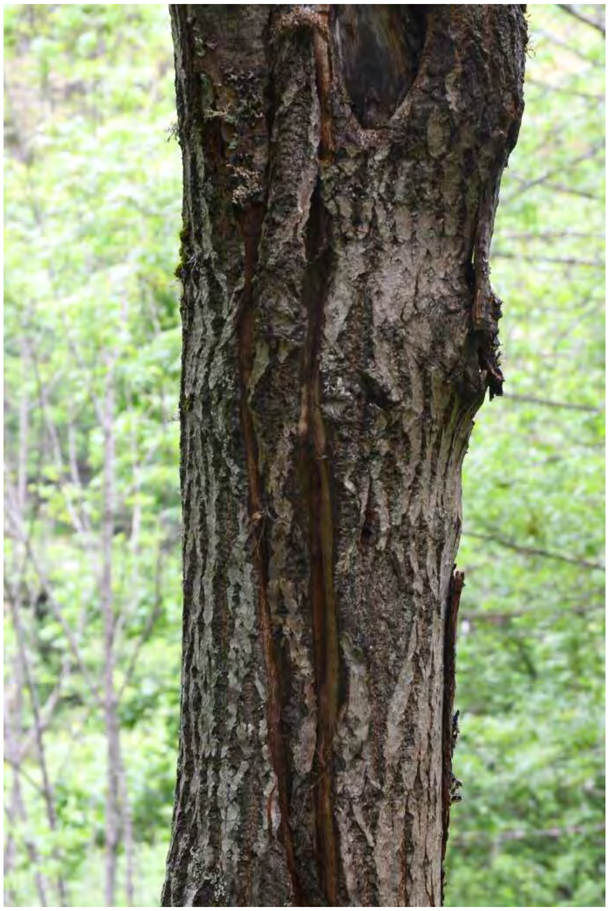

7.

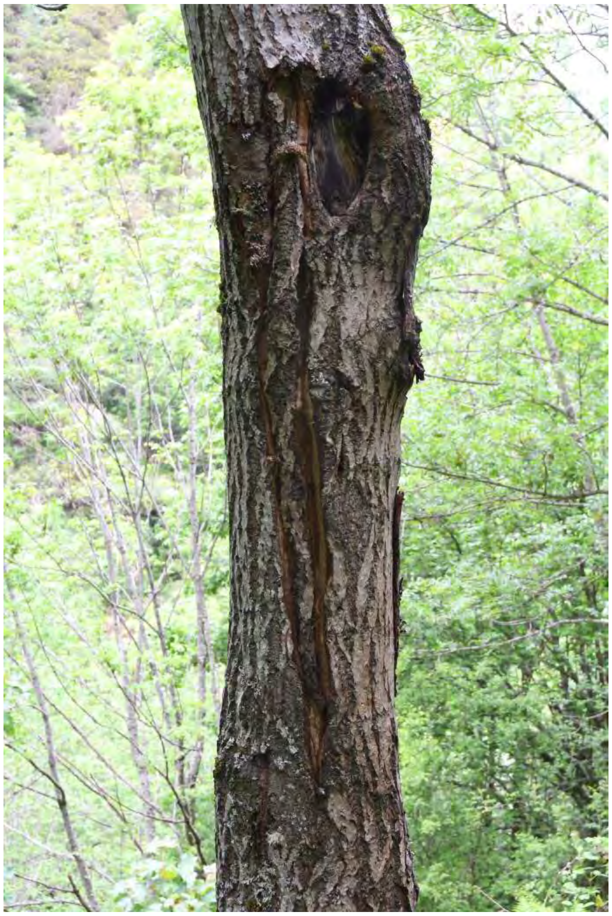

8.

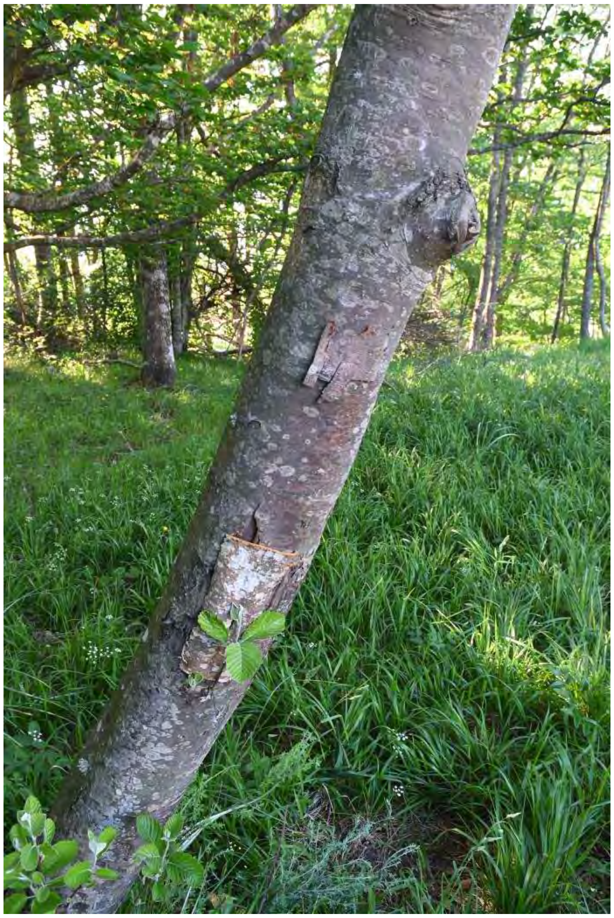

9.

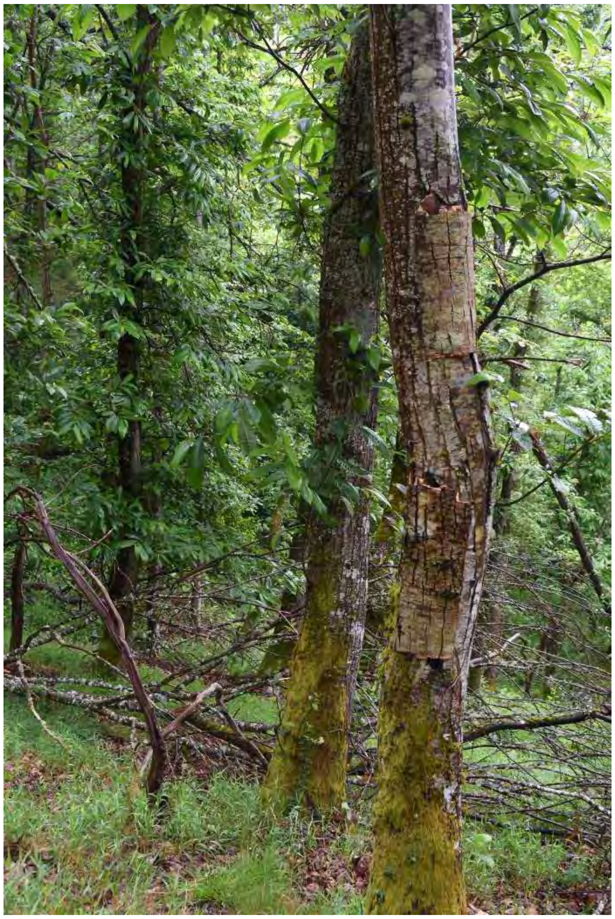

10.

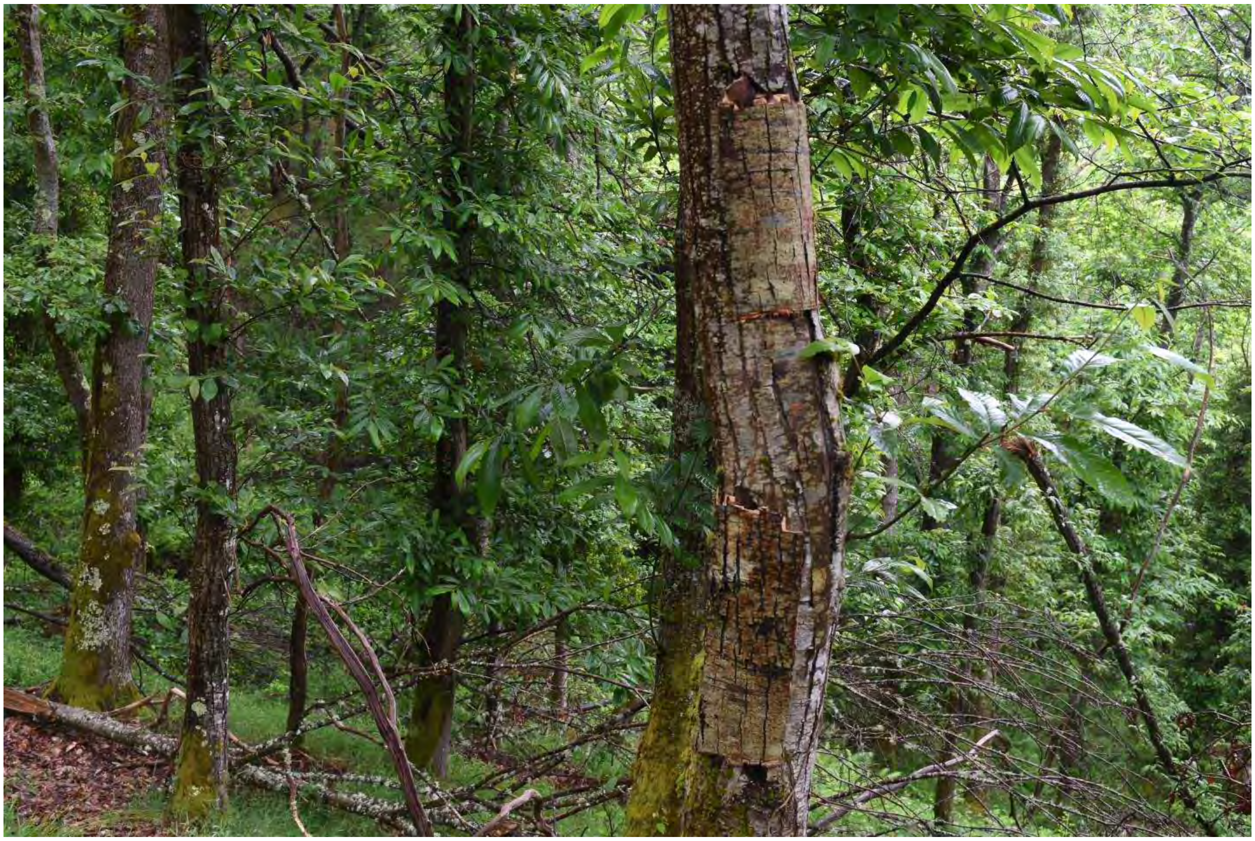

11.

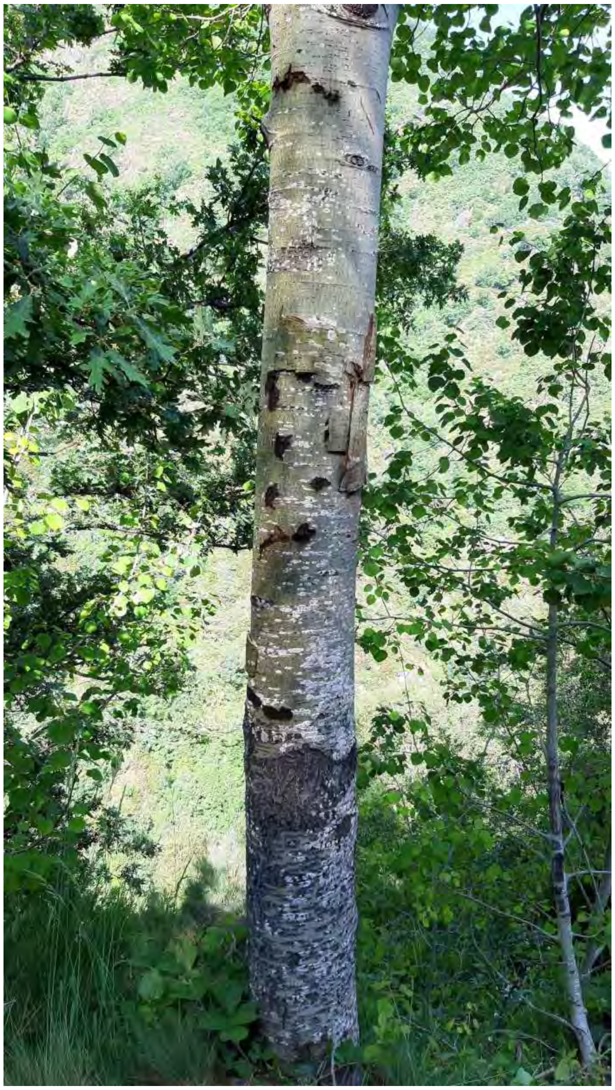

12.

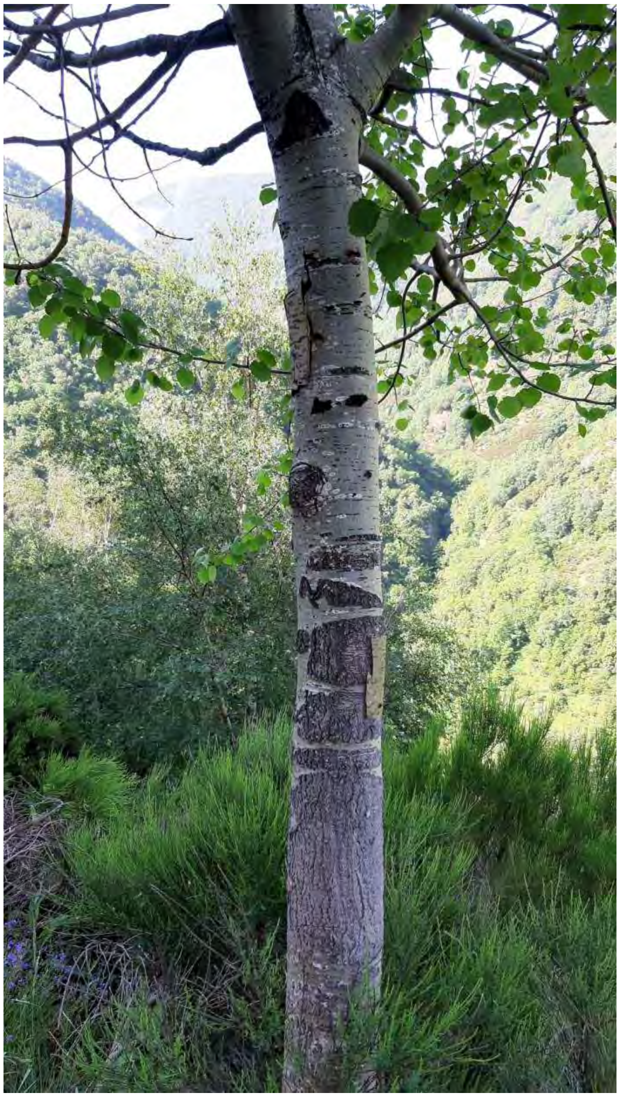

13.

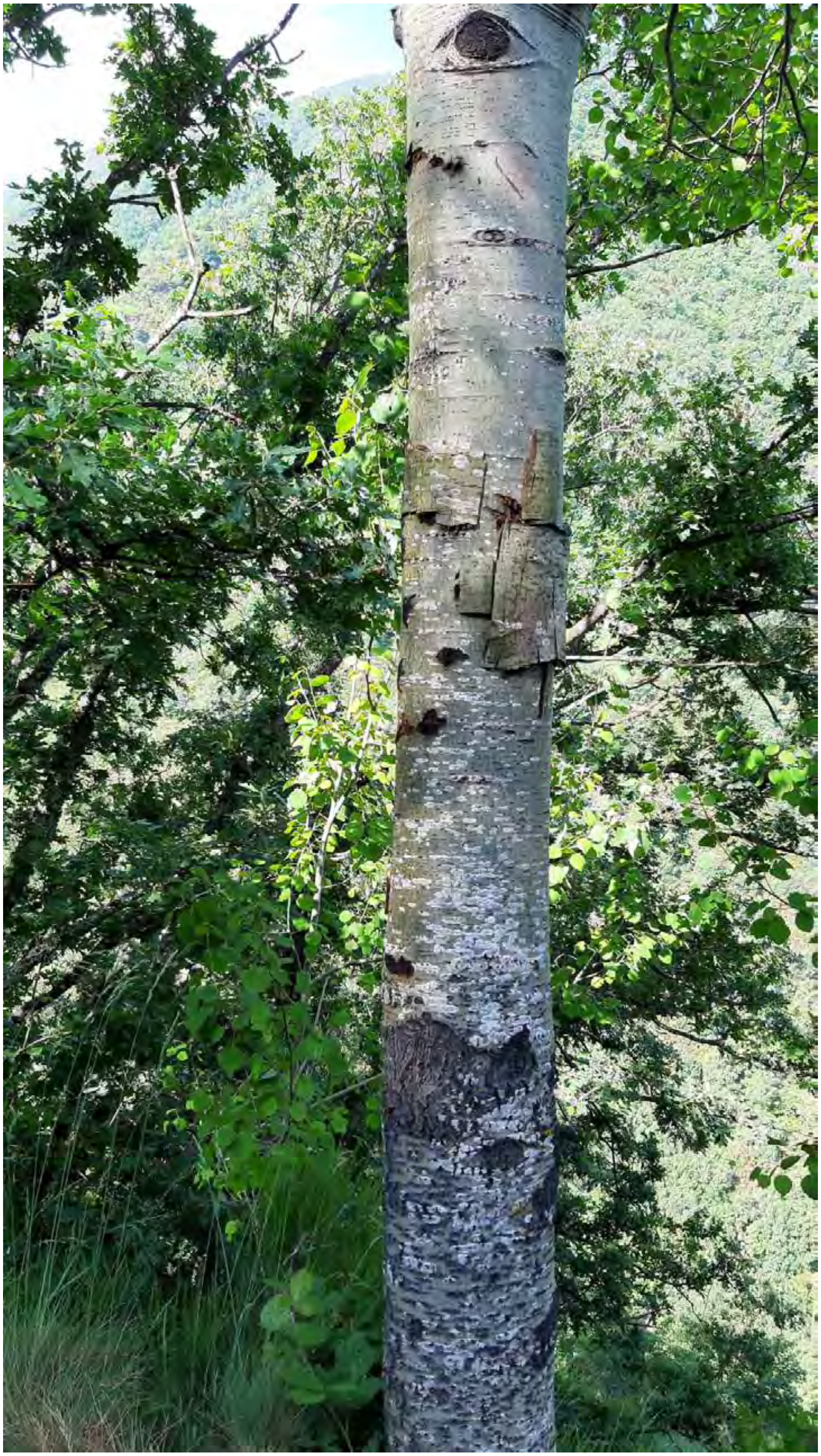

14.

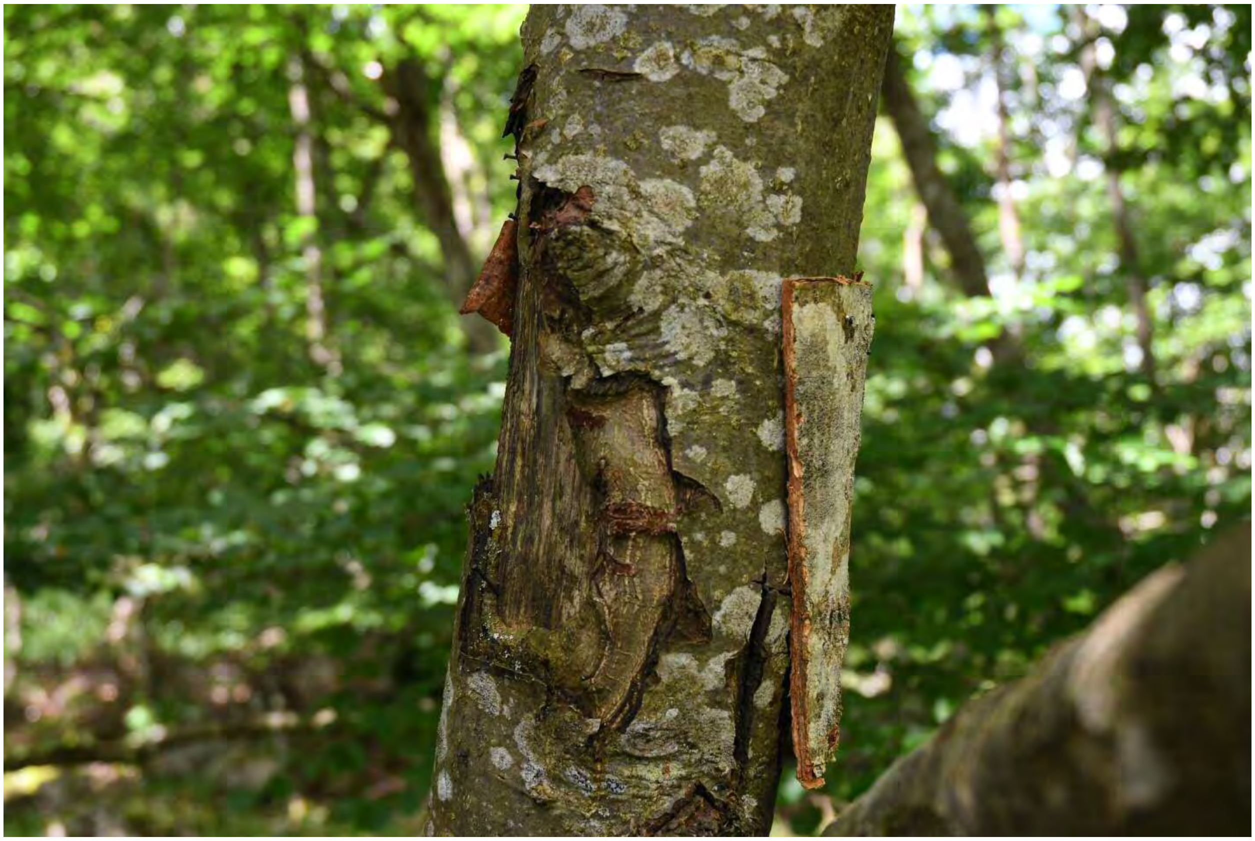

15.

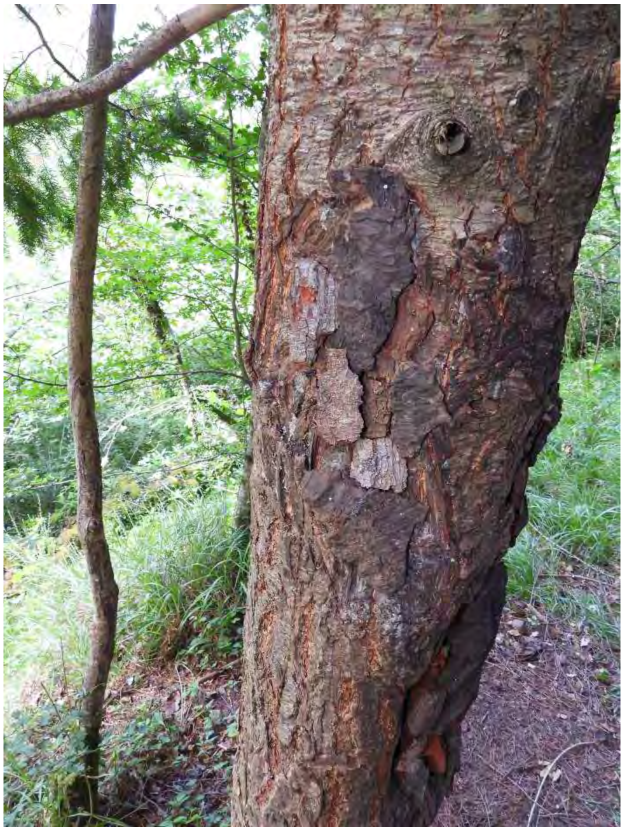

16.

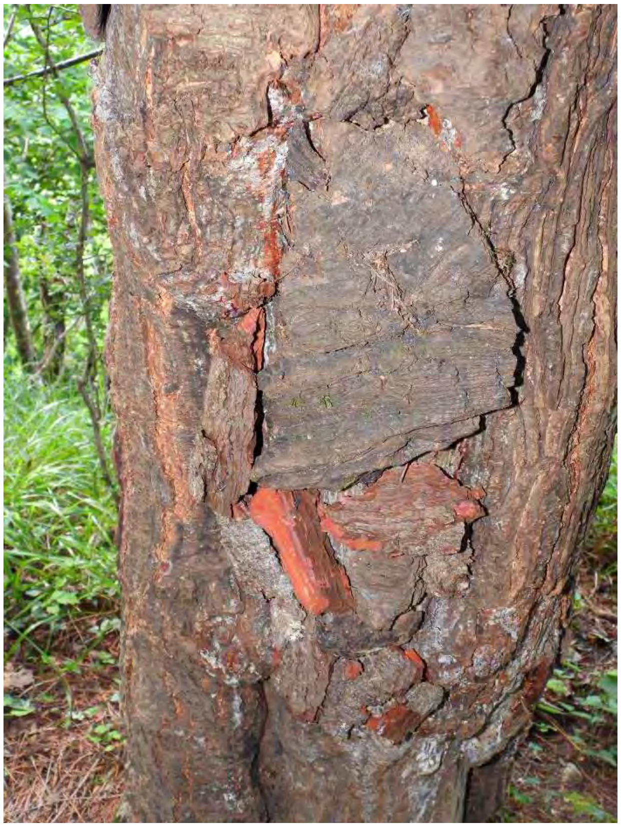

17a.

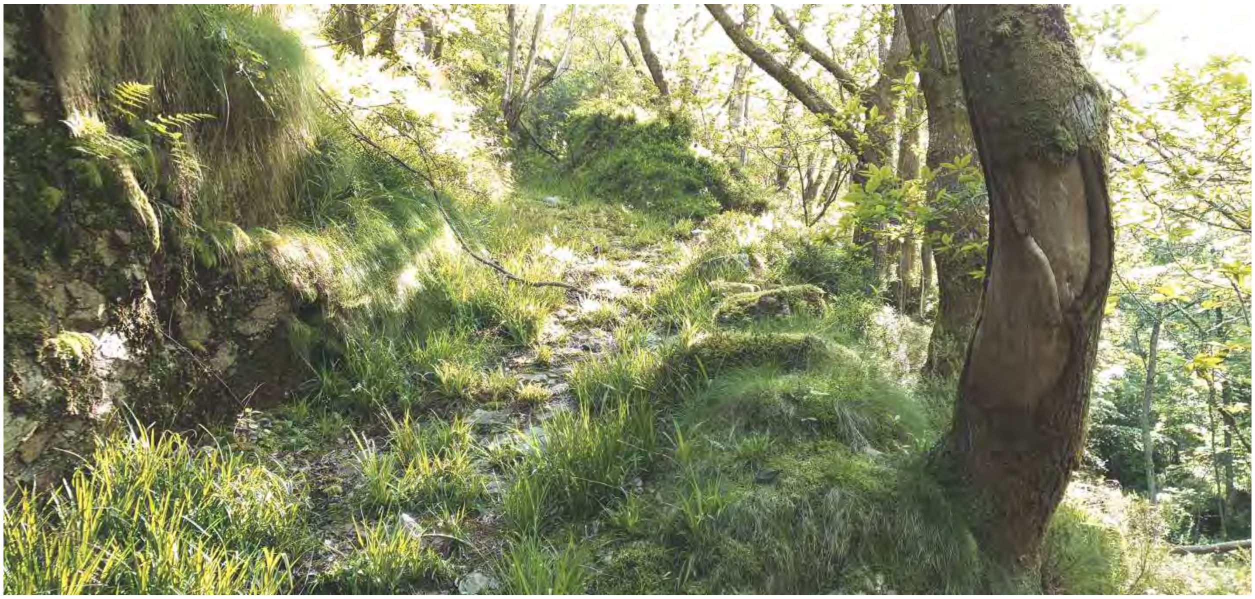

17b.

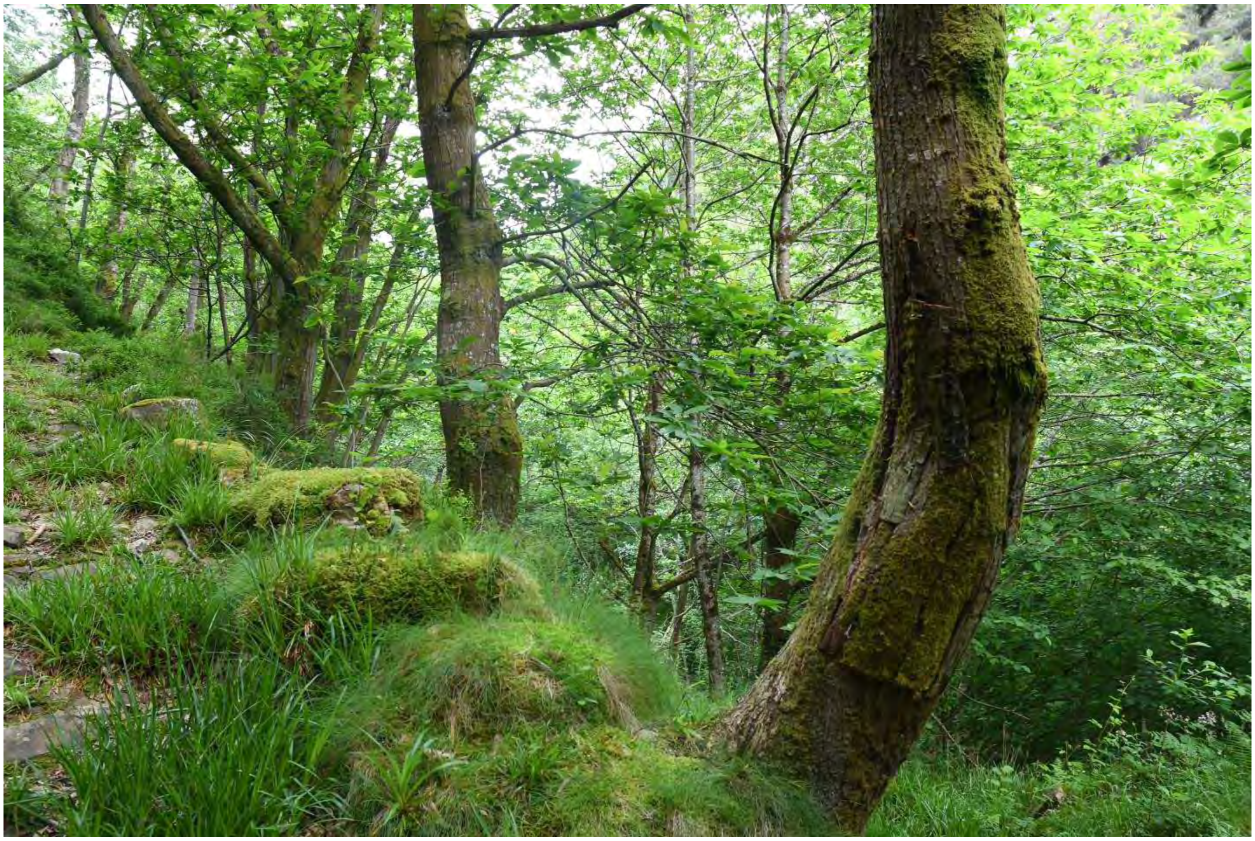

17c.

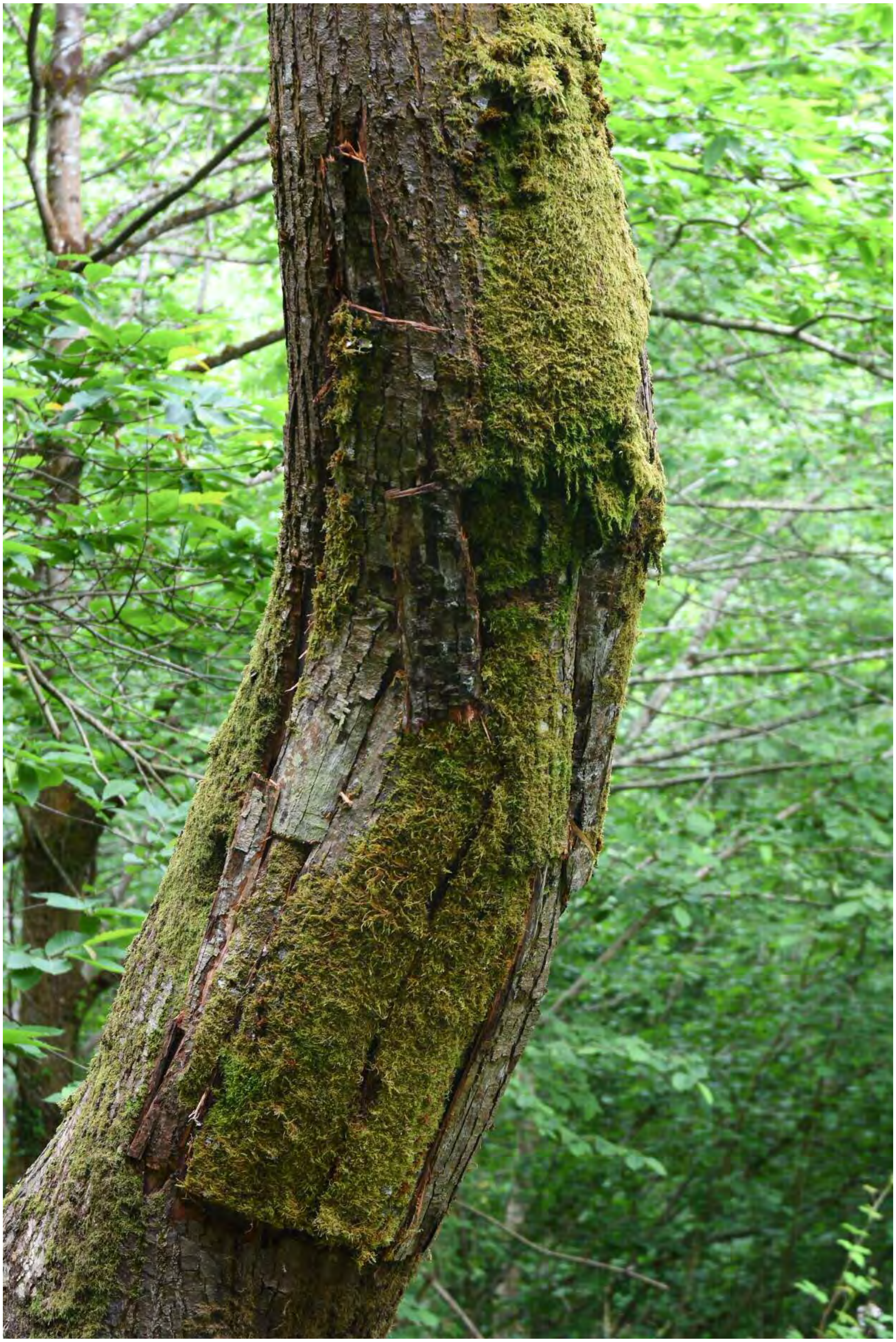

18a.

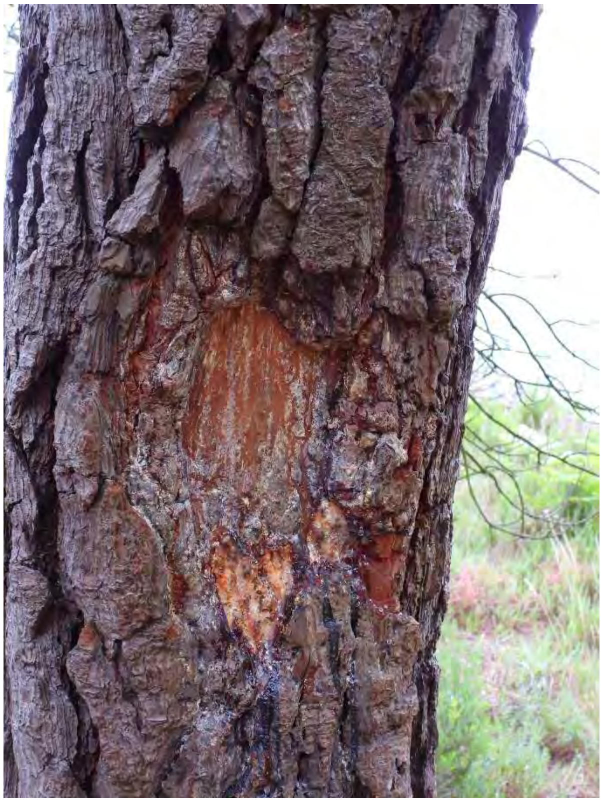

18b.

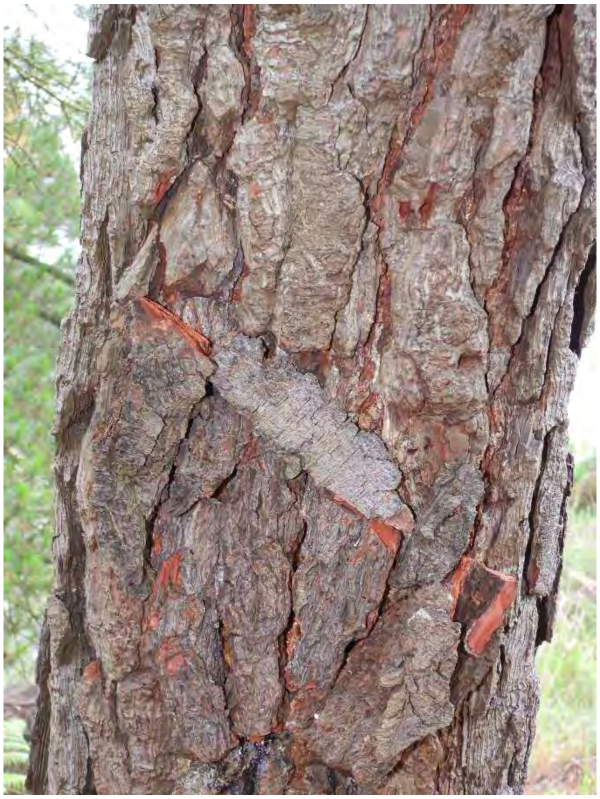

19a.

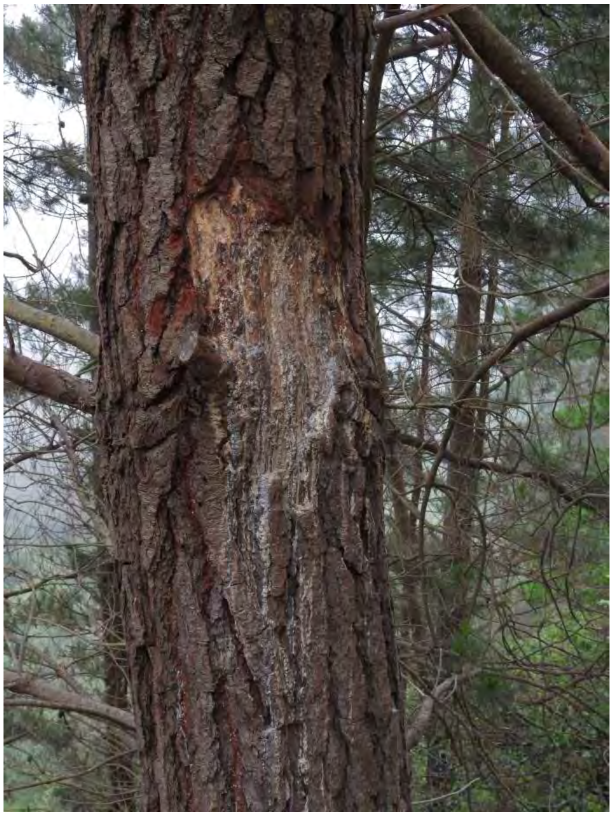

19b.

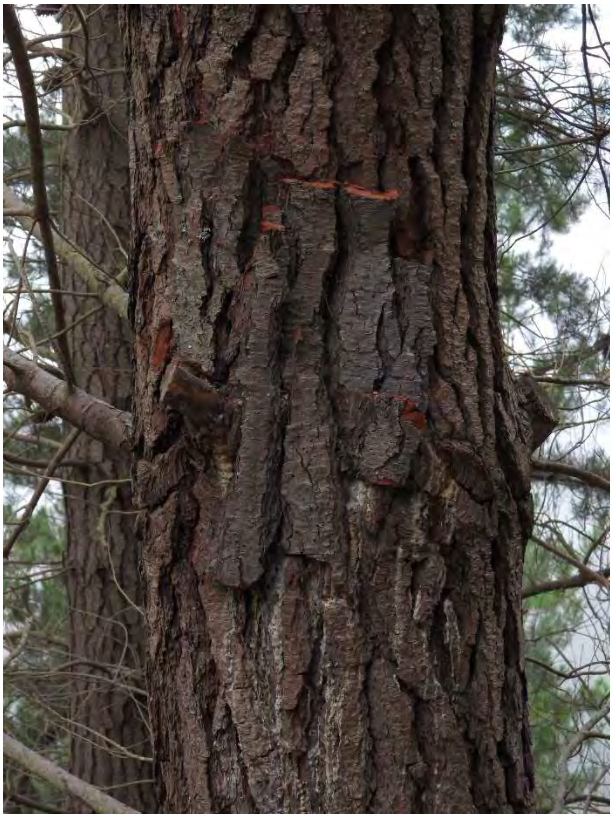

20a.

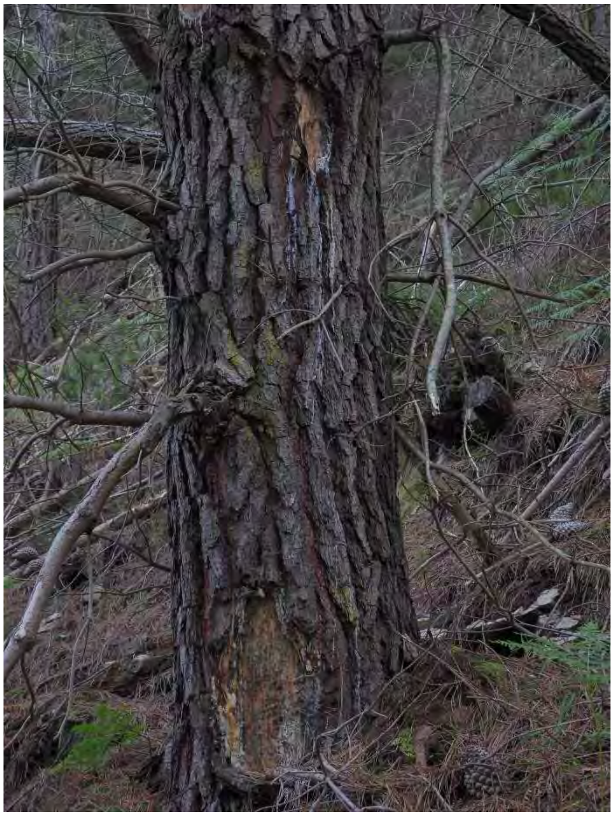

20b.

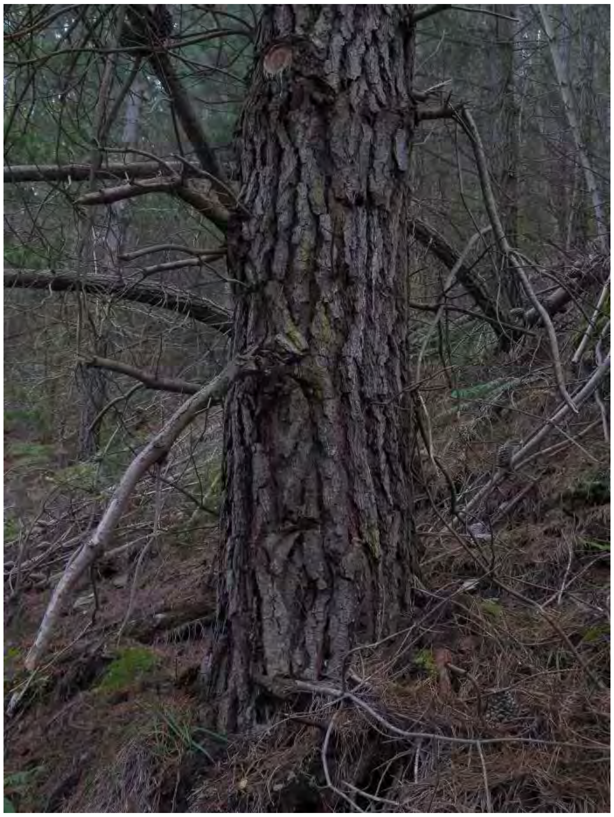

20c.

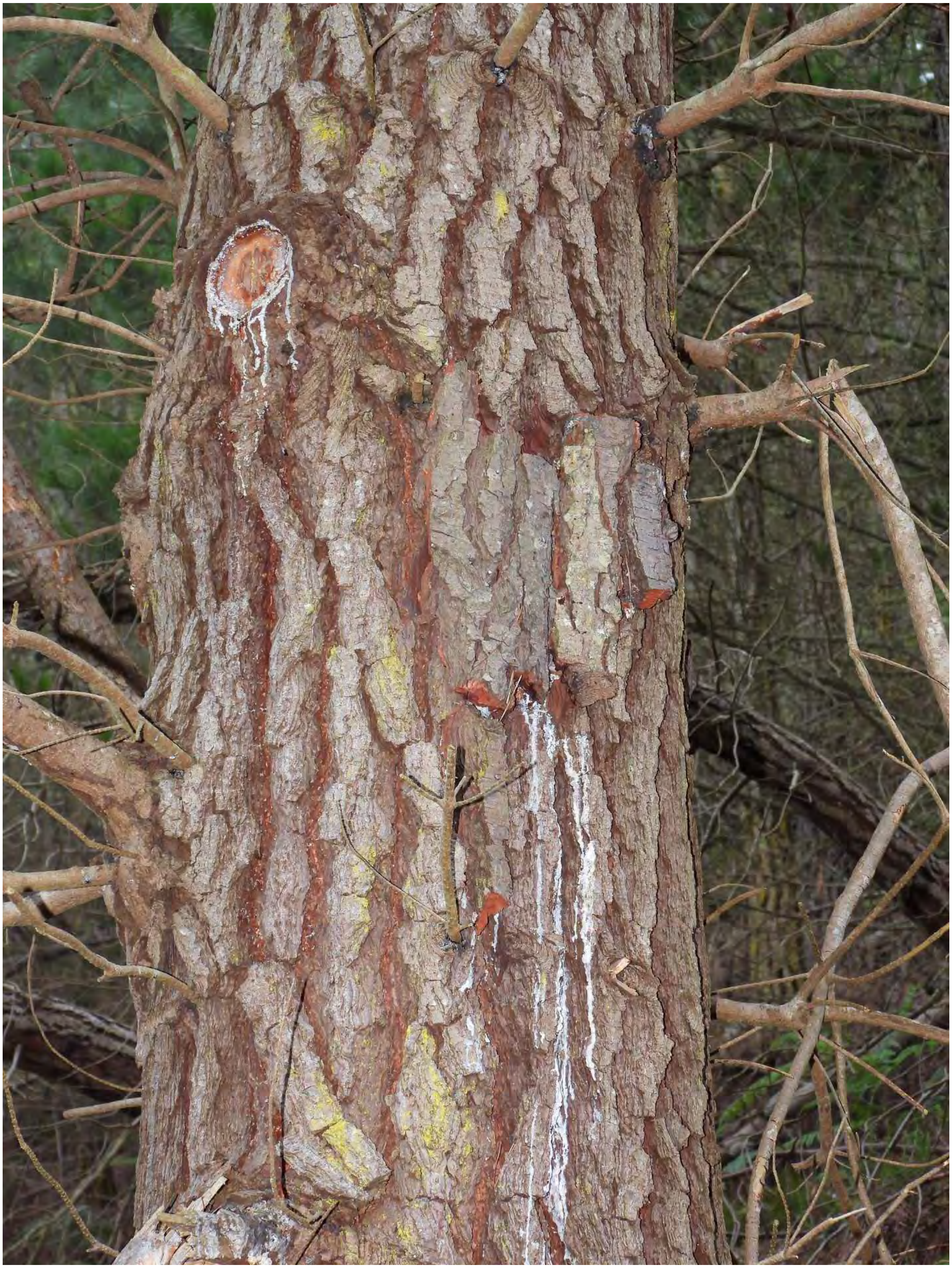

21a.

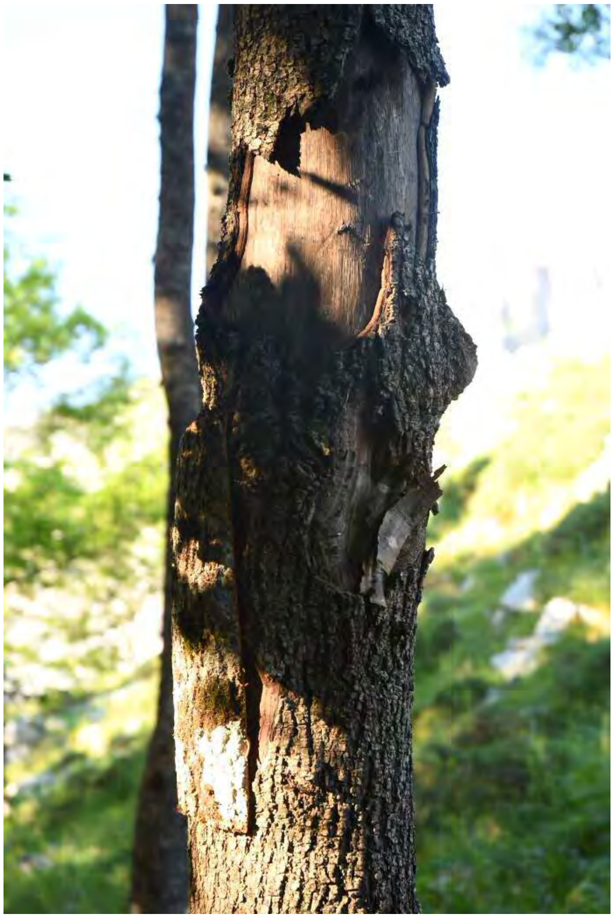

21b.

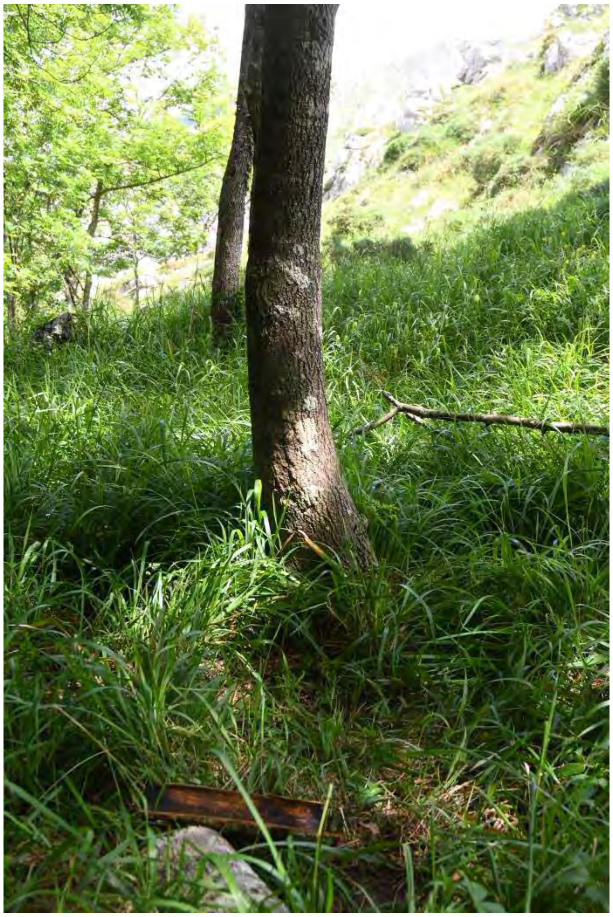

21c.

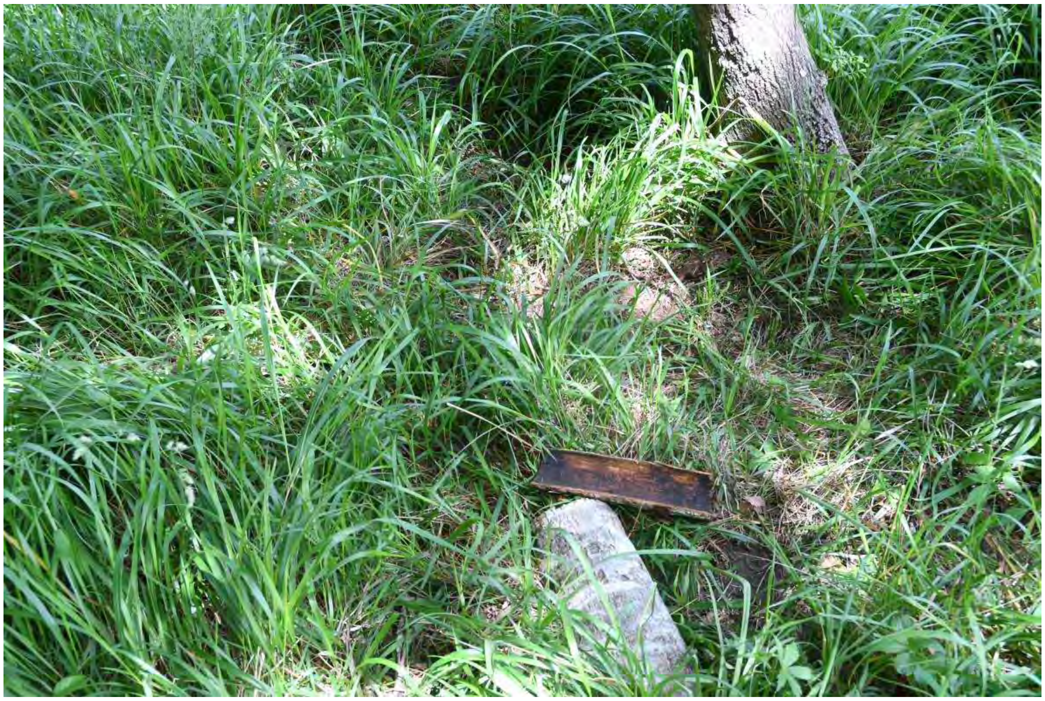

21d.

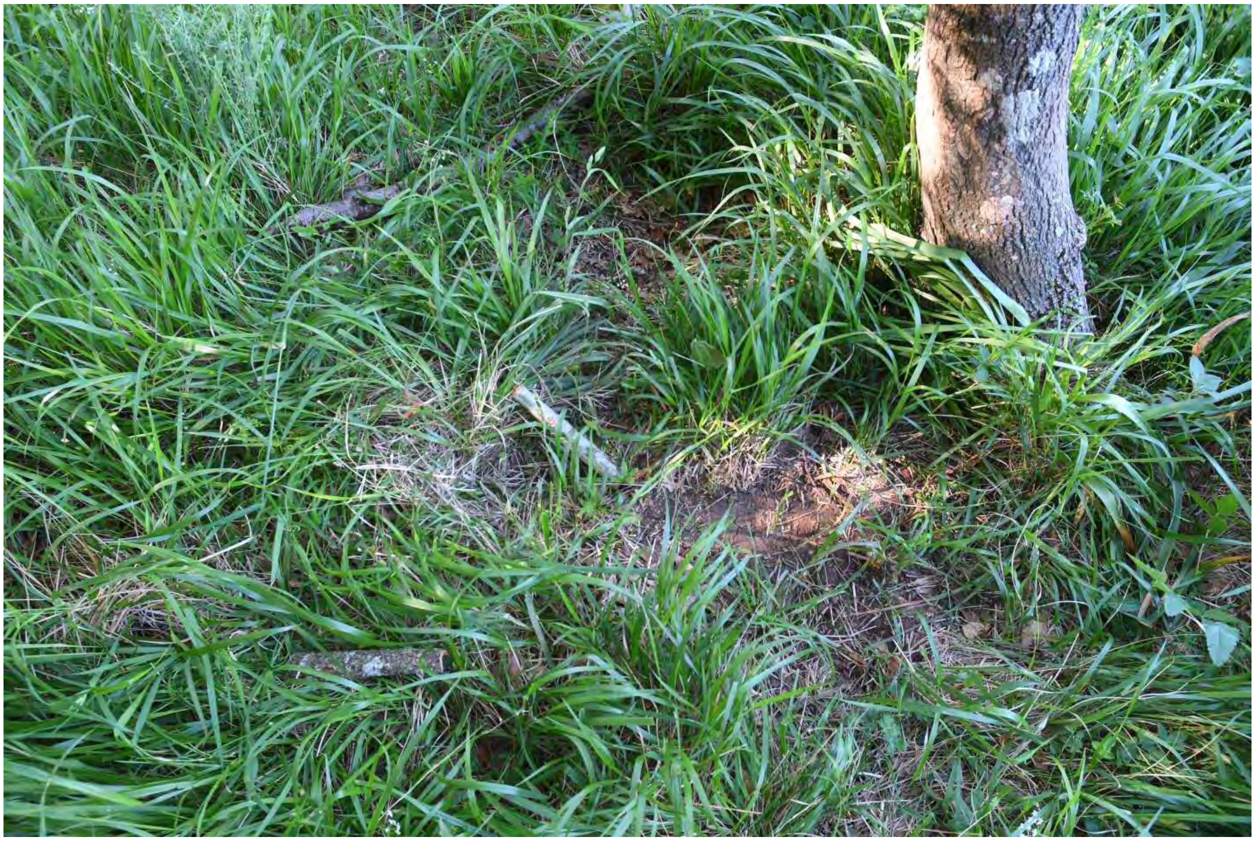

Supplement: Supplementary file 8 — Supplementary Figure 6. [file 41598_2021_88472_MOESM8_ESM.pdf]
