# Supplementary material for: Visual marking in mammals first proved by manipulations of brown bear tree debarking
Source: Sci Rep. 2021 May 4;11:9492. doi: 10.1038/s41598-021-88472-5 (PMC8096968; doi:10.1038/s41598-021-88472-5)

Visual marking in mammals first proved by manipulations of brown bear tree debarking

Vincenzo Penteriani[1*¶], Enrique González-Bernardo[1,2¶], Alfonso Hartasánchez[3], Héctor Ruiz-Villar[1], Ana Morales-González[4], Andrés Ordiz[5], Giulia Bombieri[6], Juan Díaz García[7], David Cañedo[7], Chiara Bettega[1], María del Mar Delgado[1]

1. Research Unit of Biodiversity (UMIB, CSIC-UO-PA), Mieres Campus, 33600 Mieres, Spain

2. Pyrenean Institute of Ecology (IPE), C.S.I.C., Avda. Montañana 1005, 50059 Zaragoza, Spain

3. FAPAS Fondo para la Protección de los Animales Salvajes, Ctra. AS-228, km 8,9 – Tuñón, 33115 Santo Adriano, Asturias, Spain.

4. Estación Biológica de Doñana, C.S.I.C., Department of Conservation Biology, Avda. Americo Vespucio 26, 41092 Sevilla, Spain

5. Faculty of Environmental Sciences and Natural Resource Management, Norwegian University of Life Sciences, Postbox 5003, NO-1432, Ås, Norway

6. MUSE - Museo delle Scienze, Sezione Zoologia dei Vertebrati, Corso del Lavoro e della Scienza 3, I-38123, Trento, Italy.

7. Consejería de Ordenación del Territorio, Infraestructuras y Medio Ambiente, Dirección General de Biodiversidad, Principado de Asturias, Oviedo, Spain

*Correspondence author: v.penteriani@csic.es

¶ These authors contributed equally to this work

Extended Data Fig. 7 | Removal of a bear mark manipulation covering an old visual signal. From 1 to 10: the moment (end of May 2020) when a brown bear first rubs itself against the tree and then removes the bark (picture 10) that we used to cover an old visual mark (red circle in picture 4). Pictures 11 and 12 show the visual mark after the bear uncovered it. Picture 13 depicts a bear rubbing again one week later, suggesting that bark manipulations did not affect bear chemical marking behaviour.

1.

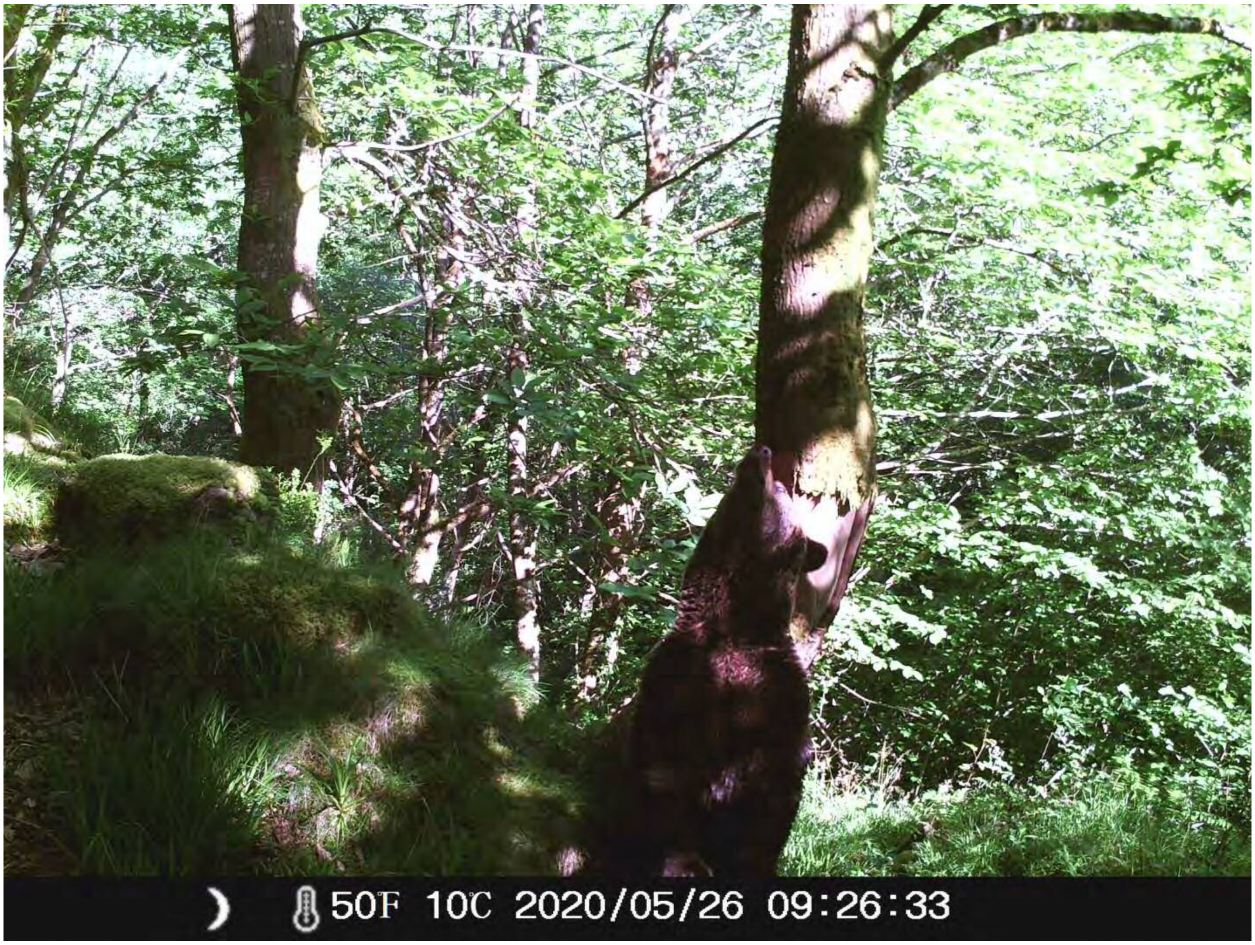

2.

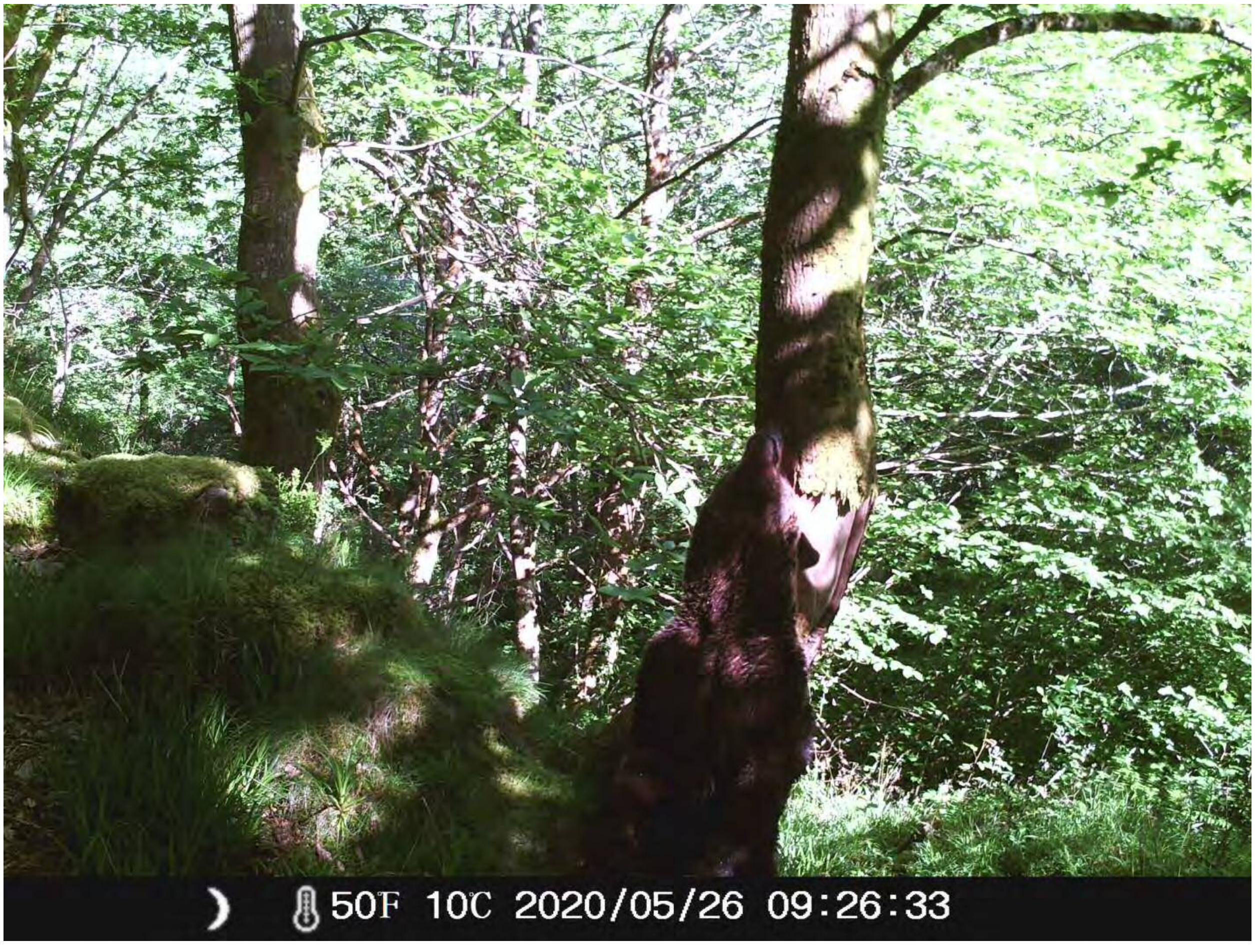

3.

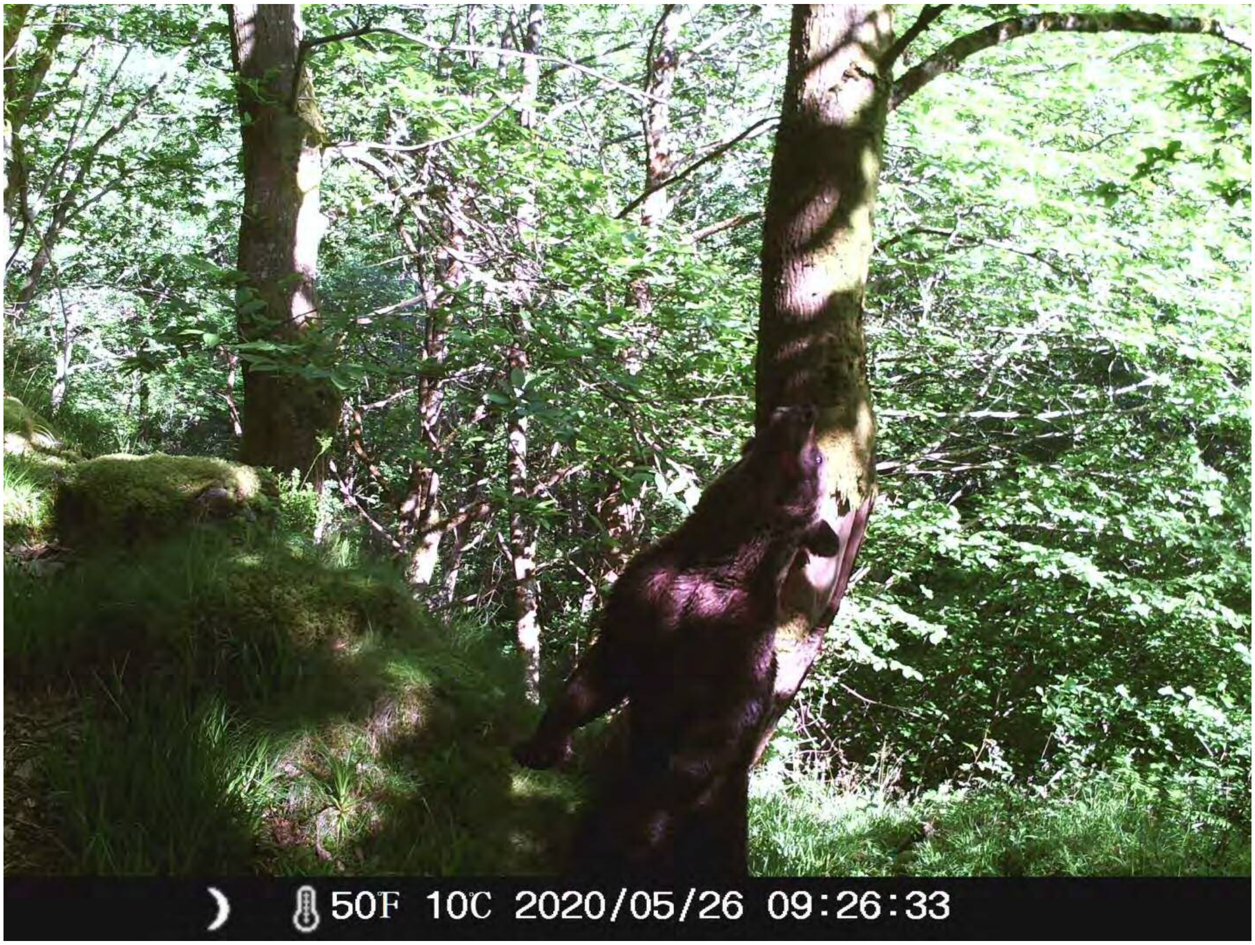

4.

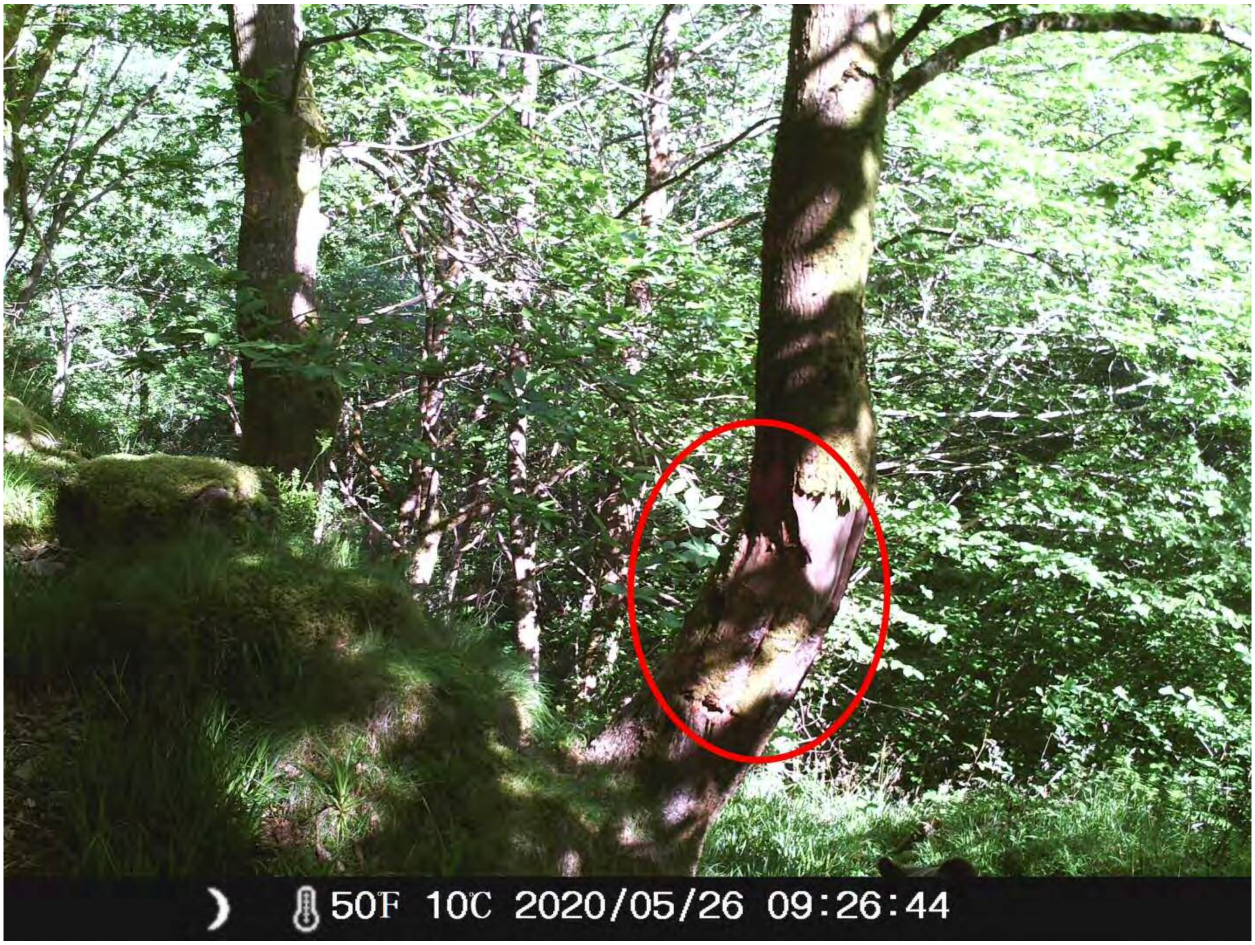

5.

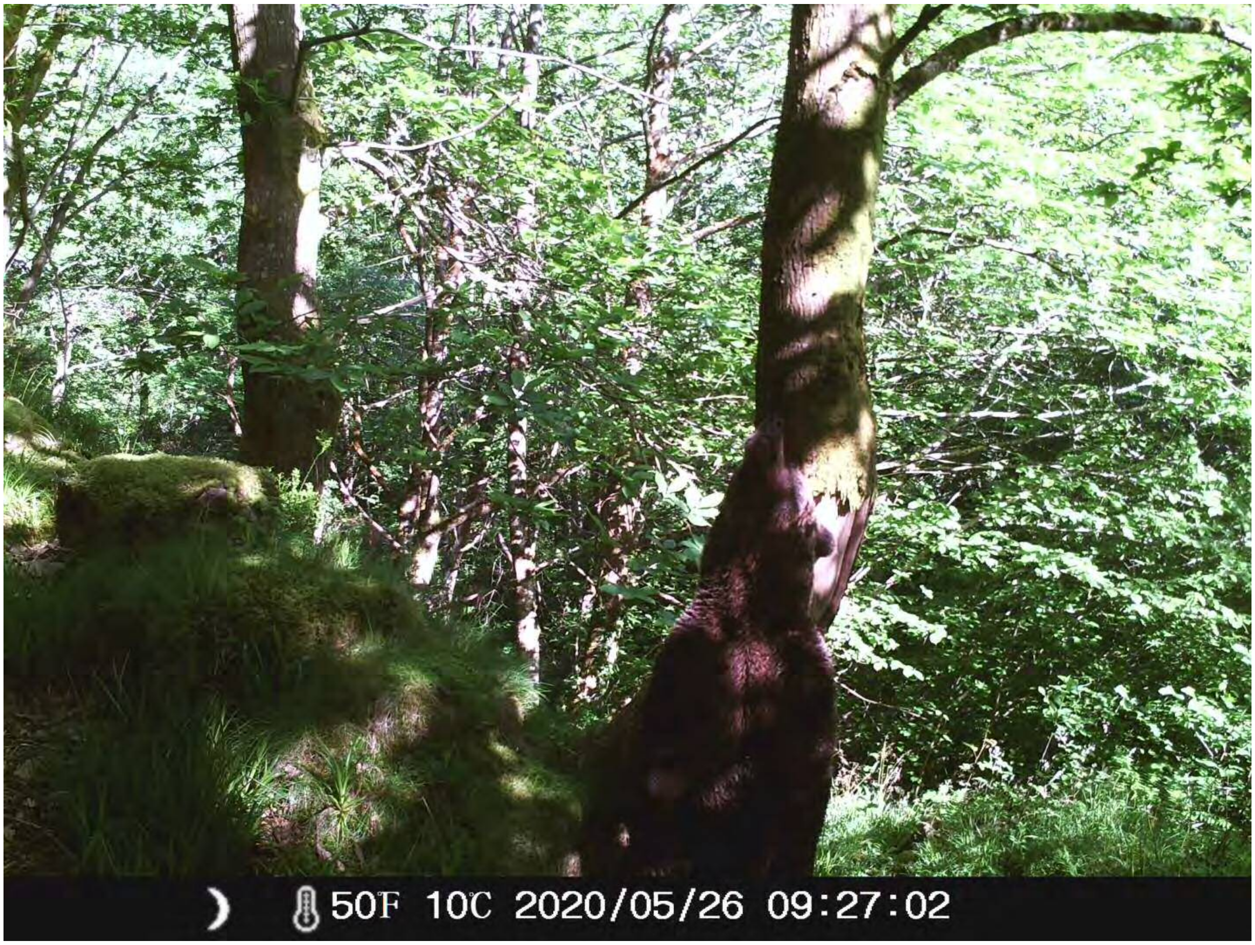

6.

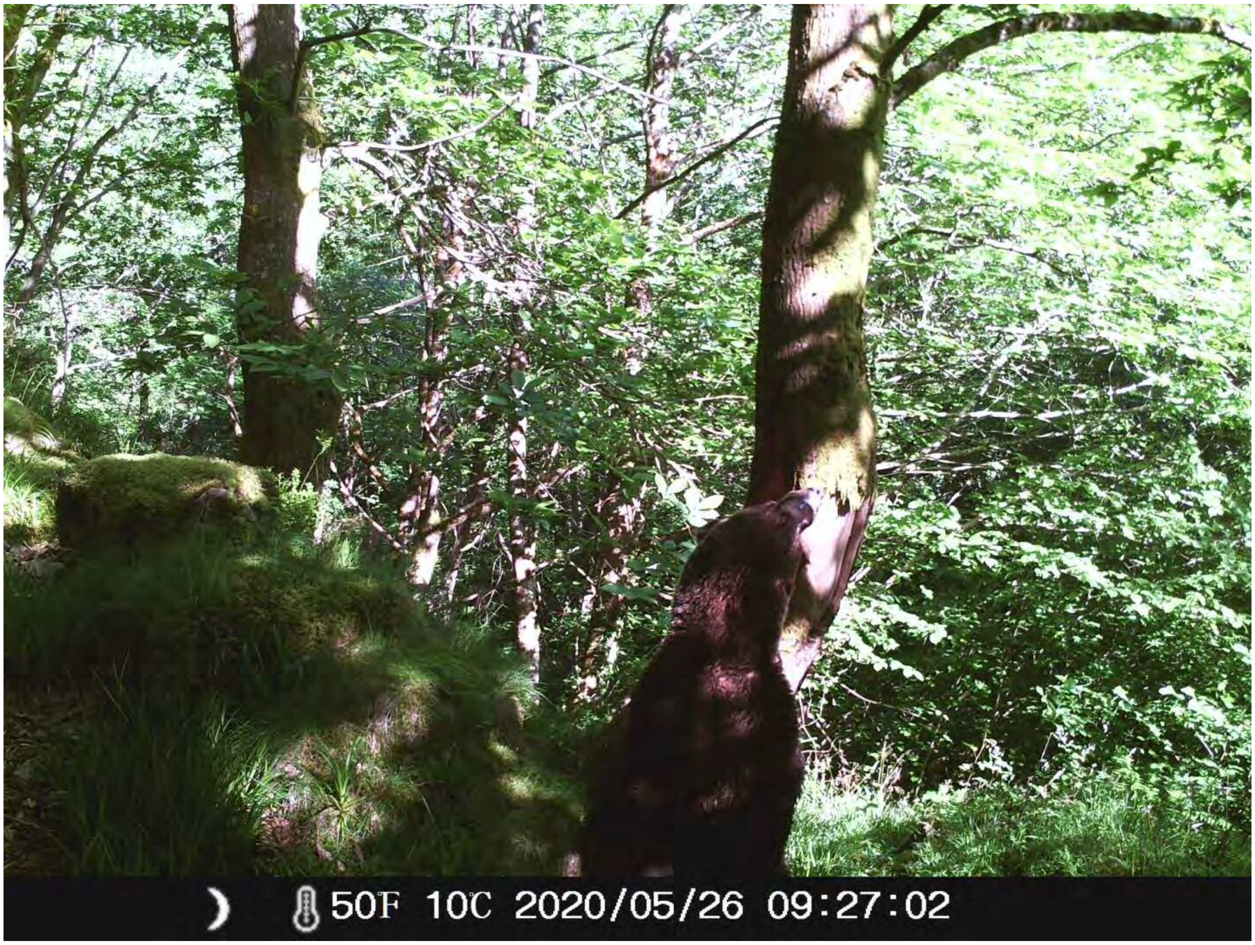

7.

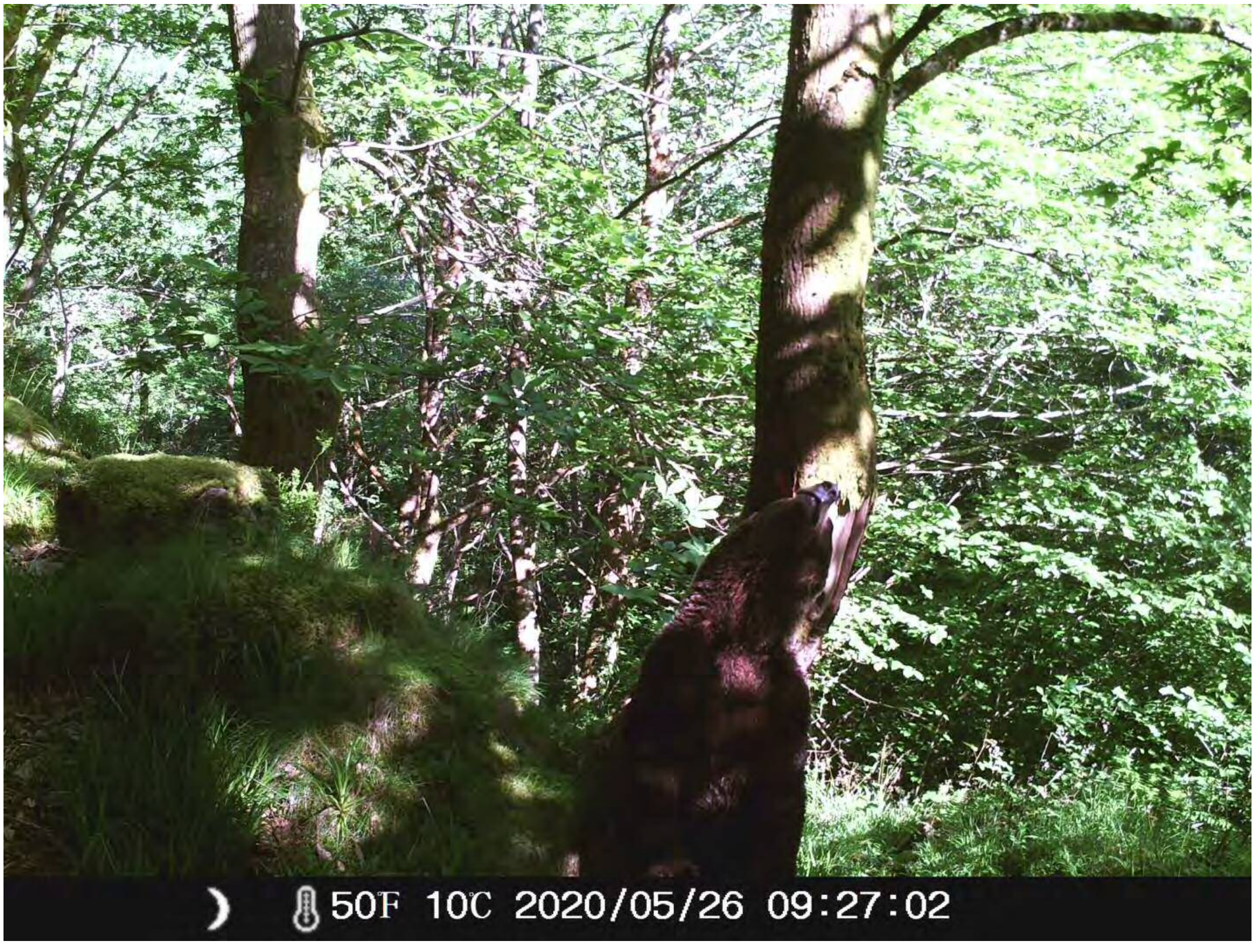

8.

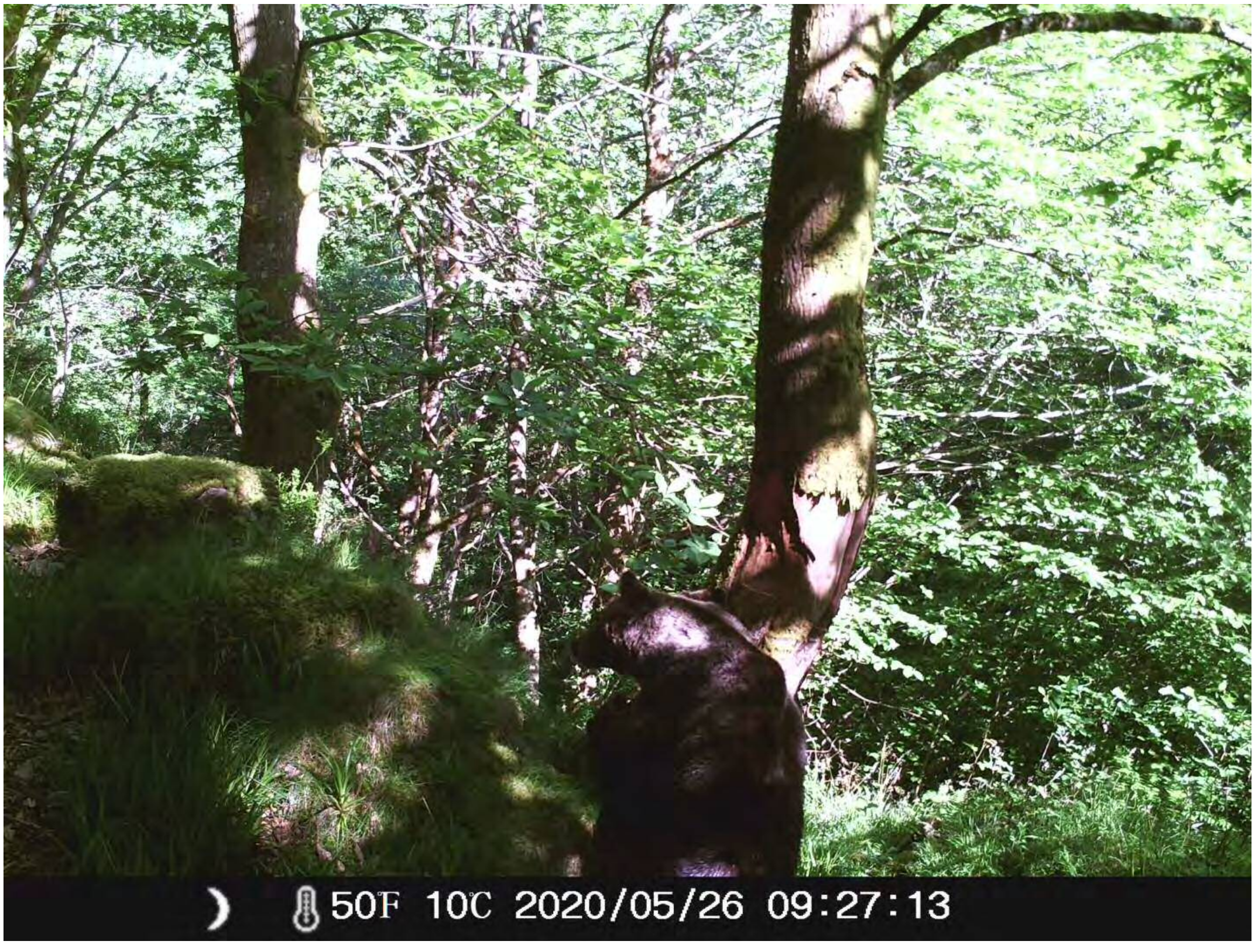

9.

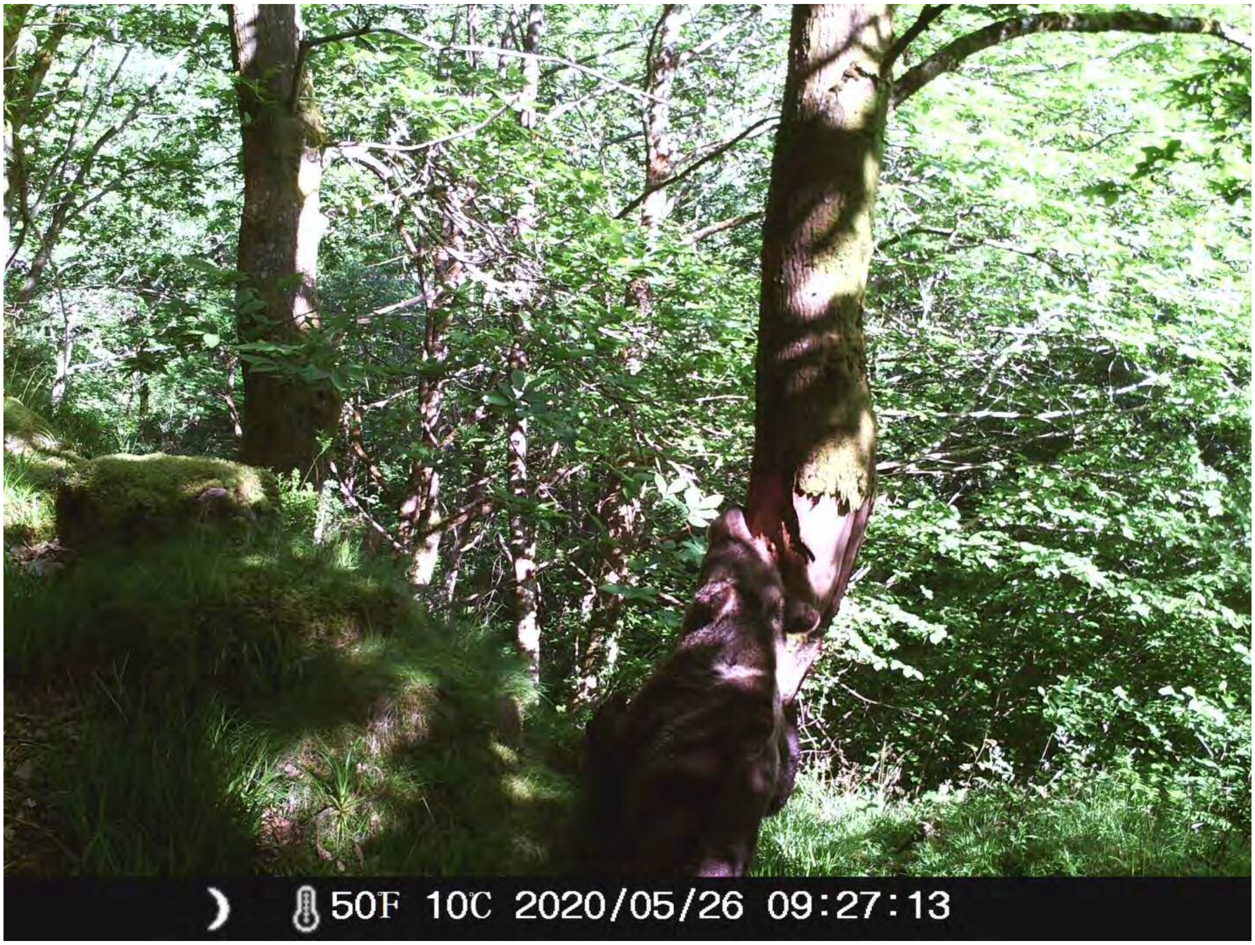

10.

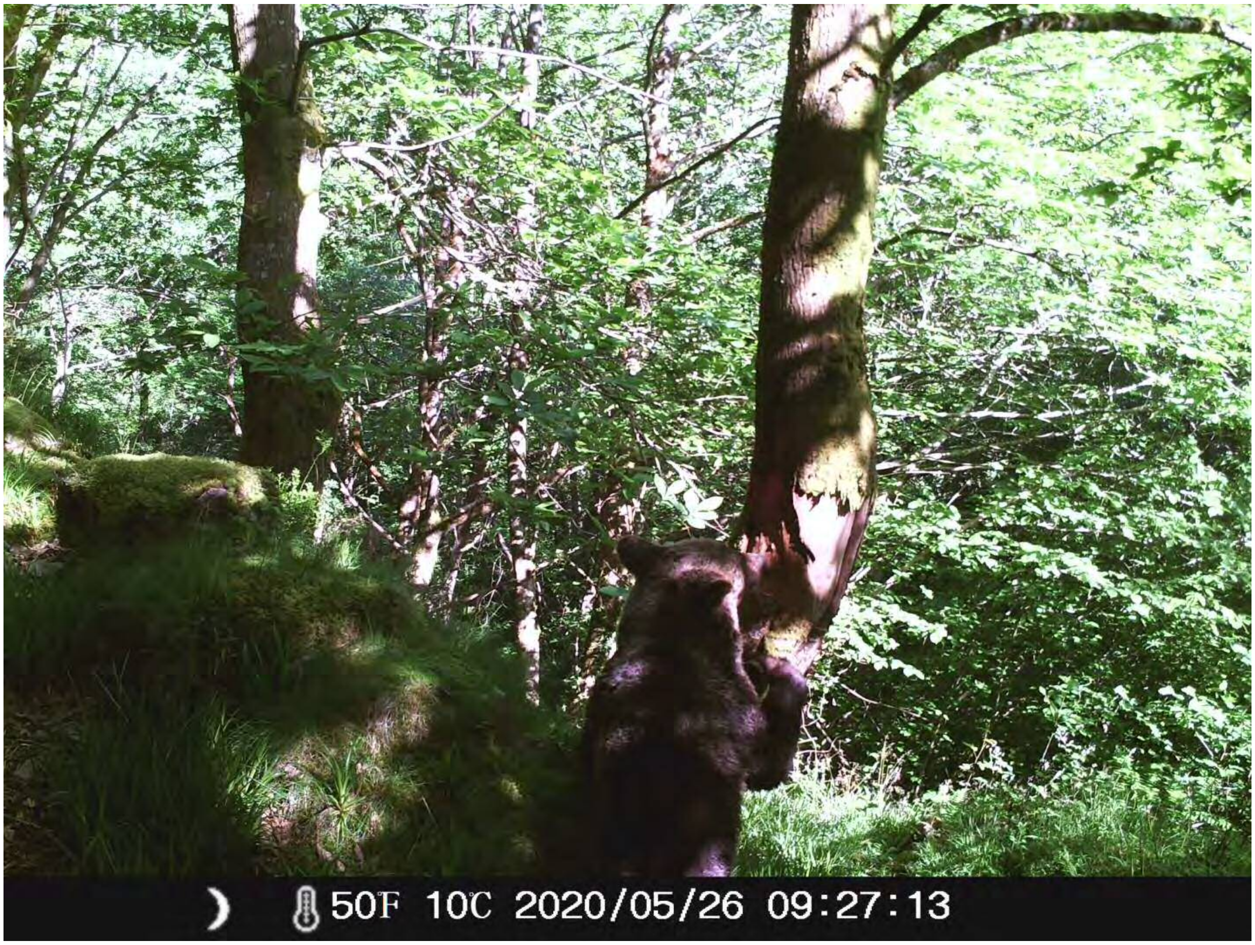

11.

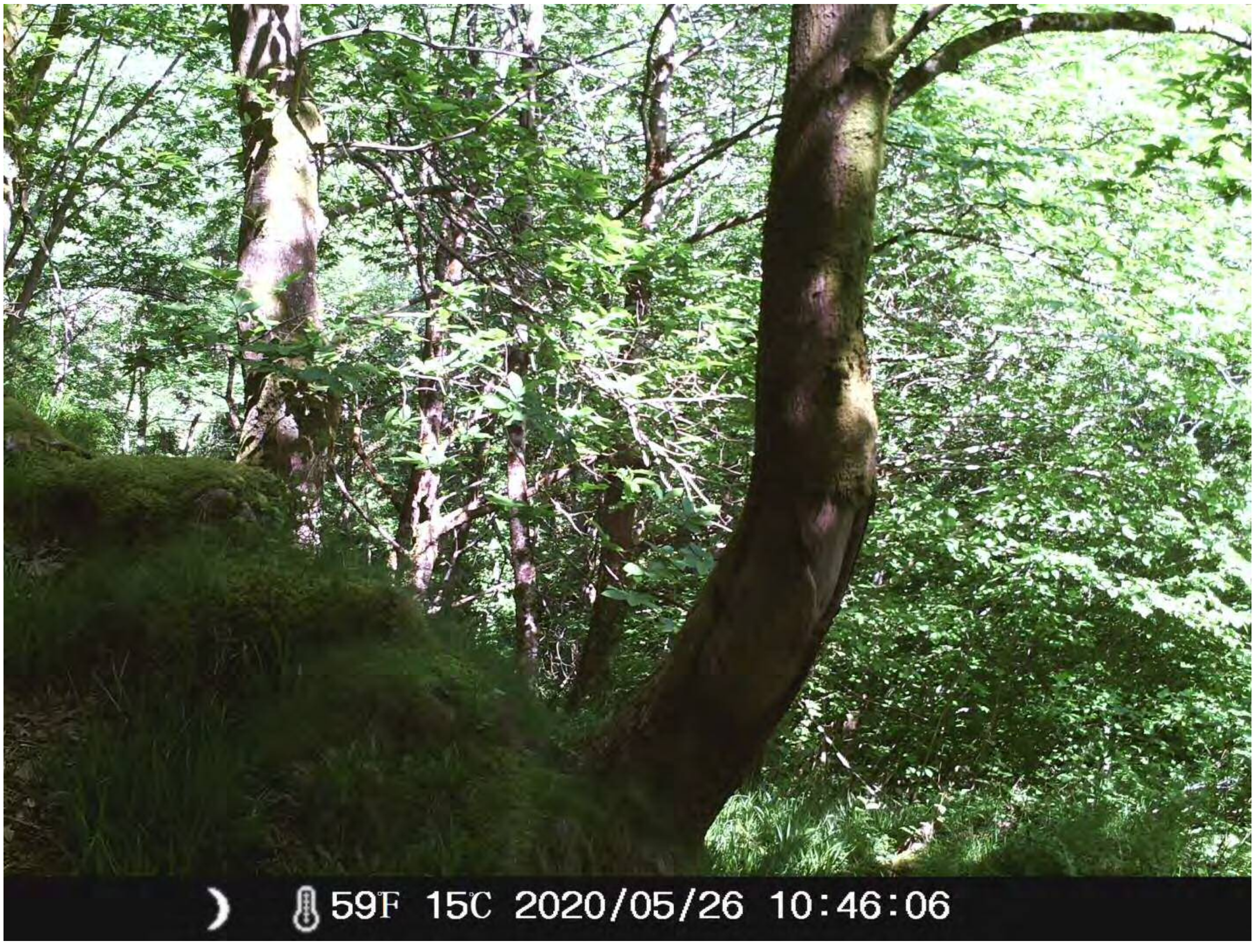

12.

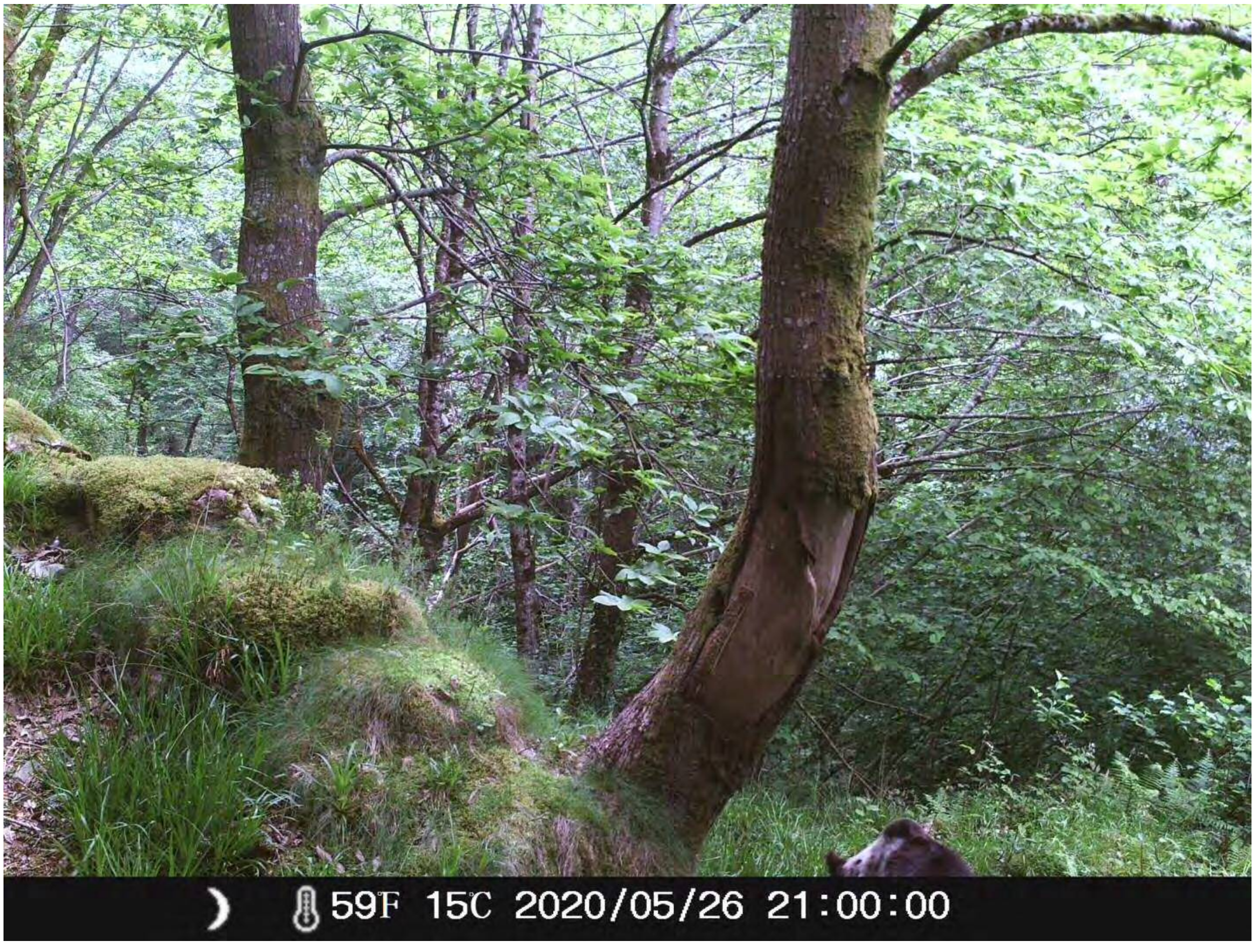

13.

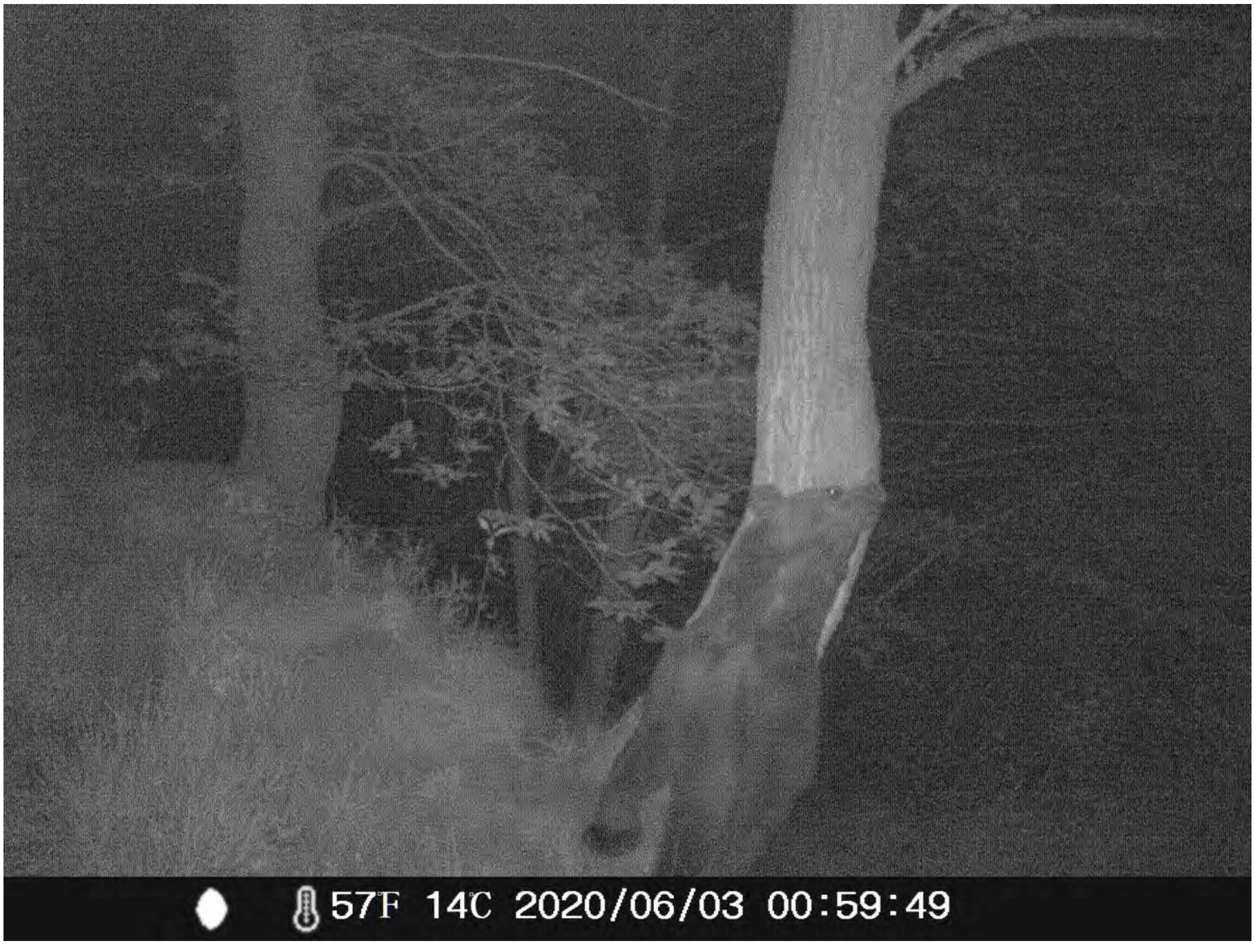

Supplement: Supplementary file 9 — Supplementary Figure 7. [file 41598_2021_88472_MOESM9_ESM.pdf]
