# Supplementary material for: Visual marking in mammals first proved by manipulations of brown bear tree debarking
Source: Sci Rep. 2021 May 4;11:9492. doi: 10.1038/s41598-021-88472-5 (PMC8096968; doi:10.1038/s41598-021-88472-5)

Visual marking in mammals first proved by manipulations of brown bear tree debarking

Vincenzo Penteriani[1*¶], Enrique González-Bernardo[1,2¶], Alfonso Hartasánchez[3], Héctor Ruiz-Villar[1], Ana Morales-González[4], Andrés Ordiz[5], Giulia Bombieri[6], Juan Díaz García[7], David Cañedo[7], Chiara Bettega[1], María del Mar Delgado[1]

1. Research Unit of Biodiversity (UMIB, CSIC-UO-PA), Mieres Campus, 33600 Mieres, Spain

2. Pyrenean Institute of Ecology (IPE), C.S.I.C., Avda. Montañana 1005, 50059 Zaragoza, Spain

3. FAPAS Fondo para la Protección de los Animales Salvajes, Ctra. AS-228, km 8,9 – Tuñón, 33115 Santo Adriano, Asturias, Spain.

4. Estación Biológica de Doñana, C.S.I.C., Department of Conservation Biology, Avda. Americo Vespucio 26, 41092 Sevilla, Spain

5. Faculty of Environmental Sciences and Natural Resource Management, Norwegian University of Life Sciences, Postbox 5003, NO-1432, Ås, Norway

6. MUSE - Museo delle Scienze, Sezione Zoologia dei Vertebrati, Corso del Lavoro e della Scienza 3, I-38123, Trento, Italy.

7. Consejería de Ordenación del Territorio, Infraestructuras y Medio Ambiente, Dirección General de Biodiversidad, Principado de Asturias, Oviedo, Spain

*Correspondence author: v.penteriani@csic.es

¶ These authors contributed equally to this work

Extended Data Fig. 8 | Brown bear visual markings also occur on trees other than rub trees. 1. A bark strip recently removed by a bear from a small birch trunk, which was found on the ground at the base of the trunk on the 2nd of May 2020. This tree was not used for rubbing. 2. Detail of the removed bark. 3-4. Details of the manipulation of the visual mark by covering it with the same bark strip removed by the bear. 5. The bark strip used to cover the visual mark had been removed between the 8th and the 15th of May 2020.

1.

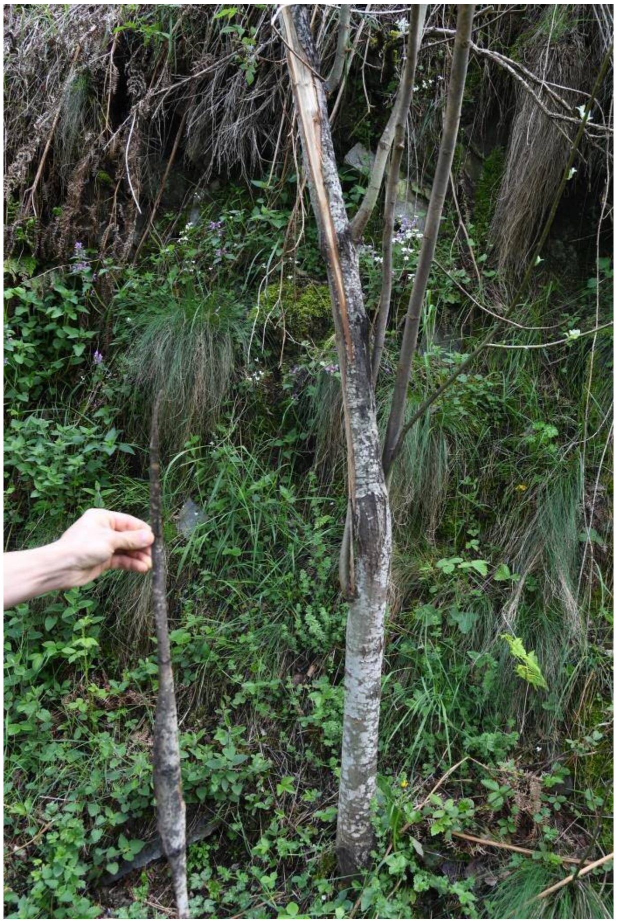

2.

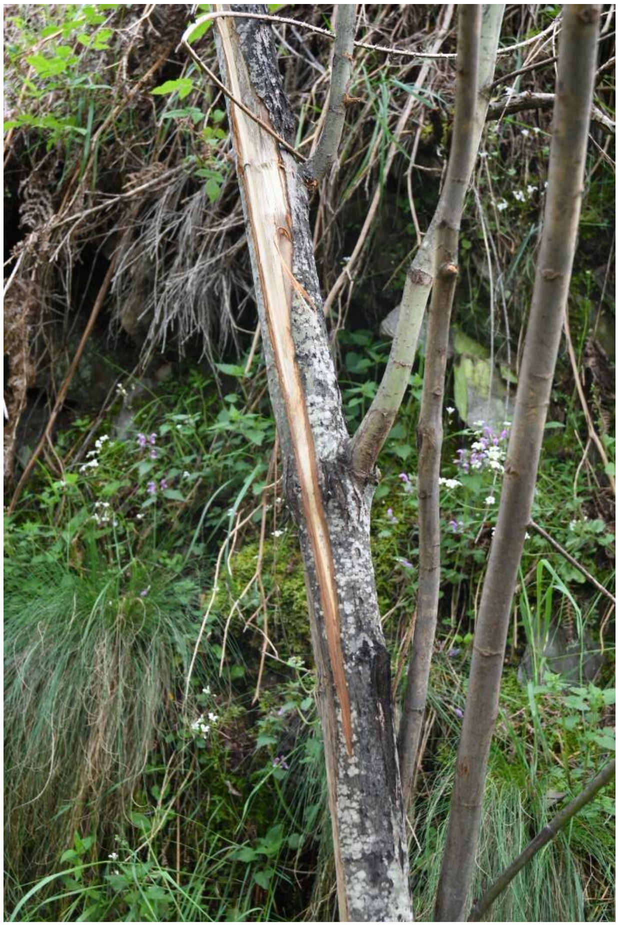

3.

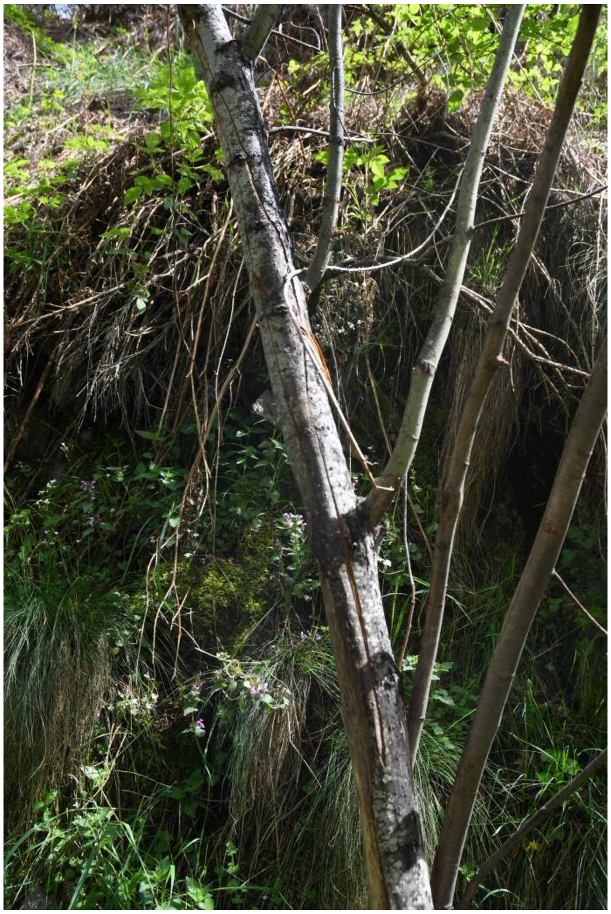

4.

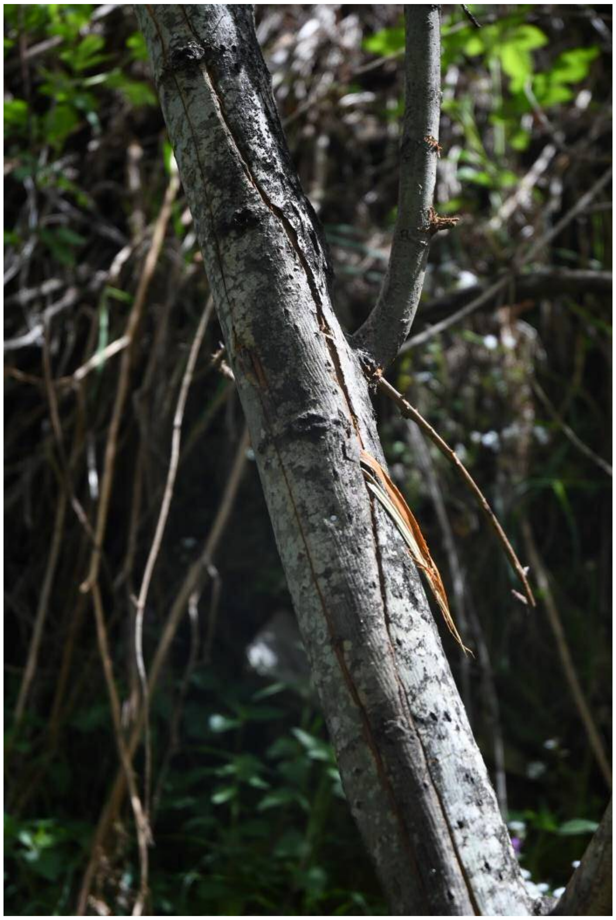

5.

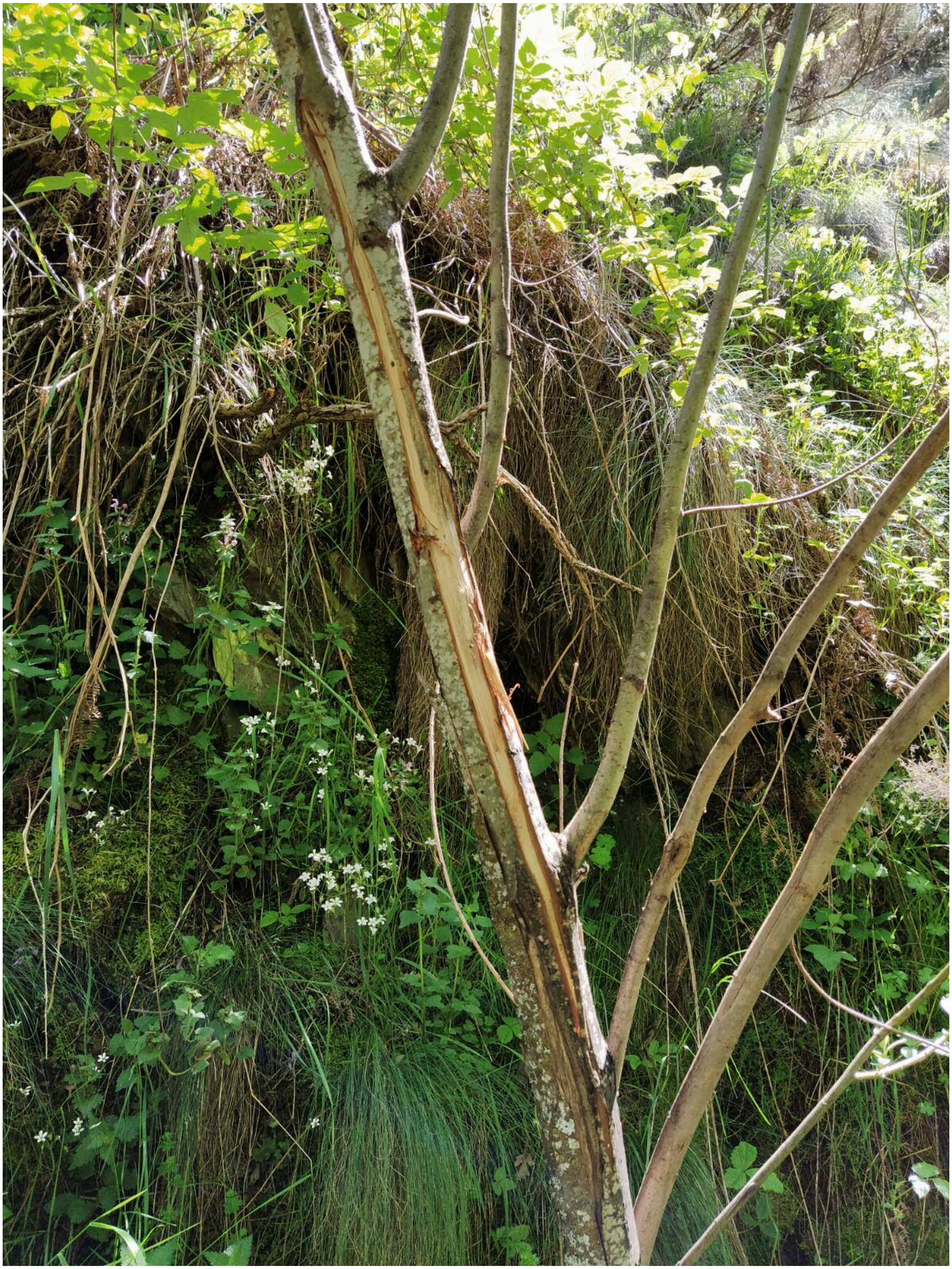

Supplement: Supplementary file 10 — Supplementary Figure 8. [file 41598_2021_88472_MOESM10_ESM.pdf]
