# Supplementary material for: Visual marking in mammals first proved by manipulations of brown bear tree debarking
Source: Sci Rep. 2021 May 4;11:9492. doi: 10.1038/s41598-021-88472-5 (PMC8096968; doi:10.1038/s41598-021-88472-5)
Supplement: Supplementary file 11 — Supplementary Figure 9. [file 41598_2021_88472_MOESM11_ESM.pdf]

Visual marking in mammals first proved by manipulations of brown bear tree debarking

Vincenzo Penteriani[1*¶], Enrique González-Bernardo[1,2¶], Alfonso Hartasánchez[3], Héctor Ruiz-Villar[1], Ana Morales-González[4], Andrés Ordiz[5], Giulia Bombieri[6], Juan Díaz García[7], David Cañedo[7], Chiara Bettega[1], María del Mar Delgado[1]

1. Research Unit of Biodiversity (UMIB, CSIC-UO-PA), Mieres Campus, 33600 Mieres, Spain

2. Pyrenean Institute of Ecology (IPE), C.S.I.C., Avda. Montañana 1005, 50059 Zaragoza, Spain

3. FAPAS Fondo para la Protección de los Animales Salvajes, Ctra. AS-228, km 8,9 – Tuñón, 33115 Santo Adriano, Asturias, Spain.

4. Estación Biológica de Doñana, C.S.I.C., Department of Conservation Biology, Avda. Americo Vespucio 26, 41092 Sevilla, Spain

5. Faculty of Environmental Sciences and Natural Resource Management, Norwegian University of Life Sciences, Postbox 5003, NO-1432, Ås, Norway

6. MUSE - Museo delle Scienze, Sezione Zoologia dei Vertebrati, Corso del Lavoro e della Scienza 3, I-38123, Trento, Italy.

7. Consejería de Ordenación del Territorio, Infraestructuras y Medio Ambiente, Dirección General de Biodiversidad, Principado de Asturias, Oviedo, Spain

*Correspondence author: v.penteriani@csic.es

¶ These authors contributed equally to this work

Extended Data Fig. 9 | Brown bear visual marking is complementary to chemical signalling. These images show one of the most typical features of brown bear visual marking, i.e., visual marks generally are on upper sections of the trunk, where even the largest adult males cannot leave their scent (chemical signalling) by performing rubbing behaviour. Here, different 'classes' of bears, from small subadults to large adult males mark (August-September 2017) the same tree, but only the adult male 'captured' by the camera trap in images 10 and 11 (and probably 12) can also cover part of the visual mark with its head when rubbing. This reinforces the possibility that: (1) visual marking is a prerogative of the largest and tallest bears (i.e., typically adult males); and (2) visual marks represent a marking behaviour additional and complementary to chemical marking, which may be distinctive to the largest individuals.

1.

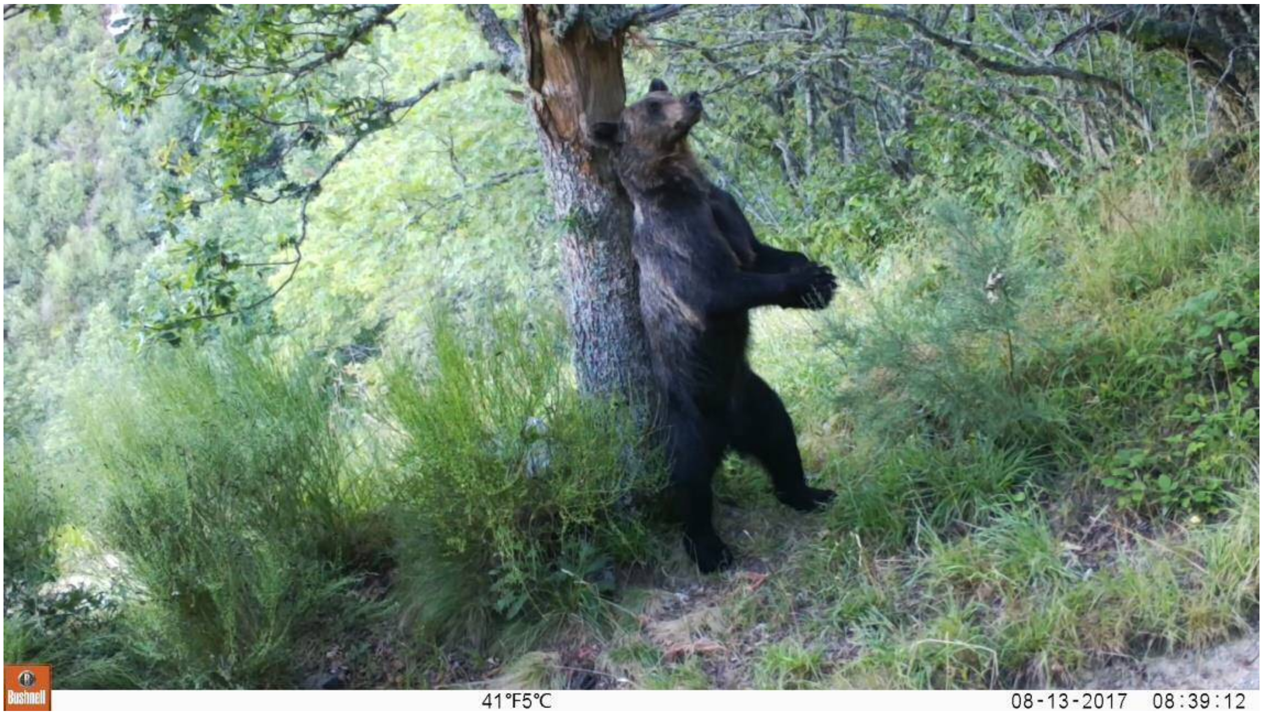

2.

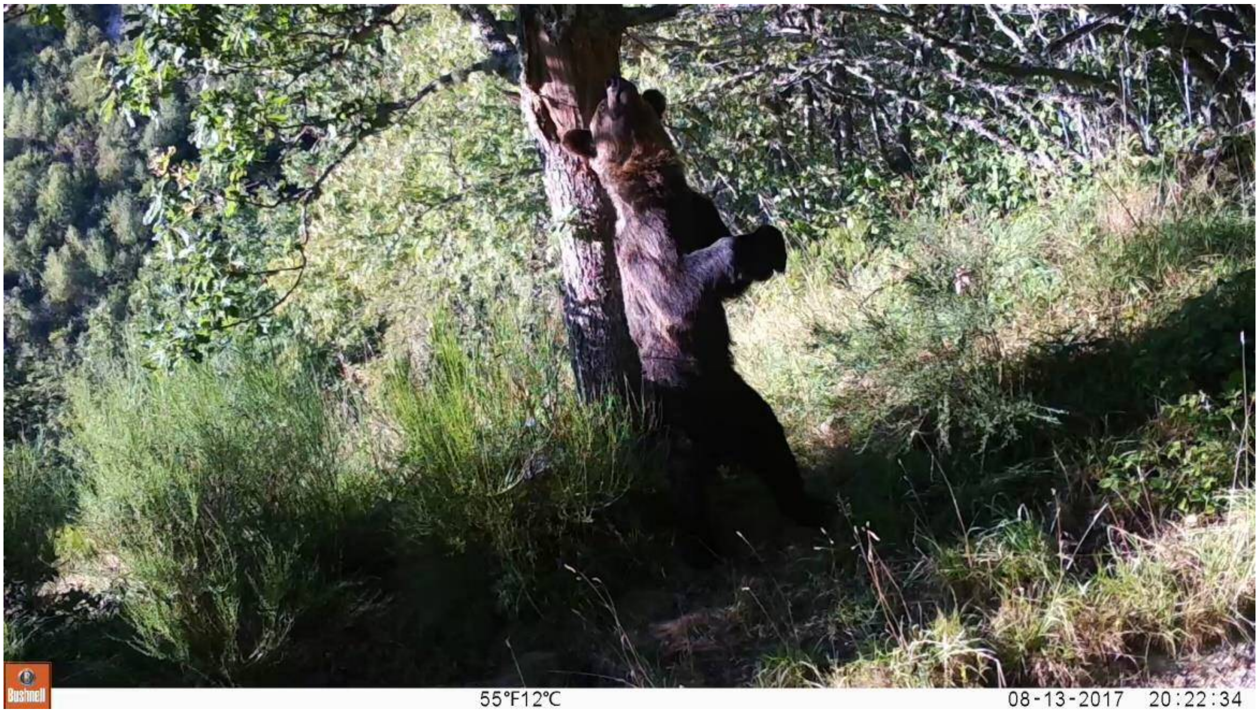
Bushnell
55°F12°C
08-13-2017 20:22:34

3.

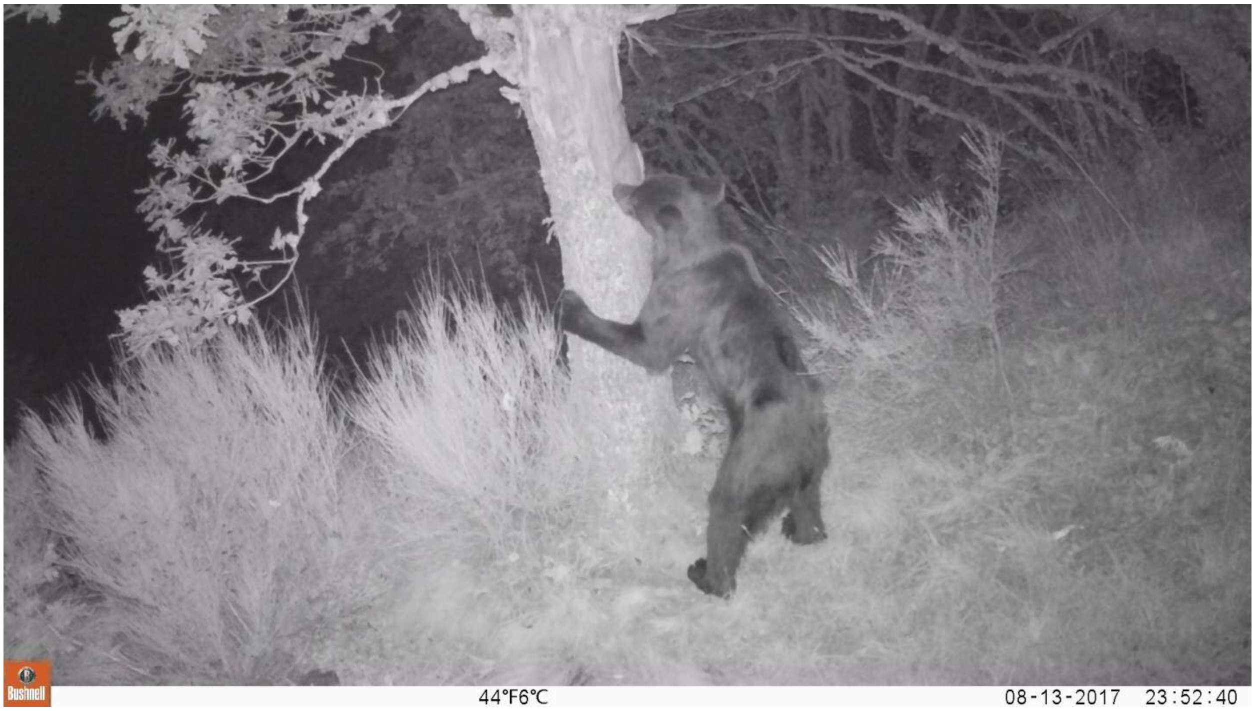
Bushnell
44°F6°C
08-13-2017 23:52:40

4.

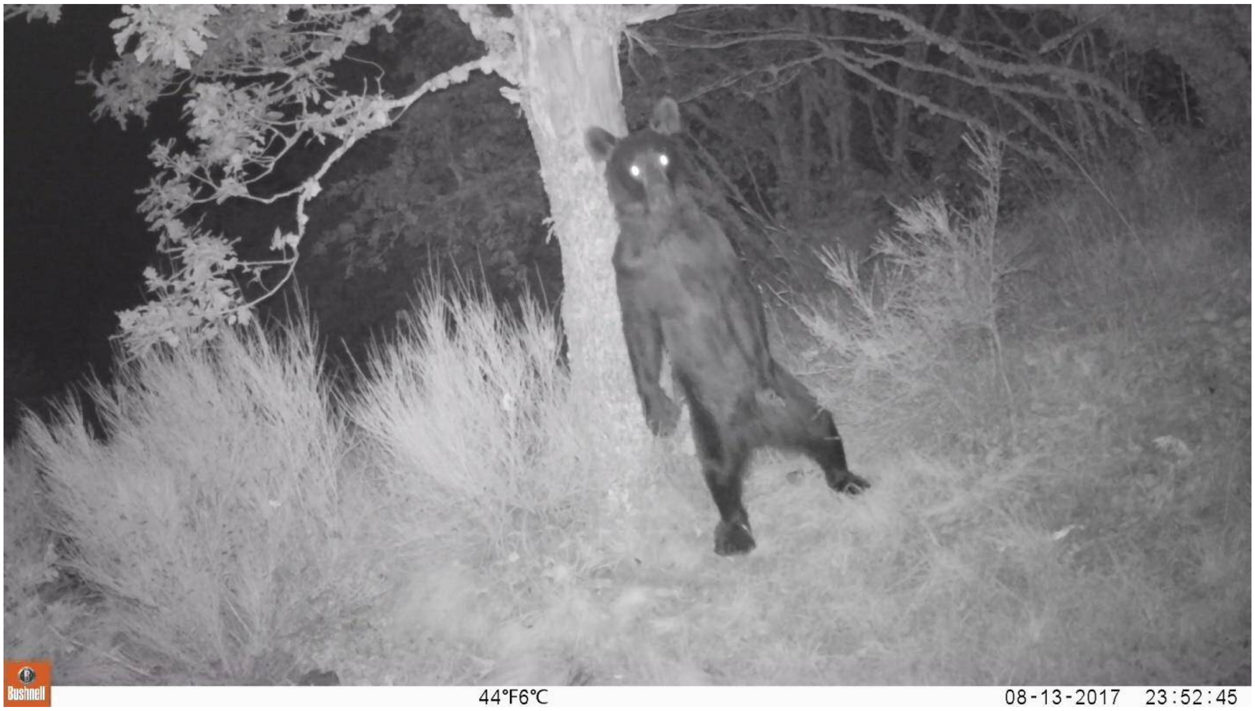

5.

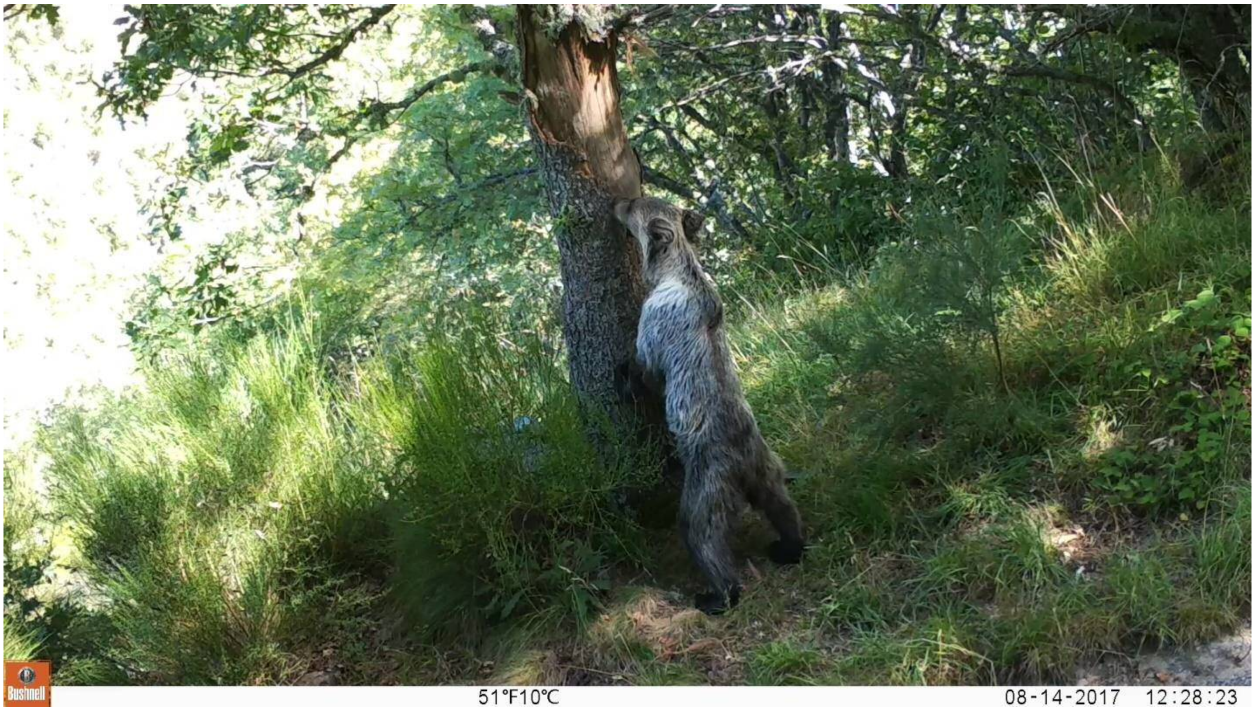
Bushnell
51°F10°C
08-14-2017 12:28:23

6.

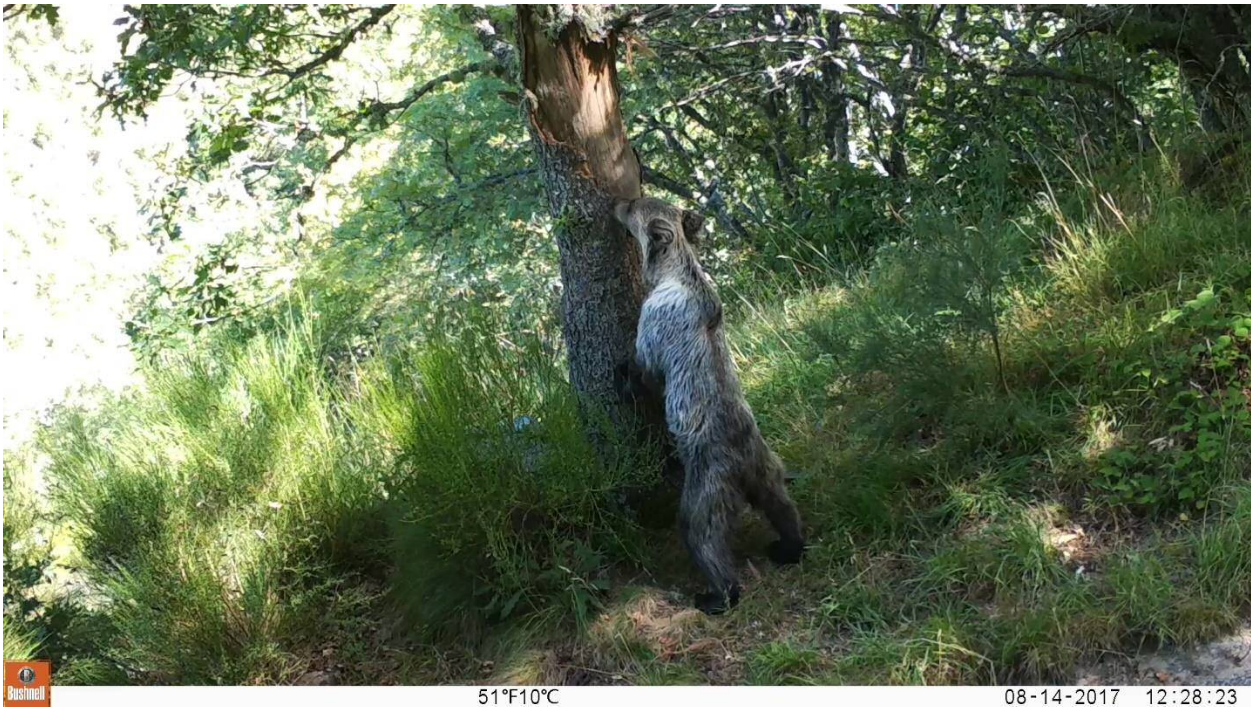
Bushnell
51°F10°C
08-14-2017 12:28:23

7.

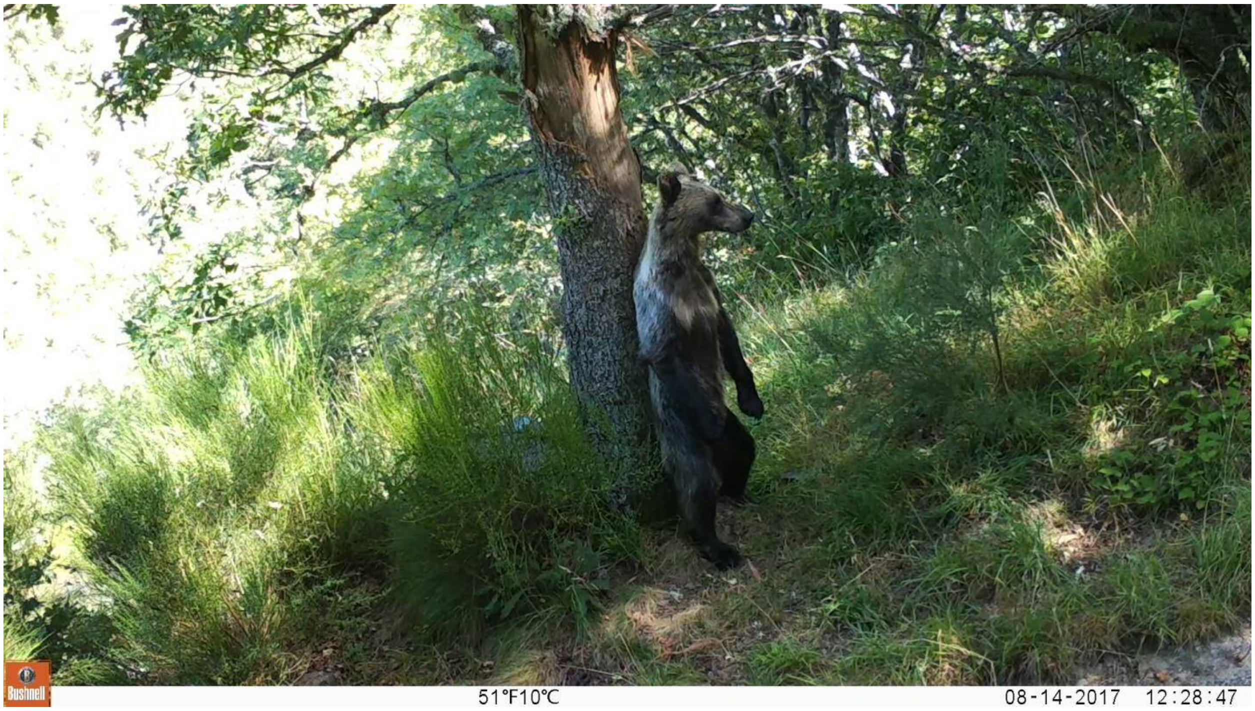

8.

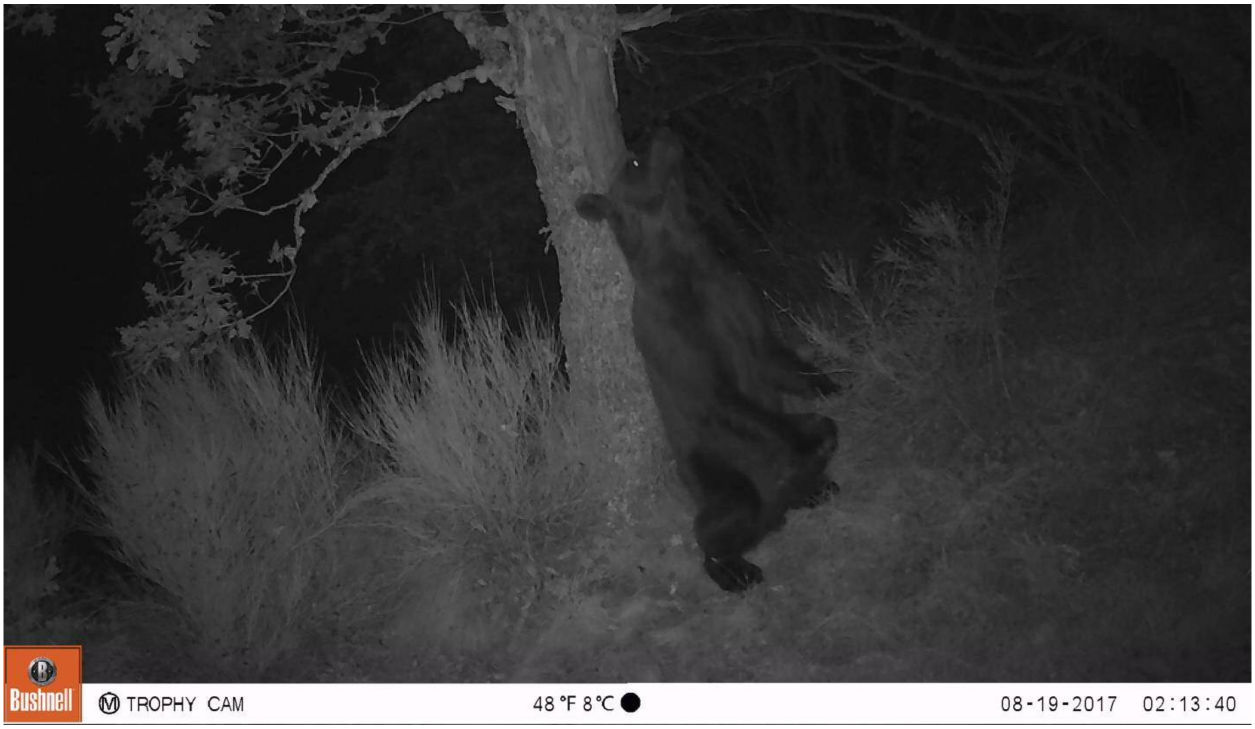

9.

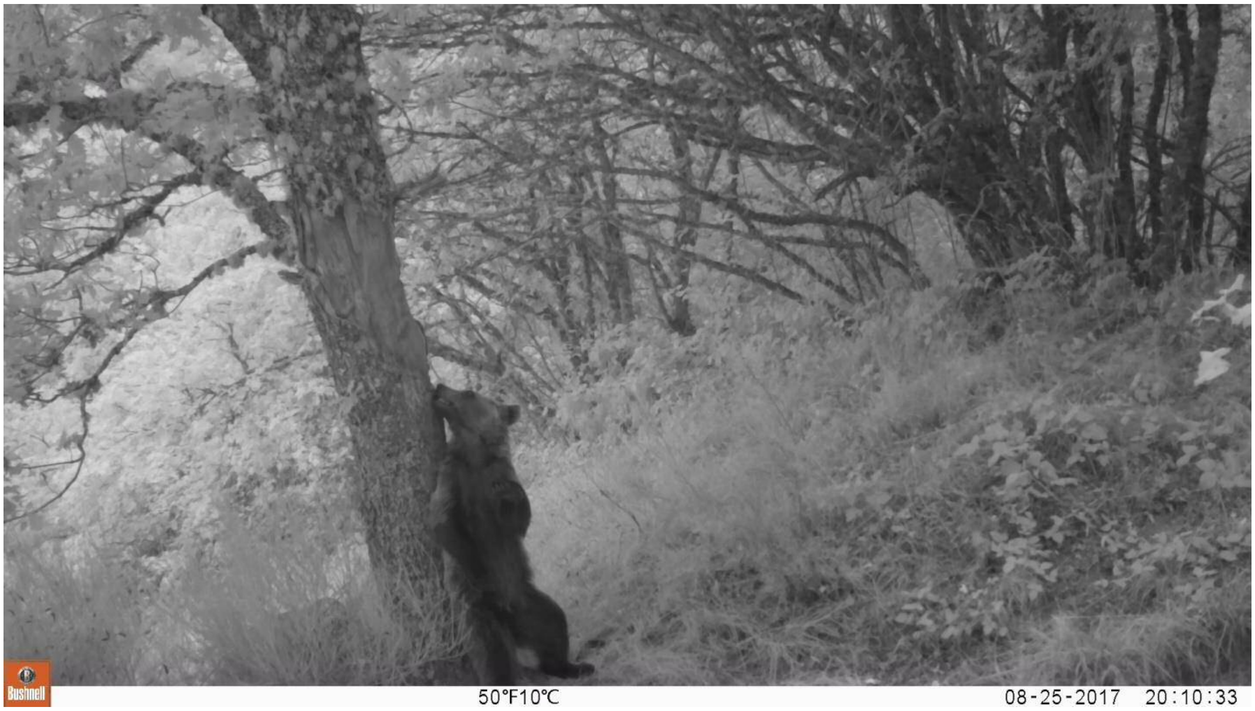
Bushnell
50°F10°C
08-25-2017 20:10:33

10.

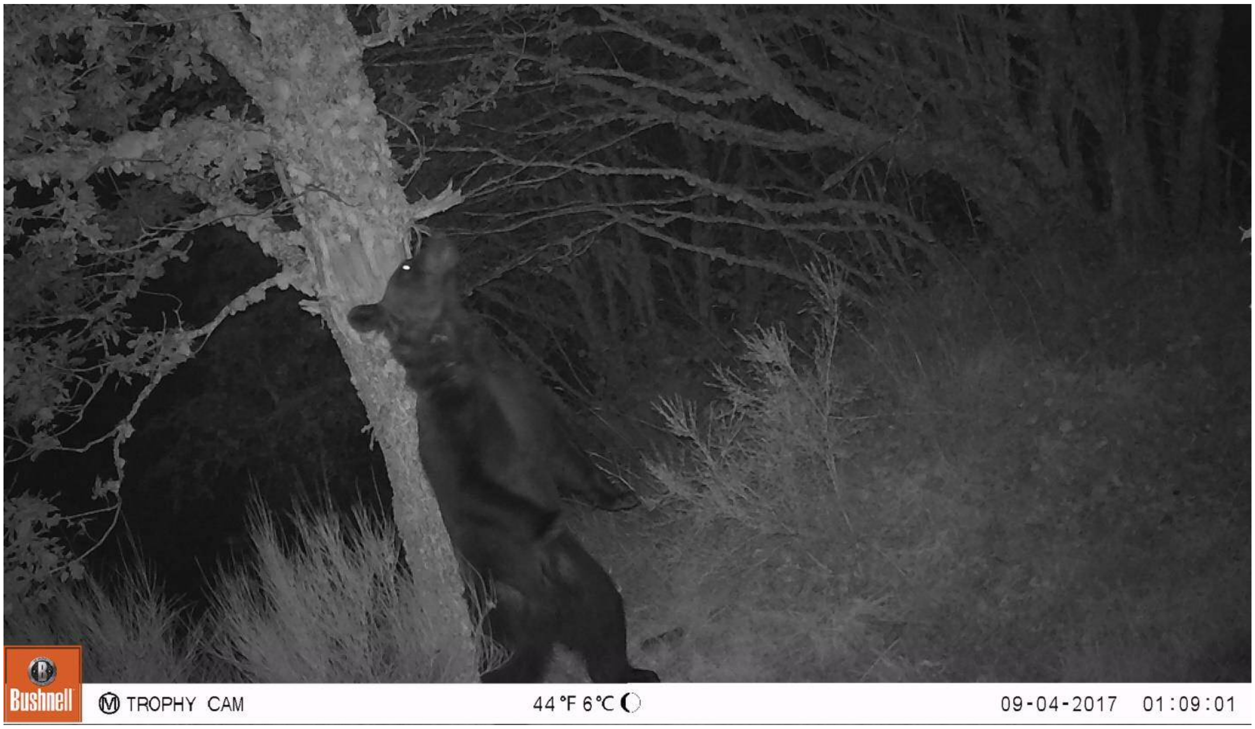
Bushnell
TROPHY CAM
44°F 6°C
09-04-2017 01:09:01

11.

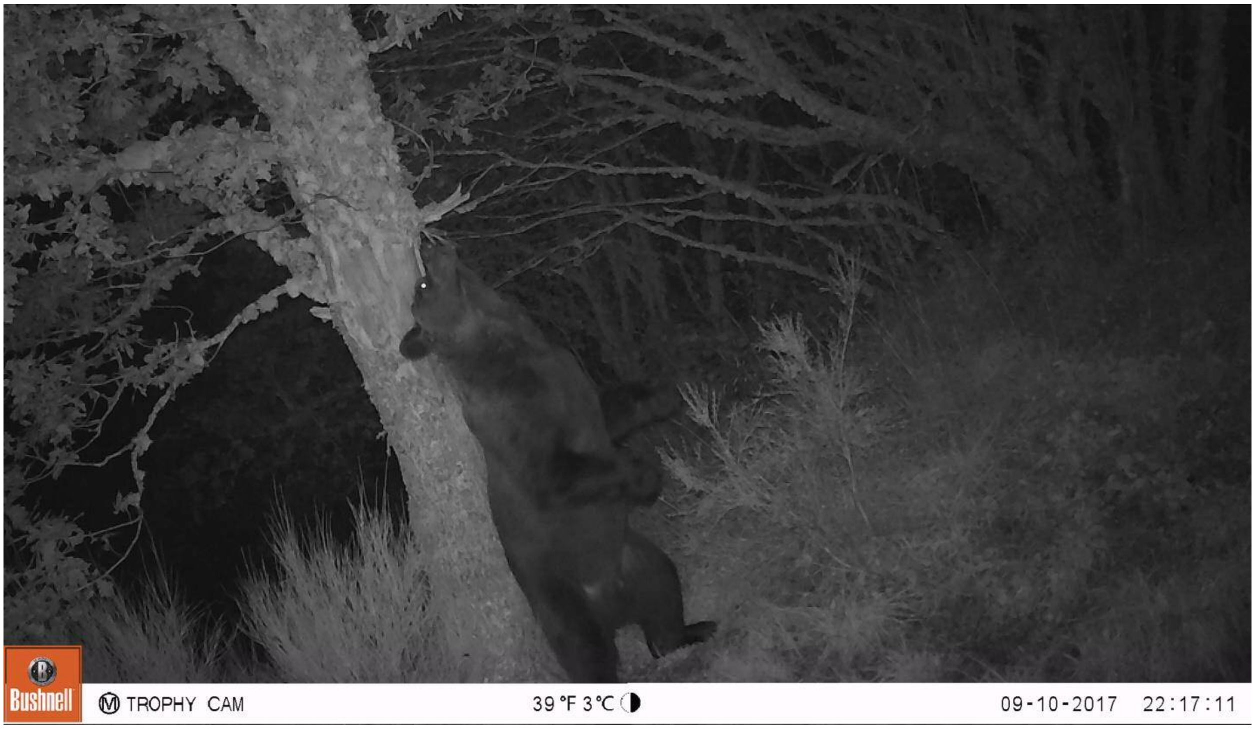
Bushnell
TROPHY CAM
39 °F 3 °C
09-10-2017 22:17:11

12.

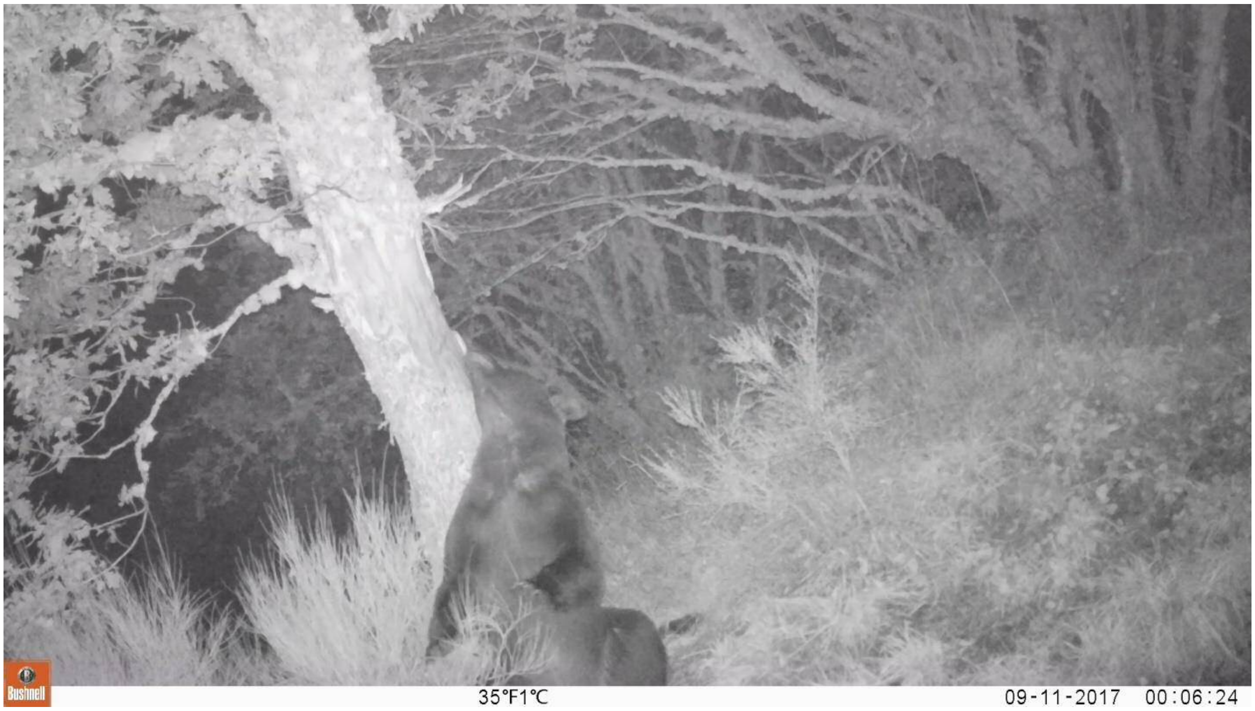
Bushnell
35°F1°C
09-11-2017 00:06:24

13.

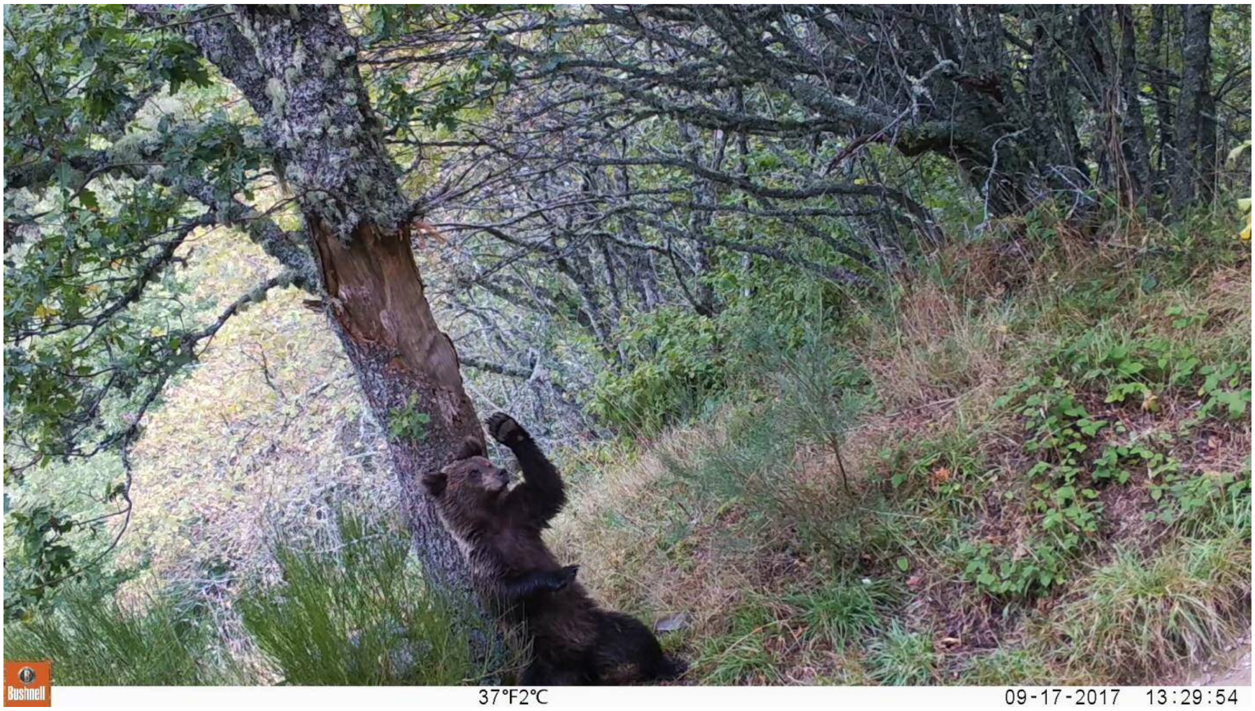
Bushnell
37°F2°C
09-17-2017 13:29:54

14.

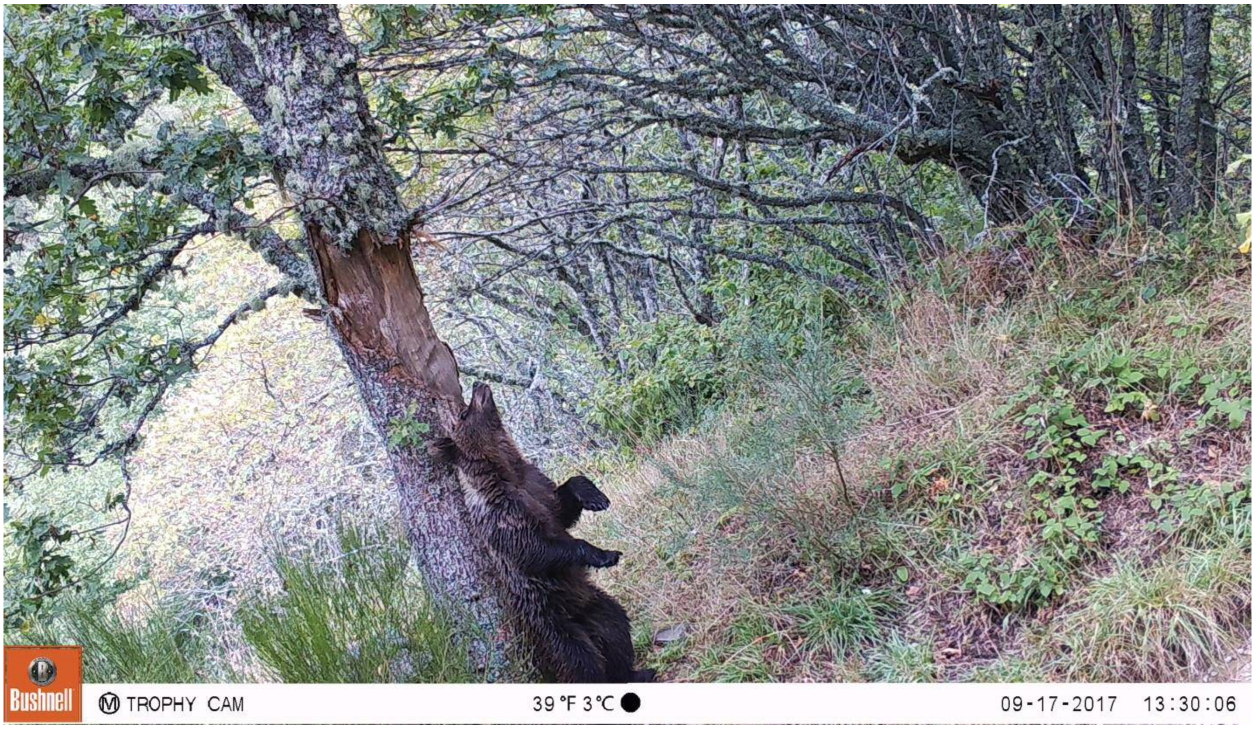
Bushnell
TROPHY CAM
39 °F 3 °C
09-17-2017 13:30:06
