# Supplementary material for: Visual marking in mammals first proved by manipulations of brown bear tree debarking
Source: Sci Rep. 2021 May 4;11:9492. doi: 10.1038/s41598-021-88472-5 (PMC8096968; doi:10.1038/s41598-021-88472-5)
Supplement: Supplementary file 12 — Supplementary Legends. [file 41598_2021_88472_MOESM12_ESM.docx]

**Extended Data Figures**

**Extended Data Fig. 1 | Fresh (i.e., current mating season) visual marks left by brown bears.** Different examples of fresh (few days to three months old) brown bear visual marks on diverse tree species and a touristic direction signpost. Colour differences between the outer bark and the interior of the tree (inner bark or sapwood) produce a bright contrast, which increases the conspicuousness of the visual signal.

**Extended Data Fig. 2 | Old (i.e., previous mating season) visual marks left by brown bears.** Different examples of old (> 1 year old) brown bear visual markings on diverse tree species. Even if the contrast between the outer bark and the interior of the tree (inner bark or sapwood) is less bright than for fresh marks (Extended Data Fig. 1), conspicuousness of the visual signal is still high. Indeed, the concealing of old marks with bark strips produced the same brown bear response as manipulations on fresh marks (Extended Data Fig. 7).

**Extended Data Fig. 3 | Visual signalling in brown bears (colour and black and white photos)**. 1. On the 29^th^ of April 2019 the tree had not yet been used for visual signalling by brown bears, even though it was a well-known rubbing tree. 2-6. On the 7^th^ of May 2019 an adult male scratched and bit the trunk to remove the outer bark and, thus, revealed the bright and conspicuous layer of inner bark. 7. An example of an adult male brown bear leaving a visual mark (light grey patch) on a tree. The bear is scratching the trunk with his left paw, first removing a rectangular strip of bark. 8. Always with the left paw, the bear increases the size of the mark. 9-11. By rising on its hind legs, the bear removes a new piece of bark with its mouth, resulting in the bark being completely separated from the trunk when the bear drops down.

**Extended Data Fig. 4 | Adult males visually marking trees (three videos)**. 1. Adult males leaving visual marks by scratching and biting bark on the upper portion of trunks during the mating period seems to be the most common scenario of visual signalling in brown bears. The evidence that the visual mark is produced after rubbing and on an upper section of the tree that is not reachable for chemical marking may support the possibility that visual signalling is additive and complementary to chemical signalling. 2. Again, during the mating period (16^th^ of May 2020), a new mark is being made by an adult male on a tree that already showed an older visual mark, which was done the previous year during the mating period (the clearest strip on the whole front part of the trunk). Marking is made by both biting and scratching the trunk. 3. Even if less frequently, visual marks can also be made at the base of the trunk. Here, a new mark appears (13^th^ of April 2020) at the base of a trunk that was already visually marked the year before. Chemical marking (i.e., rubbing and pedal marking) is also performed.

**Extended Data Fig. 5 | Bear removing manipulated bark strips concealing a visual signal (video)**. The first ever video footage of an adult male responding to the manipulation of bear visual marks (Cantabrian Mountains, NW Spain, 28/05/2020), ten days after manipulation of the visual mark, during the first visit of a bear to the tree. After chemically marking the ground (pedal marking, first 12 seconds), between seconds 18 and 23, after rubbing behaviour, the bear starts to remove some of the bark pieces that we used to cover the visual mark on the trunk of an ash tree. He uses both his claws and teeth. Then, at second 34, after continued rubbing, he removed the largest piece of bark covering the mark. Note that the visual signal is above the portion of the trunk where bear chemicals can be deposited by rubbing behaviour. The control bark strips on the same trunk as the visual signal (visible on the left side of the tree) are not removed. Similarly, when rubbing (from second 55 to the end of the video) on the closest tree (where there are no visual marks), the second control bark strips, i.e., the ones on the closest tree to the one with visual marks, are not removed. Control bark strips are visible on the left, lower side of the trunk, close to the hindquarters of the rubbing bear.

**Extended Data Fig. 6 | Example of the manipulation of bear marks by covering them with bark**. Several examples of brown bear visual mark manipulations are shown, where the mark on the trunk has been covered by strips of bark from the same tree species (1–16). 17a–17b: a marked tree (old mark) before and after manipulation (with detail of the manipulation in 17c). This is the tree where the manipulation of the mark has been removed in the Extended Data Figure 7. Details of before and after the manipulation of brown bear marks are also shown in paired photos 18a–18b, 19a–19b, 20a–20b (with detail of the manipulation in 20c). 21a–21d: an example of a removed manipulation (21a) and the remains of manipulated strips of bark at the base of the tree (pedal marking is also visible in 21d).

**Extended Data Fig. 7 | Removal of a bear mark manipulation covering an old visual signal**. From 1 to 10: the moment (end of May 2020) when a brown bear first rubs itself against the tree and then removes the bark (picture 10) that we used to cover an old visual mark (red circle in picture 4). Pictures 11 and 12 show the visual mark after the bear uncovered it. Picture 13 depicts a bear rubbing again one week later, suggesting that bark manipulations did not affect bear chemical marking behaviour.

**Extended Data Fig. 8 | Brown bear visual markings also occur on trees other than rub trees**. 1. A bark strip recently removed by a bear from a small birch trunk, which was found on the ground at the base of the trunk on the 2nd of May 2020. This tree was not used for rubbing. 2. Detail of the removed bark. 3-4. Details of the manipulation of the visual mark by covering it with the same bark strip removed by the bear. 5. The bark strip used to cover the visual mark had been removed between the 8th and the 15th of May 2020.

**Extended Data Fig. 9 | Brown bear visual marking is complementary to chemical signalling**. These images show one of the most typical features of brown bear visual marking, i.e., visual marks generally are on upper sections of the trunk, where even the largest adult males cannot leave their scent (chemical signalling) by performing rubbing behaviour. Here, different 'classes' of bears, from small subadults to large adult males mark (August-September 2017) the same tree, but only the adult male 'captured' by the camera trap in images 10 and 11 (and probably 12) can also cover part of the visual mark with its head when rubbing. This reinforces the possibility that: (1) visual marking is a prerogative of the largest and tallest bears (i.e., typically adult males); and (2) visual marks represent a marking behaviour additional and complementary to chemical marking, which may be distinctive to the largest individuals.
